# Understanding the unexpected effect of frequency on the kinetics of a covalent reaction under ball-milling conditions

**DOI:** 10.3762/bjoc.15.120

**Published:** 2019-06-05

**Authors:** Ana M Belenguer, Adam A L Michalchuk, Giulio I Lampronti, Jeremy K M Sanders

**Affiliations:** 1 Department of Chemistry, University of Cambridge, Lensfield Road, Cambridge CB2 1EW, UK; 2 BAM Federal Institute for Materials Research and Testing, Richard-Willstätter Str. 11, 12489 Berlin, Germany; 3 Department of Earth Sciences, University of Cambridge, Downing Street, Cambridge CB2 3EQ, UK

**Keywords:** ball-mill grinding, grinding frequency, kinetics, mechanism, mechanochemistry

## Abstract

We here explore how ball-mill-grinding frequency affects the kinetics of a disulfide exchange reaction. Our kinetic data show that the reaction progress is similar at all the frequencies studied (15–30 Hz), including a significant induction time before the nucleation and growth process starts. This indicates that to start the reaction an initial energy accumulation is necessary. Other than mixing, the energy supplied by the mechanical treatment has two effects: (i) reducing the crystal size and (ii) creating defects in the structure. The crystal-breaking process is likely to be dominant at first becoming less important later in the process when the energy supplied is stored at the molecular level as local crystal defects. This accumulation is taken here to be the rate-determining step. We suggest that the local defects accumulate preferentially at or near the crystal surface. Since the total area increases exponentially when the crystal size is reduced by the crystal-breaking process, this can further explain the exponential dependence of the onset time on the milling frequency.

## Introduction

We describe here an unusual frequency-dependence in the induction period of a covalent reaction carried out using ball-mill grinding. We present a kinetic analysis indicating that this is due to the successive fracture of crystals into smaller particles followed by the accumulation of energy in crystal defects. In recent years, manual and ball-mill grinding have become increasingly routine solid-state synthesis tools [1]. Generally referred to as mechanochemistry, these methods are more environmentally friendly and usually less expensive than traditional solution-based methods, because they require little or no solvent. Moreover, mechanochemical syntheses often give quantitative yields of products [2–4]. Manual or mechanical grinding can be performed “neat”, i.e., in the absence of solvent (neat grinding, NG). Alternatively, very small quantities of liquid can be added to the grinding mixture [5], a procedure known as "kneading" or "liquid-assisted grinding" (LAG) [4]. The liquid often accelerates reactions between solids or even enables new reactions [5–7]. Mechanochemical methods have been successfully applied for a wide range of different syntheses and chemical reactions of inorganic [8–9] and organic [10–11] compounds. Even supramolecular architectures such as co-crystals and metal-organic frameworks [4,12–14], cages [15] and rotaxanes [16] could be formed mechanochemically. Crucially, the mechanisms and driving forces which underpin mechanochemical transformations and supramolecular reactions remain poorly understood and are subject to considerable debate [2,4,7–8,14,17–25]. The future successful academic and industrial application of these methods depends on developing a fundamental understanding of these solid-state processes.

The validation of reaction kinetic models has been a powerful approach for investigating fundamental processes in chemistry and physics. This has led to significant advancement in the understanding of molecular and submolecular phenomena. A number of researchers have attempted to rationalize organic mechanochemical transformation profiles in a similar way [26–28], with far more developed with respect to inorganic reactions [29–36]. However, despite many advances in developing mathematical models based on various kinetic treatments, an understanding of mechanochemical reaction dynamics remains largely elusive. While mechanochemical kinetics must obey the general principles of reactivity (collision, energy gain and relaxation), there remains a poorly understood, complex interplay between physical and chemical phenomena [37], which are not captured in traditional fluid-phase kinetics treatments. Furthermore, many physical parameters are intimately coupled (e.g., milling-ball size and mass), and carefully designed studies are required to understand their independent effects on the reaction rate [29,38–43]. Hence, before one can develop elementary kinetic equations for these processes, it is crucial to understand the types of processes that must be independently considered.

**Scheme 1:**
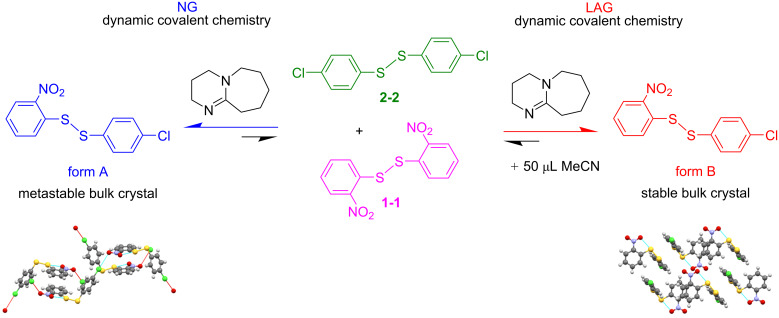
Solid-state exchange reaction through ball-mill grinding under neat ball-mill-grinding conditions (left) and under ball-mill LAG conditions (right). From the solid-state reaction of the homodimers (CCDC [48] codes ODNPDS02 and DCPHDS for **1-1** and **2-2**, respectively) only the relevant stable polymorph of the heterodimer, form A and form B respectively, crystallizes. The details of form A (CCDC code FUQLIM01) and form B (CCDC code FUQLIM) have been previously reported.

Recently, we have been investigating the final reaction equilibrium achieved under ball-mill LAG conditions [17–18,44]. It is generally accepted that when the milling reaction reaches completion in a sealed system, a steady state is eventually achieved. The final phase composition does not change as long as the milling conditions are maintained [1,17–18,45–46]. Such equilibria depend on numerous factors, including ball-mill jar size, shape and material, ball-bearing size, weight and material, milling frequency, temperature, and the nature and concentration of the added liquid [17–18]. In this paper we investigate how the ball-mill grinding frequency affects the kinetics of the covalent reaction of bis(2-nitrophenyl) disulfide and bis(4-chlorophenyl) disulfide in the presence of a small amount of the base catalyst 1,8-diazabicyclo[5.4.0]undec-7-ene (DBU) to produce 4-chlorophenyl 2-nitrophenyl disulfide (see Scheme 1). Reliable experimental procedures have already been established for this system [47]. While the experimental operations for the system are given below and in Supporting Information File 1, we refer to our methodology paper for further and more general details and considerations [47].

The results presented here show a significant induction time before the reaction starts. We interpret this as a consequence of a two-stage process: a first stage that is dominated by crystal breaking, and a second stage in which the energy supplied by the ball-bearing impact is stored as structural defects (within crystalline or cohesive states [37] at the molecular level). Indeed, it has been suggested that a number of mechanochemical transformations depend greatly on the accumulation of energy [29,49–51]. We therefore propose that this energy accumulation is the rate-determining step: when a certain threshold is overcome the reaction starts very suddenly and occurs rapidly. The idealized model presented here serves as proof-of-concept for an often-overlooked aspect of the coupling between physical and chemical phenomena, required to rationalize the unconventional kinetics associated with mechanochemical transformations.

## Results and Discussion

**Figure 1:**
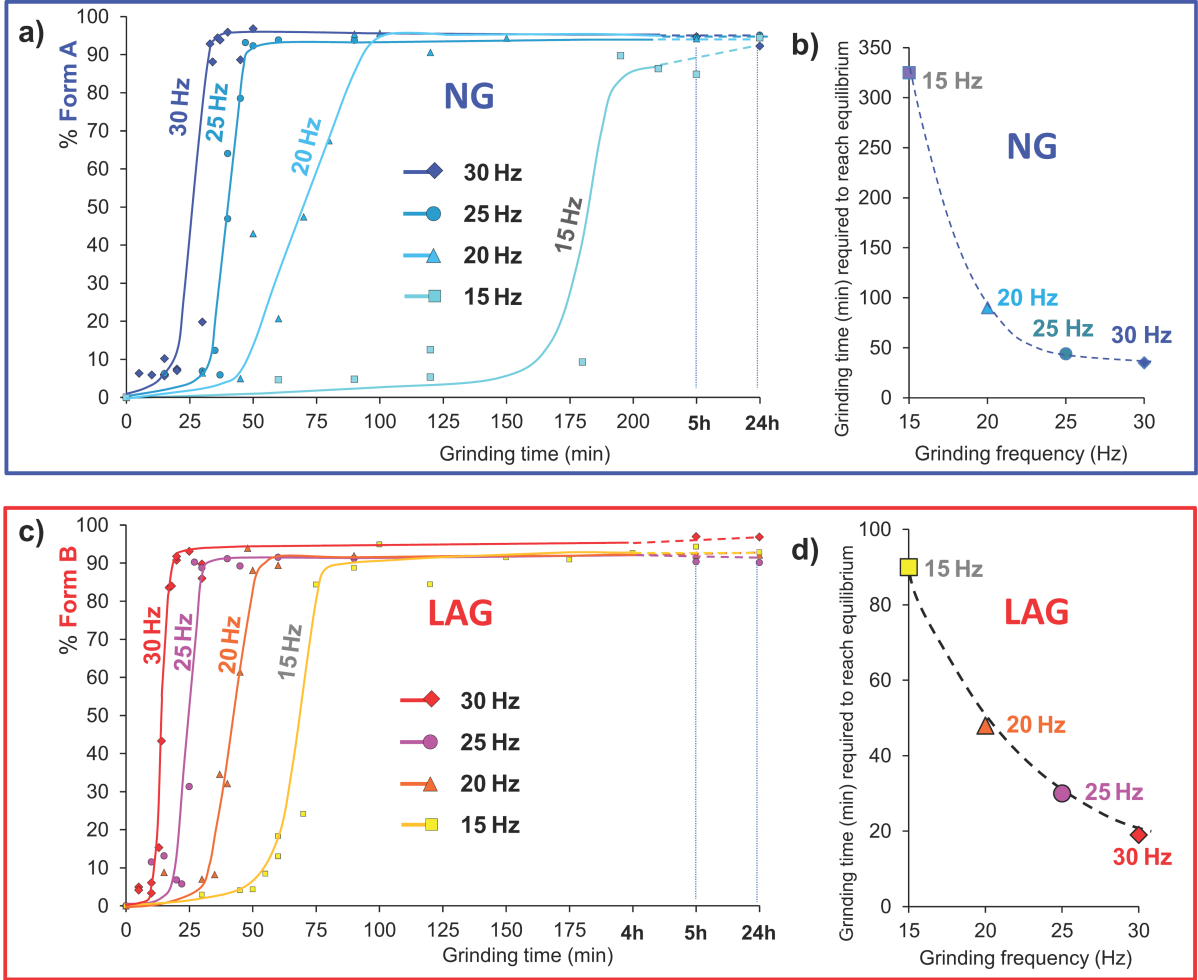
Solid-state studies reacting **1-1** and **2-2** in an equimolar ratio in the presence of DBU as catalyst to give the heterodimer **1-2**. (a) And (c) show the dependence of reaction progress (obtained by the Rietveld quantitative phase analysis) on milling frequency under ball-mill NG and LAG conditions (with 50 μL of MeCN), respectively. No fitting was performed, and the kinetic curves are only a guide to the eye. Each time point in these kinetic plots corresponds to a separate grinding experiment.

Figure 1 show the experimental results for the progress of product formation as a function of grinding time.

Purposely designed stability experiments of the ball mill NG and LAG reactions were conducted by interrupting the milling experiments of **1-1** + **2-2** + DBU at different times and storing the grinding jars sealed. After several months and up to years, these sample jars were opened and the materials reanalyzed by HPLC in order to obtain their chemical composition. We observed that the nanocrystals of **1-1** and **2-2** homodimers had reacted during storage in the absence of further mechanochemical activation, thereby resulting in an increased concentration of **1-2**. This increase in **1-2** was only observed with aged NG and LAG samples that contained more than 2 mol % of **1-2** seeds. Higher concentrations of **1-2** seeds in the initial sample led to a higher overall conversion with aging. Importantly, we stress that the detectable increase in **1-2** with time required a period of months, or even years. Hence, the kinetics that we observe in the experiments reported in this work pertain only to the mechanochemical phenomena themselves.

The rate (*r*) associated with any physical or chemical transformation can be described by generic Equation 1

[1] r∝f(α)×A×exp(−E/RT)

where *A* × exp*(−E/RT)* is the Arrhenius-type rate constant, and *f*(α) is the functional form of rate, dependent on the specific mechanism of the transformation. Many of the traditional kinetic equations for solid-state transformations (e.g., the Avrami–Erofeyev and Prout–Tompkins models) are derived for single phase solid-state transformations [52]. Their general application to multiphase mechanochemical reactions is thus limited.

**Scheme 2:**
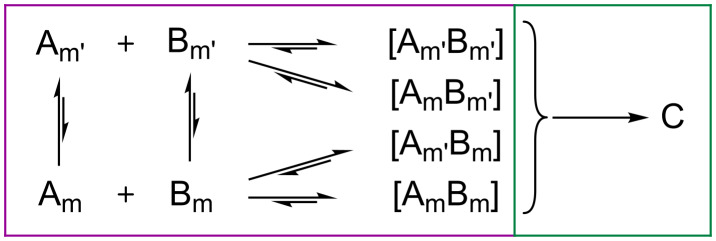
Schematic representation of a solid + solid mechanochemical reaction. Subscript denote macroscopic (m) and comminuted (m’) particles.

A general mechanochemical reaction can (macroscopically) be taken to consist of two stages: (1) mass transport (i.e., mixing) and formation of heterogeneous, reactive contacts and (2) activation of these contacts by mechanical impact. This is analogous to traditional chemical kinetics, limited by collision and activation. Macroscopically, a general irreversible mechanochemical reaction reaching equilibrium can be thus considered according to Scheme 2. A notable assumption is that all combinations of the physical complex [AB] yield the same product phase. While the latter is not strictly true in all cases [28], it does hold for the general case. The equilibria between complex [AB] and the pure components represent de-mixing, as well as surface regeneration by expulsion of C.

**Scheme 3:**
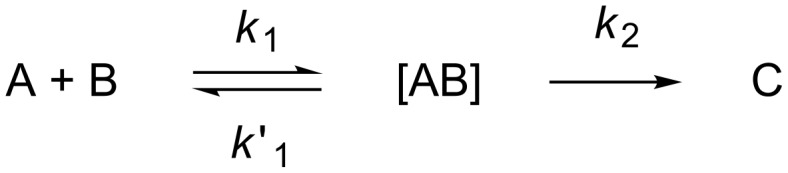
Simplified reaction equation for the mechanochemical transformation. Note that [AB] is a physical complex.

In such a scheme, it is irrelevant which intermediate state forms, and thus the kinetic constant for reactive contact formation represents an average of all such states. Hence, Scheme 2 can be drastically simplified to Scheme 3.

The kinetic profiles presented in Figure 1 (b and d) exhibit striking non-linearity in their dependence on the milling frequency. Furthermore, they each exhibit sizeable induction periods, far greater than most reported kinetic profiles of multi-phase mechanochemical non-covalent supramolecular chemical reactions [28,45]. The notable exceptions are similar synthetic reactions, which induce covalent bond formation, conducted by mechanochemistry [46]. A large induction period can be explained by two mechanisms: (1) time required for mass transport (mixing), or (2) time required for the accumulation of energy. While mass transport surely plays some role in the observed induction period, it requires that no reaction occurs for several tens of minutes, followed by a sudden onset of covalent chemistry. This does not appear likely. Furthermore, this induction period, with sudden and rapid onset of reaction, is not typical of most solid-state kinetic models, but is instead typical of temperature (energy)-dependent kinetics in which a reaction does not occur until sufficient energy is present in the system [53].

Instead, it has been suggested that the kinetics in covalent mechanochemical transformations depend greatly on the accumulation of energy, which can be stored as local defects, or trapped within the energetic framework of the submolecular system [29,54–55]. However, the latter is not expected to contribute substantially to macroscopic mechanochemical phenomena, assuming typical relaxation times in the order of microseconds or less [29,56]. Macroscopically, energy can also accumulate as heat, which itself is known to have an effect on the rate of the mechanochemical transformation [23]. This accumulation is taken here to be the rate-limiting step. For the purpose of this proof-of-concept study, we do not consider any particular energy accumulation (or relaxation) pathway. Instead, only the total of all phenomena is considered. In reality, this accumulation and subsequent relaxation is highly complex, involving submolecular (electronic and vibrational) effects, as well as defect generation and temperature development [54,57].

A rate constant, *k*_2_, is hence developed as a modification from that originally proposed by Butyagin [29], in which the rate is proportional to the frequency of collision (A), and the initial activation energy (*E*_0_) for the chemical reaction. For systems in which the reaction is limited by the distribution of mechanical energy, it has been suggested that the temperature term of the traditional Arrhenius equation can be (to a first approximation) replaced by the rate of supply of mechanical energy, W [29], (Equation 2)

[2] $$k_{2}=A\times \exp\left(-\frac{E_{0}-E_{acc}}{W}\right)$$

Hence, *k_2_* represents the ‘per impact’ probability of reaction. We note that the individual terms of Equation 2 are indeed expected to be dependent on the equilibrium temperature of the system, although this dependence is outside the scope of the present manuscript. With the rate of energy relaxation, τ, normalized to 1 Hz, the energy accumulation can be approximated (see Supporting Information File 2 and Equation 3),

[3] $$E_{acc}=\{\begin{array}{c} W(\nu -\tau );\nu /\tau \geq 1\\ 0;\nu /\tau <1 \end{array}$$

thus denoting the accumulation of energy at a rate proportional to the difference in milling frequency ν (Hz) and the relaxation of energy τ (Hz). We note that this is a somewhat simplified form, not considering the differences between the mechanism for energy accumulation. Furthermore, this simplification assumes a linear increase in stored energy throughout the bulk material. Note that all attempts at scaling energy accumulation resulted only in shifting of the relative onset times (Supporting Information File 2).

If it is assumed that *k*_1_, *k*_1_' 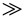 *k*_2_, the rate equation for the transformation can be expressed as in Equation 4:

[4] $$f(\alpha_{\text{C}}(t))=\alpha_{\text{AB}}(t)\exp(-k_{2}t)$$

The energy of an ideal impact can be worked out from the classical equations of motion, assuming an ideal linear trajectory of the milling ball along the entire length of the milling jar at the acceleration of the mill. Note that we have made the assumption that ‘double-impacts’ do not occur and that the two milling balls simultaneously impact the powder directly. This has a similar effect as simply modelling a milling ball with a larger surface area [38]. Double impacts, and ball–ball collisions are certainly important considerations for future development of this simple model. The resulting impact energies, *W*, are given in Table 1, and it is assumed that the milling ball is subsequently ejected at the same velocity after impact. Unfortunately, Equation 2 cannot be solved without knowledge of *E*_0_ and *τ*. However, the relative rate is proportional only to *E*_0_:*τ* (Supporting Information File 2), which we selected here to fit the experimental curve for 15 Hz ball-mill neat grinding.

**Table 1:** Approximate kinetic energy of an ideal impact of a 1.43 g milling ball at different milling frequencies. The relative frequency is shown in each case. Note that the milling jar oscillation distance was measured at 4 cm.

Frequency, ν	*W* /mJ

15 Hz	0.489
20 Hz	0.870
25 Hz	1.359
30 Hz	1.958

**Figure 2:**
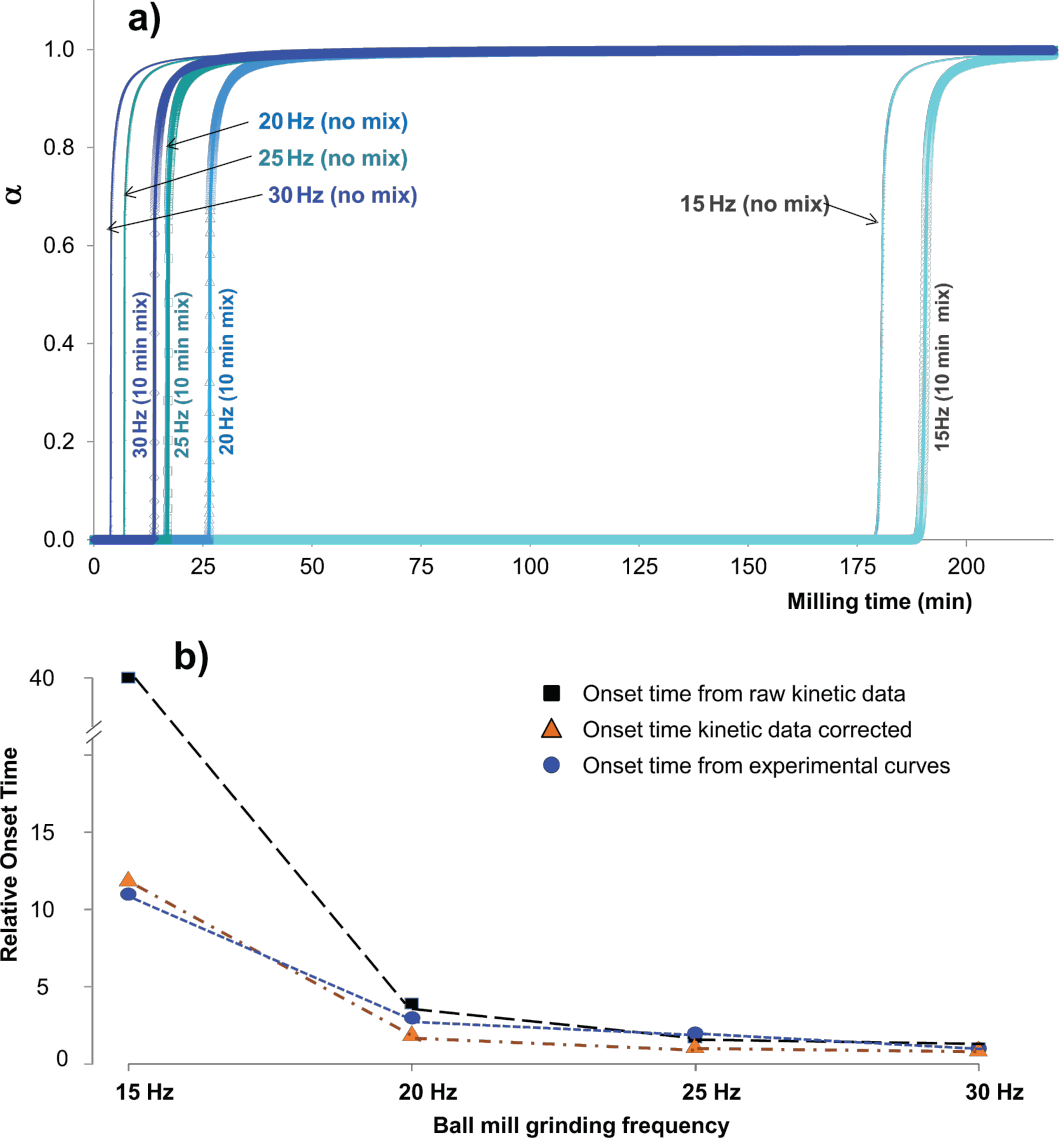
Reaction profiles for mechanochemical milling according to Equation 4. (a) Kinetic profiles for 30 Hz, 25 Hz, 20 Hz and 15 Hz NG milling. Raw (fine line curves) and corrected (base onset time + 10 min; thick line curves) are given and labelled. Onset time (normalized relative to 30 Hz model data) correction approximated from experimental curves. (b) Comparison of relative conversion onset times from raw kinetic curves (black rectangles), onset time corrected (brown triangles), and from experimental ball mill NG curves (blue circles) in Figure 1. *E*_0_:*τ* = 17.48.

If a model is built on Scheme 2, with *k*_1_, *k*_1_' 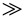 *k*_2_, Figure 2A is generated. Remarkably, despite the simplifying assumptions used in this model, the general features of the experimental curve are well reproduced. Based solely on the consideration of the physical parameters *W* and ν, the significant non-linearity observed in experiment is obtained, with the onset time *t*_on_ decreasing as *t*_on_ (15 Hz) >>> *t*_on_ (20 Hz) >> *t*_on_ (25 Hz) > *t*_on_ (30 Hz). The on-set time is taken as the point where the tangent to the pre-accumulation plateau meets that of the accumulation curve. Although the model explains the general features, it cannot capture the resolution in onset frequencies for the three fastest milling reactions. We believe this to be due to the crude assumption of ideal impact trajectory; non-ideal trajectory should decrease the energy accumulation and hence elongate the induction period.

If the raw values are used, it is found that the relative rate of conversion for the 15 Hz milling reaction is substantially overestimated with respect to the experiment. However, we note that the present model assumes energy accumulation beginning immediately, and does not allow for initial comminution or mixing, as is required [58]. The discrepancy in the 15 Hz milling onset time may represent an effect of the approximate mixing and comminution rate. Indeed, specimens taken from different areas of the milling jar showed significant compositional differences even after 75 minutes (see Supporting Information File 1). The re-normalized onset times predicted by the model match very well the ones observed experimentally (Figure 2b).

The generation of the observed kinetic profiles requires a consideration of the energy accumulation. If energy is not permitted to accumulate, each individual impact is too small to induce a chemical transformation. If each impact is taken to be sufficient for chemical reaction, then the entire induction period must be taken to be the result of mass transport, in which case slow, gradual kinetics would be expected. Unfortunately, the need to account for energy accumulation does not permit the assessment of *E*_0_ for the present system.

**Figure 3:**
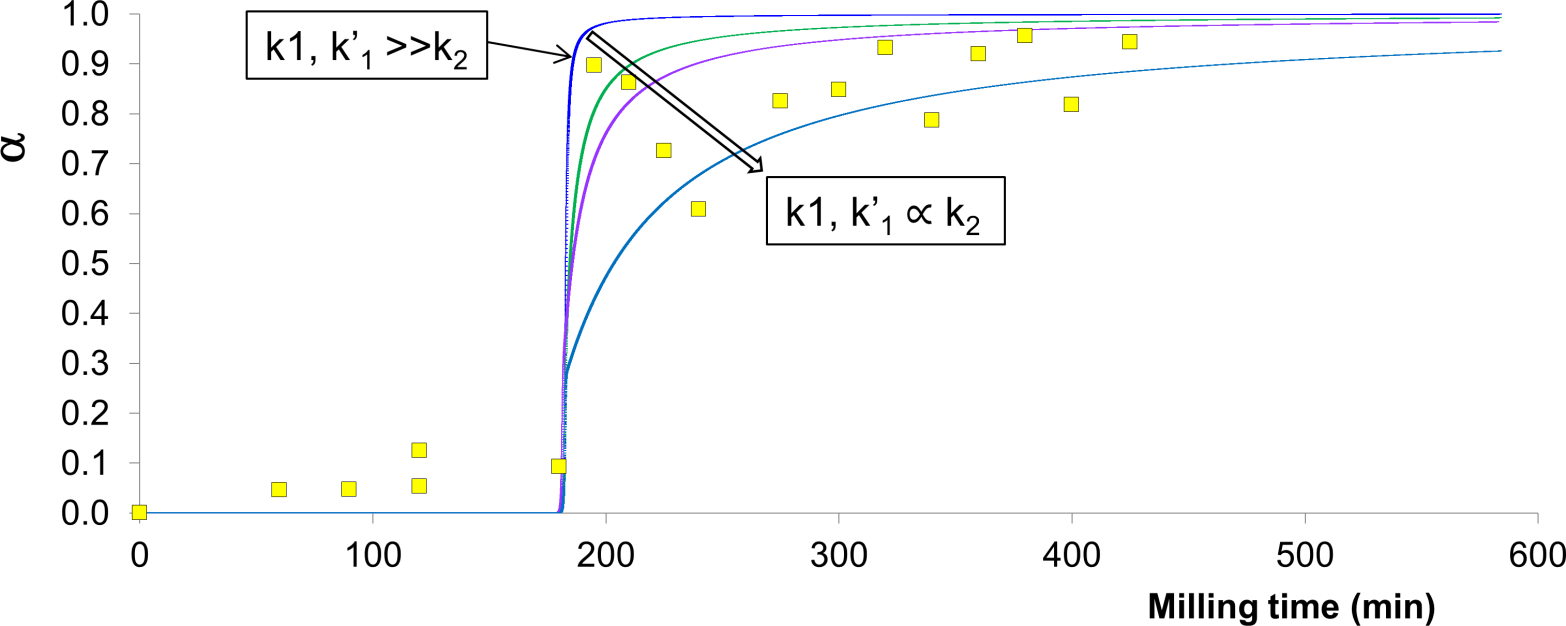
Modelled kinetic profiles for 15 Hz neat milling, with variation in the magnitude of the mixing term. Experimental data points for NG at 15 Hz are provided as yellow squares. Note that no parameters in the model are fit from experiment.

In our attempts to model Scheme 2 using *k*_1_,*k*_1_' 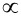 *k*_2_, no noticeable effect could be observed on the onset time. Instead, it was found that the relative mixing rate affects the slope of accumulation (Figure 3). By comparison to the experimental curves in Figure 1, this effect appears to dominate in the 20 Hz and 15 Hz profiles. This suggests that on the macroscopic scale, lower frequency milling is limited not only in the rate of energy input, but also by its ability to facilitate mass transport.

**Figure 4:**
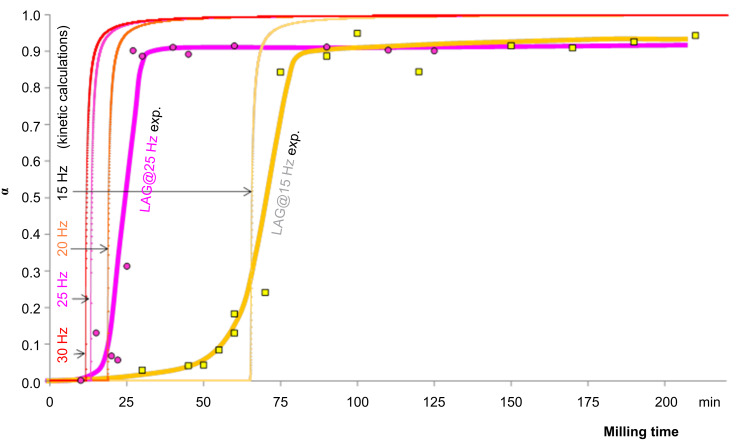
Reaction profiles for LAG mechanochemical milling according to Equation 4. The modelled curves are given for each milling frequency, and experimental data points are provided for 25 Hz (pink circles) and 15 Hz (yellow squares). No fitting was performed for the experimental data and the kinetic curves drawn are only a guide to the eye. *E*_0_:τ = 19.23

Having ascertained that the model of Equation 4 well describes the non-linear kinetics observed for neat grinding, it is worth considering this model with respect to LAG kinetics. While the non-linearity is not so evident under LAG conditions, the consideration of the relative onset rate does suggest its presence, albeit shifted towards lower grinding times. This is captured by reducing the *E*_0_:τ ratio in the kinetic model (Figure 4). The general structure of the experimental curves is reproduced well, with the 15 Hz profile considerably higher in onset time than the others. Unfortunately, it seems that again the three fastest milling frequencies are clustered somewhat too closely. One important aspect that our model does not take into account is the crystal size and the relative surface-to-volume ratio. The latter could explain an exponential dependence of the onset time on the frequency, as the specific area increases exponentially with the reduction of crystal size. For the specific area to affect the onset time, the local submolecular defects responsible for the energy accumulation should cluster preferentially at or near the crystal surface [59]. Our relatively simple model does not consider the non-ideal ball trajectory and this may play a role. Further work is therefore required to account for these important aspects.

While we are cautious about deriving mechanistic information from this result, it suggests that LAG primarily lowers the relative activation energy of mechanochemical reactions or facilitates the energy accumulation when compared to experiments run under ball mill NG conditions. These two explanations are not mutually exclusive. While the activation may be facilitated by partial dissolution of components, it is possible that crystallite aggregation plays a significant role under NG conditions: the energy supplied by the impact would therefore be used to break down aggregates as well as crystallites, the aggregation itself being limited by the MeCN solvent under LAG conditions. Indeed, aggregation seems to be a rather important phenomenon in the reaction kinetics under mechanochemical conditions [59].

## Conclusion

The kinetic profiles observed for neat and LAG processes described in this work are anomalous when compared to traditional solid-state processes. They are characterized by lengthy induction periods and a sudden, rapid conversion to the product phase. Traditional equilibrium kinetics demonstrates such behavior when insufficient energy is initially present in the system. Unique to mechanochemical transformations, however, is the periodic nature of the input energy. A simple model was therefore employed to account for this periodicity. Remarkably, the abnormal kinetic behavior of the system was captured within such a model, requiring only two physical parameters from the milling system: milling frequency and ideal impact energy. While induction periods in mechanochemistry can be the result also of mixing, these effects are considerably smaller. For such an effect to dominate in the present case, one must assume that no reaction takes place until ideal mixing is achieved, which is unrealistic. Indeed, the inclusion of a mixing term does not affect the onset time for reaction. However, it was found that the inclusion of a mixing term does lead to ‘shaping’ of the accumulation profile. Such effects appear necessary to capture the kinetic profile of the lower frequency kinetic curves. This suggests that, while onset time is dependent on frequency and input energy, the mixing (mass transport) can dominate subsequent stages of the transformation, as the probability of contact formation decreases. While further work is required to capture detailed mechanistic insight, we can suggest that kinetic modelling of covalent mechanochemical reactions likely requires a model that accounts for both the accumulation of energy and mixing effects. The induction time is significantly shorter under LAG conditions. This can be explained by either a lower activation energy under LAG conditions, or aggregation playing a more important role under NG conditions. The crystal breaking process is likely to be dominant at first, and it involves breaking crystallites as well as crystallite aggregates. When the particles are reduced in size, the energy supplied is stored at the molecular level as local crystal defects. This accumulation is taken here to be the rate-determining step. We suggest that the local defects accumulate preferentially at or near the crystal surface. Since the total area increases exponentially when the crystal size is reduced by the crystal breaking process, this can further explain the exponential nature of the onset-time dependence on the milling frequency.

Milling reaction kinetics is a relatively unexplored field, and we have explored only one reaction, but it seems likely that similar effects will operate for other reactions.

## Experimental

The kinetic studies presented here were performed under ball-mill neat grinding (NG) and under ball mill liquid-assisted grinding (LAG) conditions with 50 μL of acetonitrile added to 200 mg of powder. The kinetic points prepared for this study are all single point experiments. The reaction under study is a base-catalyzed disulfide exchange reaction starting from equimolar amounts of homodimers using DBU as the base catalyst to result in the formation of the heterodimer. The homodimers (0.34 mmol) bis(2-nitrophenyl) disulfide (**1-1**, 104.83 mg) and bis(4-chlorophenyl) disulfide (**2-2**, 97.66 mg) were accurately weighed, resulting in a load of 200 mg. The material was quantitatively transferred to a 14.5 mL snap closure stainless steel grinding jar and two 7.0 mm in diameter stainless steel balls were placed on top of the powder. Then, 2 μL (2 mol %) of the base catalyst 1,8-diazabicyclo[5.4.0]undec-7-ene (DBU) were carefully added on top of the milling balls. For NG experiments nothing else was added while for LAG experiments, 50 μL of acetonitrile were added on top of the powder. The grinding jars were snap-closed, the closure secured with insulating tape milling was conducted at 15–30 Hz using a MM400 Retsch automated grinder for the specified period of time (see Figure 1 and Supporting Information File 1). The grinding jars were opened immediately after completion of the grinding period; the PXRD sample prepared on a slide and then scanned ex situ by PXRD as soon as possible so as to get the most reliable data. HPLC analysis to obtain the chemical composition of the sample was performed as soon as possible and always within the same day, reported as mol % and documented in Supporting Information File 1. The solid product was dissolved in MeCN + 0.2% trifluoroacetic acid (TFA) at a concentration of 1 mg/mL and injected in the HPLC system. TFA was added to the sample for HPLC analysis to neutralize the base DBU and to quench the disulfide exchange reaction. The results of the milling experiments were plotted as mol % concentration of phase composition versus grinding time (see Figure 1, Figure 4 and Supporting Information File 1). The phase composition is calculated from the Rietveld refinement of the PXRD scans. The estimated accuracy of the phase composition by Rietveld refinement is ±3 mol % of the absolute and estimated sensitivity while the limit of detection (LOD) is 3 mol %. The estimated sensitivity of the HPLC analysis resulting in the chemical composition of the samples is 0.1 mol % relative to the main component. Therefore, while the PXRD analysis is not as sensitive or accurate as the HPLC analysis, it supplies the phase composition. There is an excellent agreement between the phase composition obtained by PXRD and the chemical composition obtained by HPLC (see Supporting Information File 1). Additional details about the analytical PXRD and HPLC methods can be found in Supporting Information File 1.

A sufficient number of independent kinetic milling points were performed so as to obtain a good resolution of the sigmoidal segment of the kinetic curves and to demonstrate that the milling reaction had finally reached a plateau. To achieve this level of accurate and reproducible kinetic profiles, rigorous experimental procedures detailed in Supporting Information File 1 and in reference [47] were found necessary.

## Supporting Information

File 1 Experimental methodology for ball mill grinding experiments, analysis by HPLC and PXRD; quantitation by Rietveld refinement and particle size analysis by Scherrer equation.

File 2 Kinetic model parameterization and additional model features.
