# Supplementary material for: Understanding the unexpected effect of frequency on the kinetics of a covalent reaction under ball-milling conditions
Source: Beilstein J Org Chem. 2019 Jun 5;15:1226–35. doi: 10.3762/bjoc.15.120 (PMC6604707; doi:10.3762/bjoc.15.120)
Supplement: File 1 — Experimental methodology for ball mill grinding experiments, analysis by HPLC and PXRD; quantitation by Rietveld refinement and particle size analysis by Scherrer equation. [file Beilstein_J_Org_Chem-15-1226-s001.pdf]

BEILSTEIN JOURNAL OF ORGANIC CHEMISTRY

Supporting Information

for

Understanding the unexpected effect of frequency on the kinetics of a covalent reaction under ball-milling conditions

Ana M. Belenguer, Adam A. L. Michalchuk, Giulio I. Lampronti and Jeremy K. M. Sanders

Beilstein J. Org. Chem. **2019,** *15,* 1226–1235. doi:10.3762/bjoc.15.120

Experimental methodology for ball mill grinding experiments, analysis by HPLC and PXRD; quantitation by Rietveld refinement and particle size analysis by Scherrer equation

License and Terms: This is a supporting information file under the terms of the Creative Commons Attribution License (http://creativecommons.org/licenses/by/4.0). Please note that the reuse, redistribution and reproduction in particular requires that the authors and source are credited.
The license is subject to the *Beilstein Journal of Organic Chemistry* terms and conditions: (https://www.beilstein-journals.org/bjoc)

Table of Contents

1 Experimental section: materials and equipment S4
1.1. Materials S4
1.1 Equipment S4
1.1.1 Ball mill grinder S4
1.1.2 Automated solenoid to press the Retsch MM400 Shaker Mill start button S4
1.1.3 Grinding jars S5
1.2 Balance S5
1.3 Automatic pipettes S5
1.4 HPLC equipment S6
1.5 PXRD equipment S6
2 Experimental section: general concepts S6
2.1 Dynamic covalent chemistry S6
2.2 Solid state dynamic covalent chemistry S6
3 Experimental section: methodology S7
3.1 Preparation of single point experiments for kinetic studies of the solid-state DCC reaction of 1-1 and 2-2 under NG and LAG S7
3.1.1 Physical appearance of the product of ball mill grinding reaction S7
4 Experimental section: analysis of the solid-state samples S8
4.1 Analysis by HPLC S8
4.1.1 Preparation of HPLC samples from solid-state DCC studies S8
4.2 Analysis by PXRD S8
4.2.1 Sample preparation for PXRD analysis S9
4.3 Rietveld quantitative analysis of solid-state samples S9
4.3.1 Rietveld quantitative analysis S10
4.3.2 Crystal size determination from powder diffraction data S11
5 Experimental section: kinetic studies S13
5.1 Tabulation of the locations of the data used for kinetic studies S13
5.2 Kinetic studies of ball mill LAG of 1-1 and 2-2 with 2 mol % dbu S13
5.3 Kinetics LAG studies of 1-1 and 2-2 with 2 mol % dbu at 30 Hz S13
5.4 Kinetics LAG studies of 1-1 and 2-2 with 2 mol % dbu at 25 Hz S19
5.5 Kinetics LAG studies of 1-1 and 2-2 with 2 mol % dbu at 20 Hz S24
5.6 Kinetics LAG studies of 1-1 and 2-2 with 2 mol % dbu at 15 Hz S29
5.7 Lack of homogeneity in LAG at low grinding frequency (15 Hz) S34
5.8 Comparison between kinetics LAG studies at 30, 25, 20 and 15 Hz S36

6 Kinetic studies of ball mill NG of 1-1 and 2-2 with 2 mol % dbu ... S37
6.1 Kinetics NG studies of 1-1 and 2-2 with 2 mol % dbu at 30 Hz ... S37
6.2 Kinetic NG studies of 1-1 and 2-2 with 2 mol % dbu at 25 Hz ... S43
6.3 Kinetic NG studies of 1-1 and 2-2 with 2 mol % dbu at 20 Hz ... S48
6.4 Kinetic NG studies of 1-1 and 2-2 with 2 mol % dbu at 15 Hz ... S53
6.5 Proof of homogeneity in NG at low grinding frequency (15 Hz) ... S58
6.6 Comparison between kinetics NG studies at 30, 25, 20 and 15 Hz ... S59
7 Literature ... S61

Nomenclature and abbreviations used

DCC	Dynamic covalent chemistry (see section 2.1)
Solid state DCC	Solid state dynamic covalent chemistry (see section 2.2)
DCL	Dynamic combinatorial library: library resulting from DCC
LAG	Refers to ball mill liquid assisted grinding. The term “LAG” is equivalent and assumes we are discussing ball mill LAG
NG	Refers to ball mill neat grinding. The term “NG” is equivalent and assumes we are discussing ball mill neat grinding
1-1	Refers to the homodimer $(2NO_2PhS)_2$ or bis(2-nitrophenyl) disulfide
2-2	Refers to the homodimer $(4ClPhS)_2$ or bis(4-chlorophenyl) disulfide
1-2	Refers to heterodimer $2NO_2PhSSPh4Cl$ regardless of the polymorphic form<br>1-2 = Form A + Form B
Form A	Refers to the polymorph of $(2NO_2PhSSPh4Cl)$ obtained typically from NG:<br>CSD refcode FUQLIM01.
Form B	Refers to the polymorph of $(2NO_2PhSSPh4Cl)$ obtained typically from LAG (50μL MeCN): CSD refcode FUQLIM.
dbu	1,8-Diazabicyclo[5.4.0]undec-7-ene (base catalyst)
MeCN	Acetonitrile
TFA	Trifluoroacetic acid
FA	Formic acid
HPLC	High performance liquid chromatography
PXRD	Powder X-ray diffractometry
ID	Internal diameter
Hz	Hertz (frequency used to swing the grinding jars by the ball mill grinder)
h	Hours
m	Minutes
GC	Gas chromatography

1 Experimental section: materials and equipment

1.1. Materials

All solvents used for ball-mill grinding experiments were obtained as follows: acetonitrile (MeCN, HPLC grade) from Fisher Scientific.

All disulfide starting materials and reagents used in the solid-state DCC experiments were purchased from commercial suppliers: 1,8-Diazabicyclo[5.4.0]undec-7-ene (dbu) [6674-22-2] (> 97.5% by GC) was obtained from Acros Organics, bis(4-chlorophenyl)disulfide [1142-19-4] (98+%) referred here as **2-2** was purchased from TCI and bis(2-nitrophenyl)disulfide [1155-00-6] (99%) referred here as **1-1** was purchased from Sigma Aldrich.

The grinding jars were made inhouse from 316 stainless steel grade. All the ball bearings were sourced from Dejay Distribution Ltd.

1.1 Equipment

1.1.1 Ball mill grinder

The ball-mill grinding experiments were all performed using a Retsch MM400 Shaker Mill with the safety cover removed as the grinder motor vents to the front of the equipment warming the grinding jars on prolonged grinding. An external safety shield was used for safety.

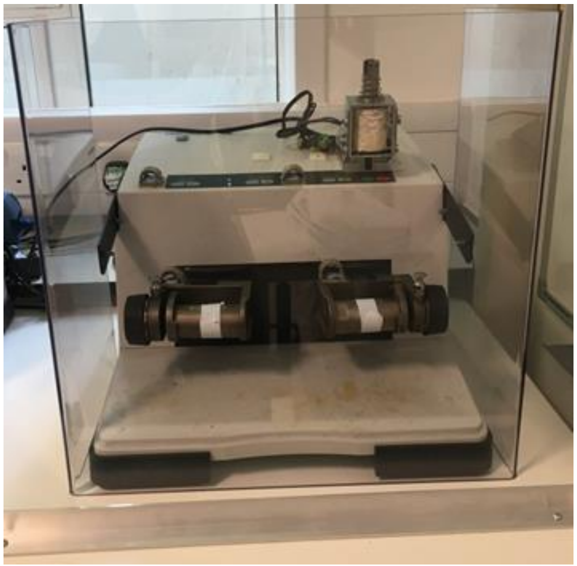

Figure S1: Modified Retsch MM400 Shaker Mill (ball mill grinder) with a solenoid installed to press the **START** button and with the manufacturer's safety cover removed and replaced by an external safety shield.

1.1.2 Automated solenoid to press the Retsch MM400 Shaker Mill start button

The Retsch MM400 Shaker Mill stops automatically once the run time has elapsed. It can only be automatically run for a maximum of 99 minutes. As these studies required in many cases up to multi-hour grinding, an automated solenoid (see Figure S 1 and Figure S 2) was manufactured in-house and controlled with in-house software to press repeatedly the **START** button as often as the experiment required it. To prevent the Retsch MM400 motor from overheating, 5–10 min rest was allowed for each hour grinding.

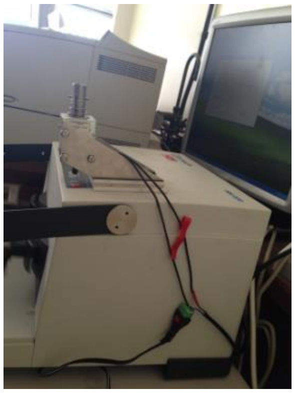

Figure S2: Solenoid used as push-finger to automatically press the **START** button on the Retsch MM400

1.1.3 Grinding jars

The snap closure grinding jars were manufactured in-house from hardened 316 stainless steel with 14.5 mL internal volume (19 mm ID × 54 mm internal length). These snap-closure grinding jars were used in previous studies.[1-2]

a) b) c)

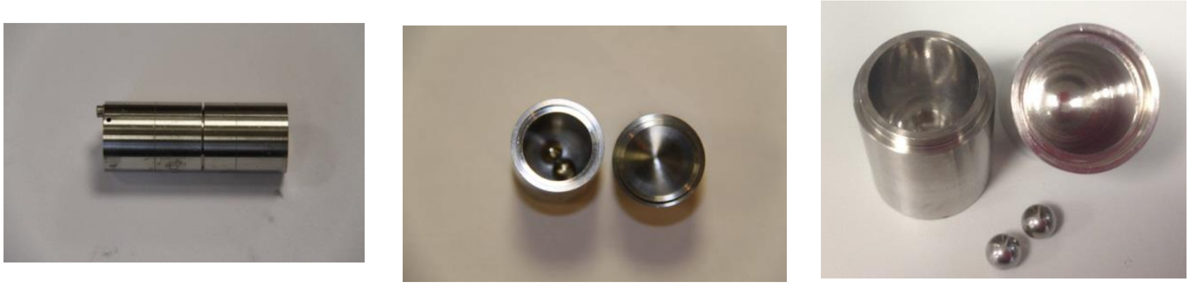

Figure S3 14.5 mL Stainless steel snap grinding jars; a) closed: b & c) open with two 7 mm ID hardened stainless steel ball bearings (each 1.37g). b) inside and c) outside the open grinding jar.

1.2 Balance

A Mettler Toledo XPE 205 Delta Range with an accuracy of 0.01mg was used for weighing the starting materials with a grease-proof weighing paper.

1.3 Automatic pipettes

A Sartorius eLINE Picus Electronic Pipette, 1-channel, 5–120 μL, in combination with Sartorius SafetySpaceTM, low retention, 2–120 μL, Sterile Filter Tip was used to achieve maximum accuracy allowing an accurate increment of 0.1 μL to be pipetted. The systematic error disclosed by the manufacturers in the pipette specification is: for 120 μL is ± 0.48 μL, for 60 μL is ± 0.36 μL and for 12 μL is ± 0.24 μL.

The Picus pipette was used in normal pipetting mode and primed repeatedly until the liquid was securely held by the pipette tip before dispensing 50 μL MeCN.[3]

For dispensing 2 μL dbu, a 0.5–10 μL Proline Plus Mechanical Pipette with TipOne XL Graduated Filter tip, max volume 20 μL from Starlab was used.

1.4 HPLC equipment

HPLC analysis of the chemical composition of the powders were performed using a modular Agilent 1200 Series HPLC system composed of a HPLC high pressure binary pump, an autosampler with injector programming capabilities, a Peltier-type column oven with 6 µL heat exchanger and a Diode Array Detector with a semi-micro flow cell (1.6 µL, 6 mm path length) to reduce peak dispersion when using short columns as in these cases. The flow-path was connected using 0.12 mm ID stainless steel tubing to minimize peak dispersion. For the analysis, an HPLC method described in Section 4.1 was used.

1.5 PXRD equipment

X-Ray powder diffractograms in the 2θ range 4–45° (CuKα radiation, step size 0.03°, time/step 100 s, 0.04 rad soller, VxA 40x40) were collected on an X-Pert PRO MPD powder X-ray diffractometer equipped with an X'Celerator detector. The PXRD equipment was available at the Department of Chemistry, University of Cambridge.

2 Experimental section: general concepts

2.1 Dynamic covalent chemistry

Dynamic combinatorial chemistry (DCC) is a method for the generation of new molecules formed by reversible reaction of simple building blocks under thermodynamic control; in a dynamic combinatorial library (DCL) all constituents are in equilibrium under the particular conditions of the experiment.[4] Typically, DCC is performed in solution using reversible chemistry, such as disulfide metathesis[5] and imine exchange.[6-7] DCC has the great advantage of proof reading and self-repair resulting in the most stable and reproducible composition at equilibrium. Furthermore, DCC is very sensitive to changes in the experimental conditions and adapts the composition of the product at the equilibrium accordingly. This makes dynamic covalent libraries (DCLs) powerful tools in identifying thermodynamic minima in a number of different contexts.[8]

2.2 Solid state dynamic covalent chemistry

Solid-state dynamic covalent chemistry (solid-state DCC) is an innovative, clean and highly atom efficient approach to the synthesis of molecules such as that discussed in this manuscript. For the reasons listed in Section 2.1, DCC can offer valuable tools to study equilibria under ball-mill grinding conditions. This one-pot reaction is achieved by the improved stability of the crystal structures of the product as compared to that of its starting materials in a reversible reaction governed by thermodynamic control under the given experimental conditions.[1] In contrast one-pot synthesis in traditional solution DCC requires the stabilizing effect of a host–guest complex. In the absence of a suitable guest, a statistical mixture of starting material and products can be expected, assuming all components have similar thermodynamic stability. As reported in our "proof-of-concept" paper[1] based on disulfide exchange chemistry, different polymorphs of the product of **1-2** can be directly synthesized from starting materials by solvent-free DCC, if the grinding is performed in the presence of substochiometric amounts of solvent, known as liquid-assisted grinding (LAG) or by ball mill neat grinding (NT).[1-2, 9.]

3 Experimental section: methodology

3.1 *Preparation of single point experiments for kinetic studies of the solid-state DCC reaction of 1-1 and 2-2 under NG and LAG*

This solid-state DCC reaction was prepared at a 200 mg scale by grinding 0.34 mmol of (2-NO_2PhS)$_2$ crystals (104.82 mg) and 0.34 mmol of (4-ClPhS)$_2$ crystals (97.66 mg) in a 14.5 mL stainless steel snap close grinding jar (see Section 1.1.3). Two 7 mm ID stainless-steel ball bearings were added and 2 μL dbu were pipetted on top of a ball bearing. Nothing else was added to prepare the kinetic curve for neat grinding. MeCN (50 μL) was added to the powder to prepare the kinetic curve for LAG. The grinding jars were closed and secured with tape as shown in Figure S1, and the solid was milled for the required time in a Retsch MM400 Shaker Mill.

3.1.1 Physical appearance of the product of ball mill grinding reaction

Typically, LAG experiments at high frequency (≥ 20 Hz) results in a slightly wet powder loosely covering the internal walls of the jar, the ball bearing being coated with very little powder (Figure S4a and S4b). Neat grinding at high frequency (≥ 20Hz), results in a dry powder loosely covering the internal walls of the jar, and little powder is attached to the ball bearings (Figure S4c and S4d). These experiments have good chemical and phase composition homogeneity of the powder in the jar. Neat grinding at 15 Hz has a long delay (around 100 min) before the reaction product **Form A** is being formed but the powder shows good homogeneity (Figure S4e and Figure S4f and Section 6.5). In contrast, LAG experiments at 15 Hz result in products forming a hard coat around the balls and at the bottom of the jar (Figure S4g and S4h). This results in very poor homogeneity of the powder (see Section 5.7) until it starts forming **Form B** in quantitative yield.

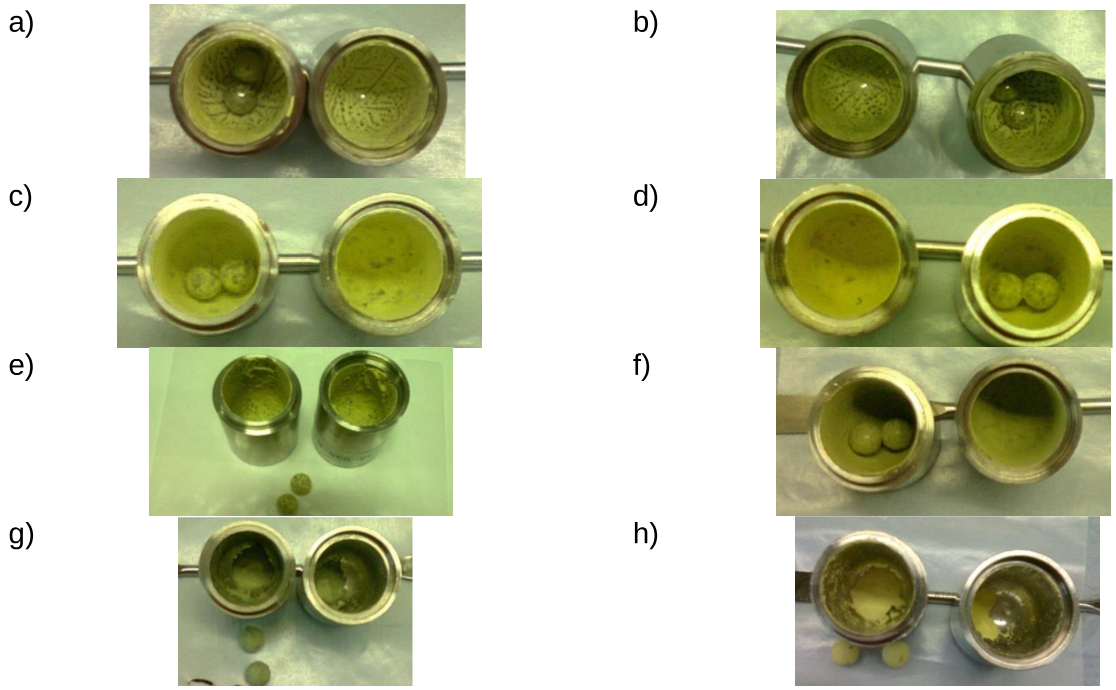

Figure S4 Physical appearance of the product of ball mill grinding: a) & b) LAG conditions grind ≥ 20Hz; c) & d) NG conditions grind ≥ 20Hz; e) & f) NG conditions grind at 15Hz and g) & h) LAG conditions grind at 15Hz.

4 Experimental section: analysis of the solid-state samples

4.1 Analysis by HPLC

The chemical composition of the DCC experiments performed by grinding, were analyzed by reversed phase HPLC using an Agilent 1200 Series composed of a HPLC high pressure binary pump, an autosampler, a column oven and a Diode Array Detector. As HPLC column a 1.8 μm Zorbax SB C18, (4.6 mm ID × 50 mm length) was used applying the following conditions:

Solvent A: water + 0.1% formic acid;

Solvent B: acetonitrile + 0.1% formic acid;

Gradient of 0–2 min 75–85% B with re-equilibration time of 1 min.

Flow rate: 2 mL/min; column temperature of 60 °C.

Injection volume: 1 μL.

The signal was monitored at 259 nm (8 nm bandwidth) with reference at 550 nm (100 nm bandwidth).

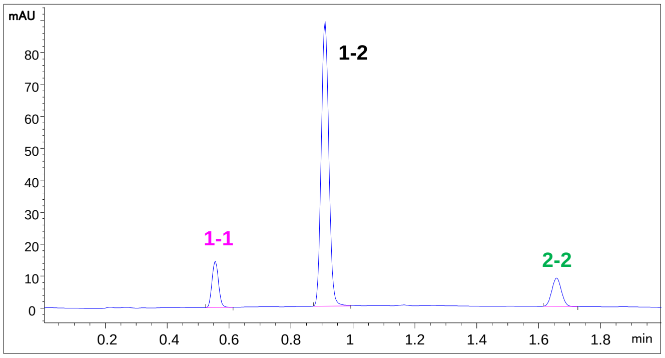

Figure S5 Typical HPLC chromatogram of enriched heterodimer **1-2** (made up of **Form A** + **Form B**). HPLC run contains the two homodimers, **1-1** and **2-2**.

4.1.1 Preparation of HPLC samples from solid-state DCC studies

HPLC samples were freshly prepared at a concentration of 1.0 mg/mL in acetonitrile containing 0.2 vol % of trifluoroacetic acid (TFA). The acid was added to quench dbu in the base-catalyzed reaction (see Section 3.1). In this way, preventing the disulfide exchange reaction from taking place in solution which would have resulted in the scrambling of the dynamic covalent chemistry library forming a statistical mixture of **1-1**, **2-2** and **1-2**, in a 1:1:2 proportion before HPLC analysis. The HPLC samples were then sonicated for a few minutes to bring them fully into solution before injection into the HPLC.

4.2 Analysis by PXRD

X-Ray powder diffractograms in the 2θ range 4–45° (CuKα radiation, step size 0.03°, time/step 100 s, 0.04 rad soller, VxA 40x40) were collected on an X-Pert PRO MPD powder X-ray diffractometer equipped with an X'Celerator detector. The PXRD equipment was available at the Department of Chemistry, University of Cambridge.

4.2.1 Sample preparation for PXRD analysis

Upon completion of the grinding experiment, the grinding jar was immediately opened and the powder transferred to an agar mortar. The powder was gently ground with an agar pestle, and then transferred to the sample holder for Powder X-Ray diffractometry.

The PXRD scans of the starting materials **1-1** and **2-2,** unreacted **1-1** + **2-2** and the two polymorphs **Form A** and **Form B** are shown in Figure S6.

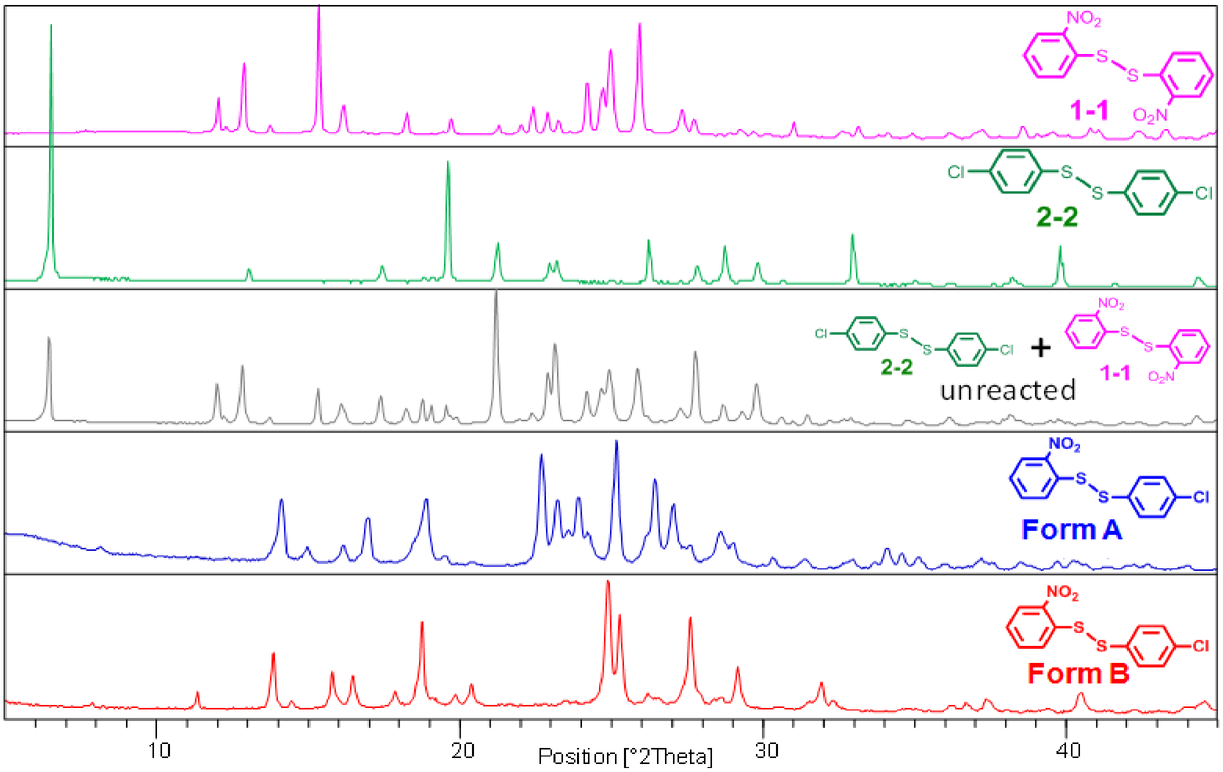

Figure S6 PXRD scan of the starting materials **1-1** and **2-2** and the two polymorph **Form A** and **Form B** using a X-Pert PRO MPD powder X-ray diffractometer, using Cu Kα radiation (0.154184 nm) at 40 kV, 40 mA with a scanning rate of 10°/min, and a 2θ angle ranging from 4 to 45°

4.3 Rietveld quantitative analysis of solid-state samples

The crystal structure of **Form A** and **Form B** are shown in Figure S7.

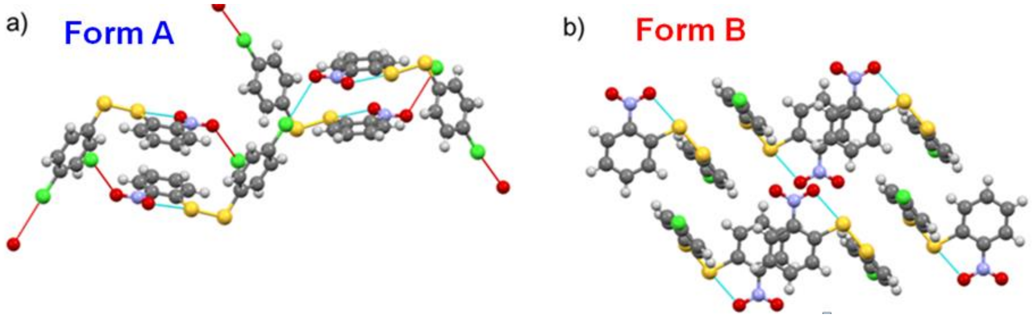

Figure S7 Arrangement of the 2 polymorph of 1-2 heterodimer molecule; **Form A** and **Form B** 1-D chains in (a) **Form A** [CCDC[1] code: FUQLIM01] and (b) **Form B** [CCDC code: FUQLIM] polymorphs respectively. Color codes: grey, carbon atoms; white, hydrogen atoms; yellow, sulfur atoms; red, oxygen atoms; blue, nitrogen atoms; green, chloride atoms.

4.3.1 Rietveld quantitative analysis

Rietveld refinements were performed with the software Topas V4.1.[10] For each of the four compounds the crystal structure retrieved from the Cambridge Structural Database was refined on a dataset collected on a pure single phase specimen: this first step was useful for the identification of the crystallographic planes most affected by preferred orientation phenomena. These were (0 1 0) for **1-1**; (0 0 1) for **2-2**; (1 0 2) and (0 0 1) for **Form A**; (0 1 0) for **Form B**. The March-Dollase model for preferred orientation was applied on these crystal planes in the quantitative analysis. No structural parameter was refined in the quantitative Rietveld refinements. The amorphous fractions were assumed to be negligible. **1-1** and **2-2** scale factors were constrained to be refined together (in order to have a **1-1**/**2-2** molar ratio of 1). This was found to be the most effective approach, after several trials and comparisons with the HPLC data [2]: even if these assumptions may sound rather strong (the **1-1** amorphous fraction may be in principle different from the **2-2** amorphous fraction, leading to a ratio of the crystalline fractions different from 1), the excellent agreement with the HPLC data validates our approach [2]. Furthermore, the peak shape and the parameters describing the diffractometer geometry were optimized using a LaB_6 NIST standard: only a Lorentzian Scherrer term (CS_L) for each phase was modeled in the Pseudo-Voigt functions for the quantitative analysis, the other parameters being fixed. A minimum limit of 30 nm for the crystal size was defined to avoid correlations with the background. On the basis of tests performed on six replicates made on the same sample, we estimate an accuracy error of ± 6 mol % absolute (see the following results from six replicates). A shifted Chebyshev function with seven parameters was used to fit the background. *Rwp* and $\chi 2$ values ranged typically from 10% to 15% and from 3 to 6, respectively. Figure S 8 shows a representative Rietveld refinement plot.

Rietveld quantitative results on six replicates from the same sample (with relative estimated standard deviations), using a minumum limit of 30 nm for the Sherrer term CS_L:

	**1-1** [%M(e.s.d)]	**2-2** [%M(e.s.d)]	**Form A** [%M(e.s.d)]	**Form B** [%M(e.s.d)]
replicate 1	28.6(5)	23.1(4)	24.3(6)	24.1(6)
replicate 2	27.8(4)	22.4(4)	23.4(5)	26.4(5)
replicate 3	27.3(4)	22.0(4)	22.7(5)	28.0(5)
replicate 4	27.3(4)	22.0(4)	22.1(5)	28.7(6)
replicate 5	27.2(4)	22.0(4)	21.0(6)	29.8(6)
replicate 6	27.0(4)	21.8(6)	20.7(5)	30.6(5)

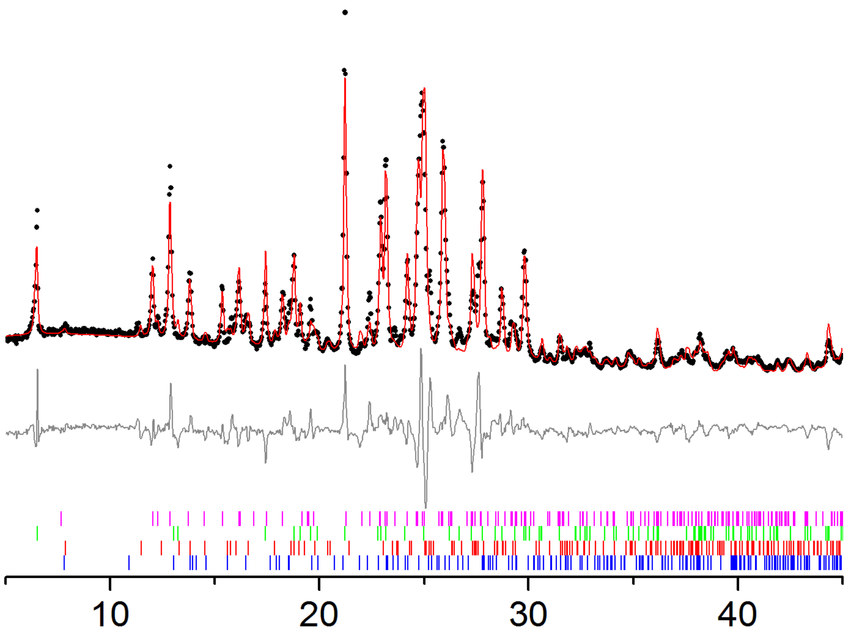

Figure S8 Experimental (black dots), calculated (orange line) and difference (grey line) patterns for a sample containing 40.0(9) wt%, 32.3(8) wt%, 15(1) wt%, 13(1) wt% of 1-1, 2-2, Form B and Form A respectively; x axis is in degrees of 2θ. Peak positions are marked in pink, green, red and blue for 1-1, 2-2, Form B and Form A respectively. The χ2 and Rwp are 3.3 and 11.6% respectively.

4.3.2 Crystal size determination from powder diffraction data

The crystal size was estimated by whole pattern Rietveld refinements which incorporate the Scherrer equation,

$$\text{crystal size [in nm]} = k\lambda/(10 \cdot \Delta\theta \cdot \cos\theta),$$

where *k* is a crystal shape factor most often assumed to be 0.9, λ is the radiation wavelength and θ is the diffraction angle, into the peak shape function in a given crystallographic direction (hkl) according to the following equation:

$$\text{fwhm}(2\theta, hkl) = (180/\pi)\ \lambda\ /\ (\cos\theta \cdot \text{crystal size}),$$

where fwhm(2θ) is the full width at half maximum of the peak at a given diffraction angle and a given crystallographic direction.[11]

The LaB_6 660b NIST standard[12] was used to model the instrumental contribution to peak broadening using a fundamental parameters approach with the software Topas v4.1.[13] Because of the nanocrystalline nature of the analysed powders and the absence of peaks above 45º in

2θ, the sample contribution to the peak broadening was assumed to be related to size only. A Lorentzian peak shape was found to fit better than a Gaussian one. In order to minimize the correlations and the e.s.d., the crystal size contribution to peak broadening was modelled as isotropic, i.e., using one single parameter for all crystallographic directions. We here remind that the e.s.d. from the Rietveld calculation has no bearing on the precision or accuracy, being merely related to the mathematical fit of the model. For what concerns the accuracy of the size determination, it is known that for a typical laboratory X-ray diffraction instrument the Scherrer analysis provides sensitivity to crystallite size in the 1–100 nm range, the upper limit being set by the instrumental broadening. This also means that the smaller the crystal size, the less the Scherrer size value is affected by how the instrumental broadening is defined.[11] In our experience related to the present case, a Scherrer crystal size of 100nm can vary by up to 30% relative of its value depending on the way the fundamental parameters are used to fit the LaB_6 660b NIST standard - e.g., depending on whether the size and microstrain contribution for the NIST standard are assumed to be zero or are allowed to give a contribution to the LaB_6 peak shape. However, if the crystal size is 60 nm, this variation is no more than 10% relative. In other words, the smaller the crystal size, the more reliable the number is. It is also important to point out that the peak shape tends to be dominated by the larger crystallites rather than the smaller ones, so the calculated size tends to be overestimated.[11]

5 Experimental section: kinetic studies

We present here the data for the kinetic studies for LAG and NG experiments for the solid-state DCC reaction starting from equimolar amounts of **1-1** and **2-2** in the presence of dbu (base catalyst) forming **1-2** as **Form A** under NG and as **Form B** under LAG conditions.

5.1 Tabulation of the locations of the data used for kinetic studies

Table S 1 Location of the tables and figures for the LAG and NG kinetic studies

Liquid assisted grinding: kinetic studies (Section 5)						
Frequency used for the kinetic study	Chemical composition (HPLC analysis)	Solid state composition (Rietveld refinement from PXRD scans)	PXRD scans	Kinetic curve for chemical composition & solid state composition versus grinding time	Scherrer size tabulation	Scherrer size plot with respect to position in kinetic curve
**30 Hz**	Table S 2	Table S 3	Figure S 9	Figure S10	Table S 4	Figure S11
**25 Hz**	Table S 5	Table S 6	Figure S12	Figure S13	Table S 7	Figure S14
**20 Hz**	Table S 8	Table S 9	Figure S15	Figure S16	Table S10	Figure S17
**15 Hz**	Table S11	Table S12	Figure S18	Figure S19	Table S13	Figure S20
**Neat grinding: kinetic studies (Section 6)**						
Frequency used for the kinetic study	Chemical composition (HPLC analysis)	Solid state composition (Rietveld refinement from PXRD scans)	PXRD scans	Kinetic curve for chemical composition & solid state composition versus grinding time	Scherrer size tabulation	Scherrer size plot with respect to position in kinetic curve
**30 Hz**	Table S15	Table S16	Figure S25	Figure S26	Table S17	Figure S27
**25 Hz**	Table S18	Table S19	Figure S28	Figure S29	Table S20	Figure S30
**20 Hz**	Table S21	Table S22	Figure S31	Figure S32	Table S23	Figure S33
**15 Hz**	Table S24	Table S25	Figure S34	Figure S35	Table S26	Figure S36

5.2 Kinetic studies of ball mill LAG of 1-1 and 2-2 with 2 mol % dbu

Ball mill LAG (50 μL MeCN) reactions were performed starting from equimolar mixtures of **1-1** and **2-2** with 2 mol % dbu as a base catalyst.

5.3 Kinetics LAG studies of 1-1 and 2-2 with 2 mol % dbu at 30 Hz

This section presents the characterization of the reaction product of the kinetic ball mill LAG studies at 30 Hz using single point experiments. The solid-state DCC reaction was performed following the procedure described in Section 3.1. This reaction was run at different grinding times ranging from 5 min up to 24 h to investigate the kinetic profile leading to thermodynamic equilibrium. The chemical composition of the reaction product was determined by HPLC (Table S 2 and Figure S10a). The phase composition of the reaction product was calculated from the Rietveld refinement of the PXRD scans (Table S 3 and Figure S10b). The crystal size of **1-1**, **2-2** and **Form B** in the product was calculated using the Scherrer equation applied to the Rietveld refinement (Table S 4 and Figure S11). The PXRD scans are displayed on Figure S 9.

Table S 2 Experimentals details of single point grinding experiments of the solid state DCC reaction of **1-1** and **2-2** performed in the presence of 2%M dbu under LAG conditions at 30 Hz. Experiments are run from 5 min up to 24 h. The chemical composition of reaction products is determined by HPLC analysis. The HPLC conditions are shown below. This data is plotted on Figure S10a.

Kinetics of ball mill **LAG** reaction @**30Hz**					
[**1-1**]+[**2-2**]+2%M dbu + 50μL MeCN					
Reagents:	**$(2NO_2PhS)_2$**	**$(4ClPhS)_2$**	**HPLC conditions** [λ=259(8nm) ref 550(100nm)]		
MW:	308.33	287.23	Zorbax SB C18, 1.8 μm; 4.6mm ID x50 mm		
%M:	50%M	50%M	**A**: H_2O+0.1% HCOOH; **B**: MeCN+0.1%HCOOH		
mmol:	0.34 mmol	0.34 mmol	0-2 min 75-85%B; 2ml/min; 60°C; run time=2.0 min		
mgs:	104.38 mg	97.63 mg	**HPLC results**		
grinding time @ 30 Hz	$(2NO_2PhS)_2$ weighed	$(4ClPhS)_2$ weighed	$(2NO_2PhS)_2$ (**1-1**)	Product (**1-2**)	$(4ClPhS)_2$ (**2-2**)
min	mg	mg	%M	%M	%M
0	Expected %M from weighing		50.0	0.0	50.0
**5**	104.87	97.70	49.2	**0.4**	50.0
**5**	104.89	97.64	50.4	**0.5**	49.1
**5**	104.84	97.69	50.2	**0.5**	49.4
**5**	104.78	97.62	49.6	**1.7**	48.8
**5**	104.83	97.68	49.8	**1.1**	49.1
**5**	104.84	97.62	51.8	**0.8**	47.3
**10**	104.87	97.68	48.0	**3.4**	48.6
**10**	104.80	96.65	47.4	**4.9**	47.7
**13**	104.83	97.60	41.9	**17.4**	40.7
**14**	104.86	97.68	27.7	**44.7**	27.6
**15**	104.82	97.67	15.6	**69.1**	15.3
**17**	104.76	97.63	8.1	**84.4**	7.4
**18**	104.83	97.64	7.8	**85.0**	7.3
**20**	104.86	97.57	2.2	**96.5**	1.3
**20**	104.77	97.63	2.7	**95.4**	1.9
**25**	104.82	97.60	2.1	**96.2**	1.6
**30**	104.84	97.66	2.2	**96.5**	1.3
**30**	104.80	97.65	1.8	**97.2**	0.9
**45**	104.86	97.65	1.8	**97.4**	0.8
**300**	104.87	97.70	1.9	**97.2**	1.5
**24h**	104.87	97.68	2.4	**95.7**	1.9

Table S 3 Solid state composition of the reaction products calculated using Rietveld refimenment from PXRD scans of single point experiments of the solid state DCC reaction of **1-1** and **2-2** performed in the presence of 2%M dbu under LAG conditions at 30 Hz. Experiments are run from 5 min up to 24 h. This data is plotted on Figure S 10b. The PXRD scans used for the Rietveld refinement are shown on Figure S 9

Kinetics of ball mill **LAG** reaction @**30Hz**<br>0.68 mmol DCC ([**1-1**]+[**2-2**] + 2%M dbu+50μL MeCN)														
**grinding time @ 30Hz**	**HPLC**			**PXRD: Rietveld refinement**										
	**1-1**	**2-2**	**1-2**	**1-1**		**2-2**		**Form A**		**Form B**		Rwp Rietveld refinement Goodness	chisq Rietveld refinement Fit Index
(min)	%M	%M	%M	%M	e.s.d. mol%	%M	e.s.d. mol%	%M	e.s.d. mol%	%M	e.s.d. mol%		
**0**	50.0	50.0	**0.0**	**50.0**		**50.0**		**0.0**		**0.0**			
**5**	49.8	49.1	**1.1**	**45.2**	2.0	**42.1**	1.9	**7.7**	2.9	**5.0**	2.2	*9.0*	*3.8*
**5**	51.8	47.3	**0.8**	**46.8**	1.3	**43.6**	1.2	**5.4**	1.9	**4.2**	1.5	*10.6*	*5.2*
**10**	48.0	48.6	**3.4**	**43.9**	2.2	**40.9**	2.1	**9.1**	3.3	**6.1**	2.7	*8.1*	*3.3*
**10**	47.4	47.7	**4.9**	**46.4**	1.2	**43.2**	1.1	**7.0**	1.8	**3.4**	0.9	*10.7*	*5.3*
**13**	41.9	40.7	**17.4**	**40.3**	1.1	**37.5**	1.1	**6.9**	1.9	**15.3**	1.0	*8.5*	*4.2*
**14**	27.7	27.6	**44.7**	**26.8**	0.4	**25.0**	0.4	**4.9**	0.9	**43.3**	0.6	*8.3*	*6.8*
**17**	8.1	7.4	**84.4**	**5.7**	0.7	**5.3**	0.6	**5.4**	1.7	**83.6**	1.7	*9.2*	*4.5*
**18**	7.8	7.3	**85.0**	**5.8**	0.6	**5.4**	0.6	**4.8**	1.5	**84.0**	1.5	*9.6*	*4.7*
**20**	2.2	1.3	**96.5**	**2.3**	2.4	**2.1**	2.2	**4.8**	2.9	**90.8**	4.0	*8.1*	*3.4*
**20**	2.7	1.9	**95.4**	**1.0**	0.7	**0.9**	0.6	**6.3**	1.4	**91.8**	1.6	*15.8*	*7.8*
**25**	2.1	1.6	**96.2**	**1.0**	0.9	**0.9**	0.8	**5.0**	1.4	**93.1**	1.8	*12.3*	*6.0*
**30**	2.2	1.3	**96.5**	**3.9**	2.6	**3.5**	2.4	**6.6**	2.9	**86.0**	4.1	*7.3*	*3.0*
**30**	1.8	0.9	**97.2**	**1.6**	0.9	**1.4**	0.8	**7.2**	1.3	**89.8**	1.6	*11.5*	*8.5*
**5h**	1.9	1.5	**97.2**	**0.2**	0.3	**0.2**	0.2	**2.6**	0.6	**97.0**	0.7	*13.5*	*11.0*
**24h**	2.4	1.9	**95.7**	**0.3**	0.3	**0.3**	0.3	**2.6**	0.5	**96.9**	0.6	*16.0*	*13.0*

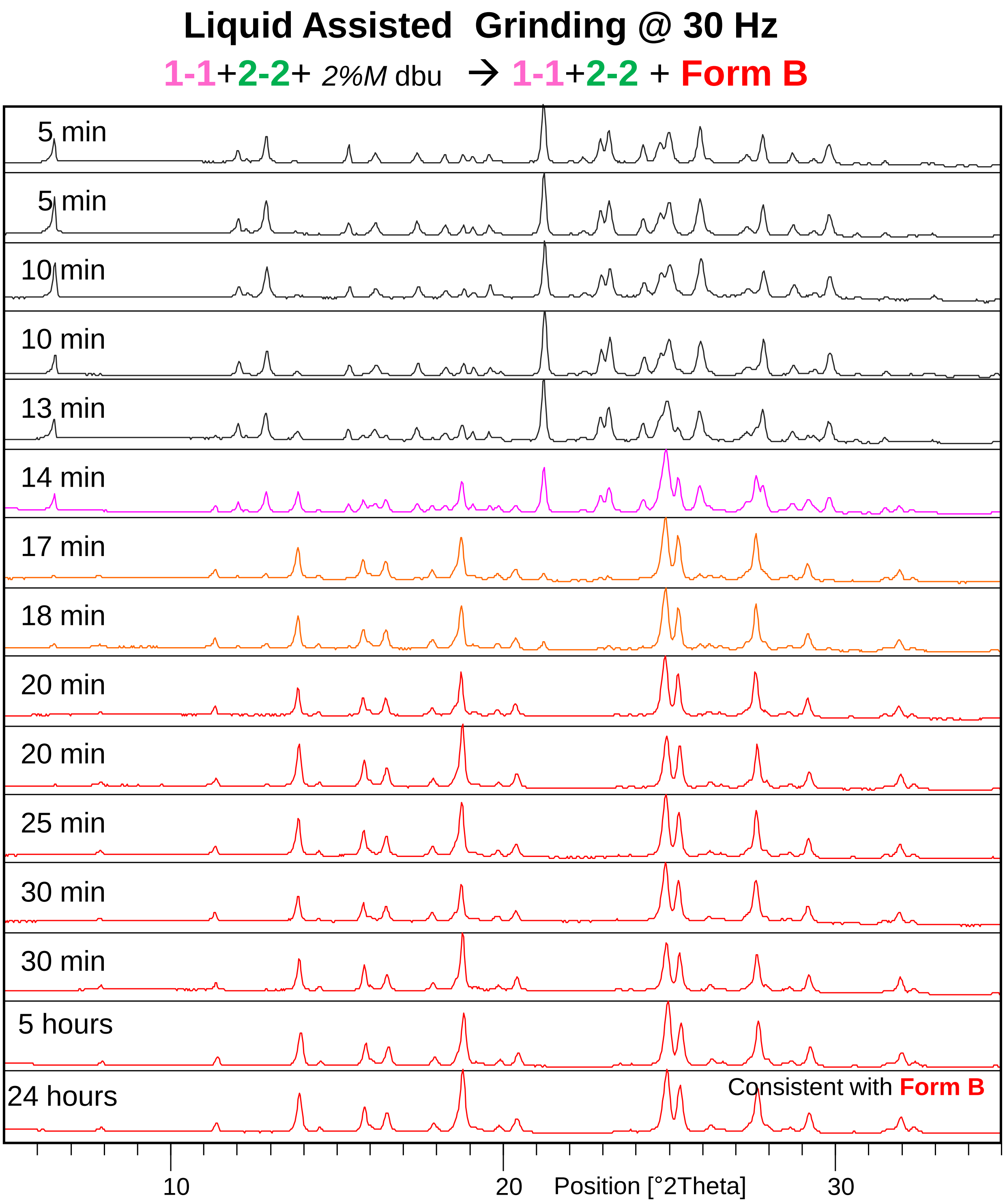

Figure S 9 PXRD scans of the reaction product of the kinetic study of a ball mill LAG reaction (50μL MeCN) at 30 Hz. Each PXRD scan was obtained from a single point experiment. The PXRD sample was prepared immediately after completion of the grinding experiment, and scanned between 5 and 45° (2θ). Typical PXRD scans are shown on Figure S 6

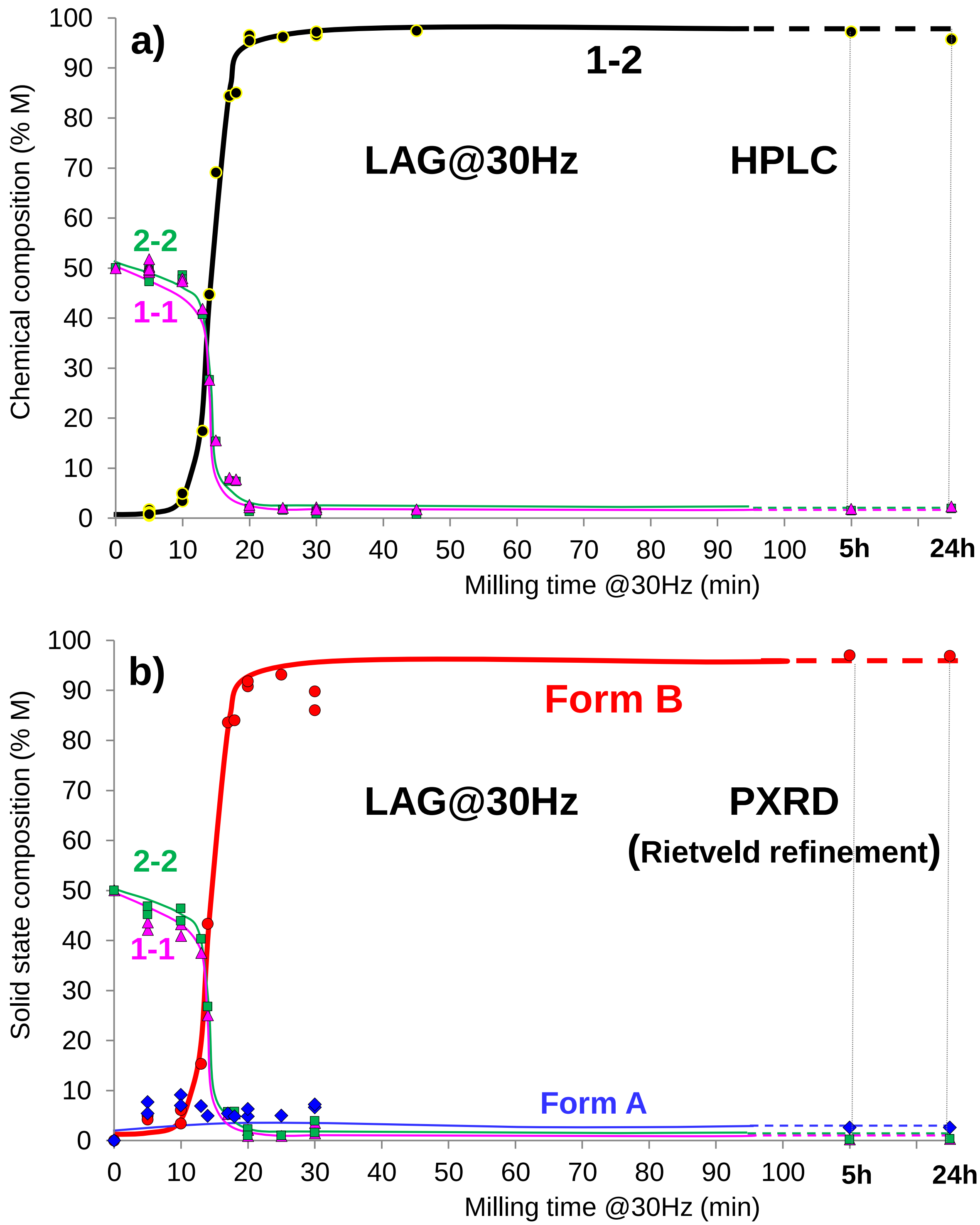

Figure S 10 Kinetic plots of the solid state DCC LAG reaction at 30 Hz of **1-1** and **2-2** in the presence of 2%M dbu leading to thermodynamic equilibrium and quantitative formation of **Form B**. a) chemical composition determined by HPLC; b) solid state composition calculated by the Rietveld refinement from PXRD data.

Table S 4 Crystal Scherrer size of the starting materials (**1-1** and **2-2**) and the product **Form B** in relation to the chemical and solid state composition of each single point experiment of the LAG reaction at 30Hz.

Kinetics of ball mill LAG reaction @30Hz															
0.68 mmol DCC ([1-1]+[2-2] + 2%M dbu +50µL MeCN															
**grinding time @ 30Hz**	**HPLC**			**Rietveld refinement**				**Scherrer size**							
	**1-1**	**2-2**	**1-2**	**1-1**	**2-2**	**Form A**	**Form B**	**1-1**		**2-2**		**Form A**		**Form B**	
min	%M	%M	%M	%M	%M	%M	%M	**nm**	e.s.d. nm	**nm**	e.s.d. nm	**nm**	e.s.d. nm	**nm**	e.s.d. nm
**5**	49.8	**49.1**	**1.1**	**45.2**	**42.1**	**7.7**	**5.0**	271	58	190	24				
**5**	51.8	**47.3**	**0.8**	**46.8**	**43.6**	**5.4**	**4.2**	155	14	173	14				
**10**	48.0	**48.6**	**3.4**	**43.9**	**40.9**	**9.1**	**6.1**	132	19	167	24				
**10**	47.4	**47.7**	**4.9**	**46.4**	**43.2**	**7.0**	**3.4**	132	11	163	13				
**13**	41.9	**40.7**	**17.4**	**40.3**	**37.5**	**6.9**	**15.3**	130	12	169	15				
**14**	27.7	**27.6**	**44.7**	**26.8**	**25.0**	**4.9**	**43.3**	166	12	190	13			**156**	9
**17**	8.1	**7.4**	**84.4**	**5.7**	**5.3**	**5.4**	**83.6**							**123**	7
**18**	7.8	**7.3**	**85.0**	**5.8**	**5.4**	**4.8**	**84.0**							**153**	10
**20**	2.2	**1.3**	**96.5**	**2.3**	**2.1**	**4.8**	**90.8**							**174**	17
**20**	2.7	**1.9**	**95.4**	**1.0**	**0.9**	**6.3**	**91.8**							**149**	8
**25**	2.1	**1.6**	**96.2**	**1.0**	**0.9**	**5.0**	**93.1**							**148**	8
**30**	2.2	**1.3**	**96.5**	**3.9**	**3.5**	**6.6**	**86.0**							**130**	11
**30**	1.8	**0.9**	**97.2**	**1.6**	**1.4**	**7.2**	**89.8**							**172**	11
**5h**	1.9	**1.5**	**97.2**	**0.2**	**0.2**	**2.6**	**97.0**							**120**	3
**24h**	2.4	**1.9**	**95.7**	**0.3**	**0.3**	**2.6**	**96.9**							**108**	2
Scherrer size calculation is only reliable for contents > 20%M, must be in range 30 nm-350 nm and the size > 3 x esd															

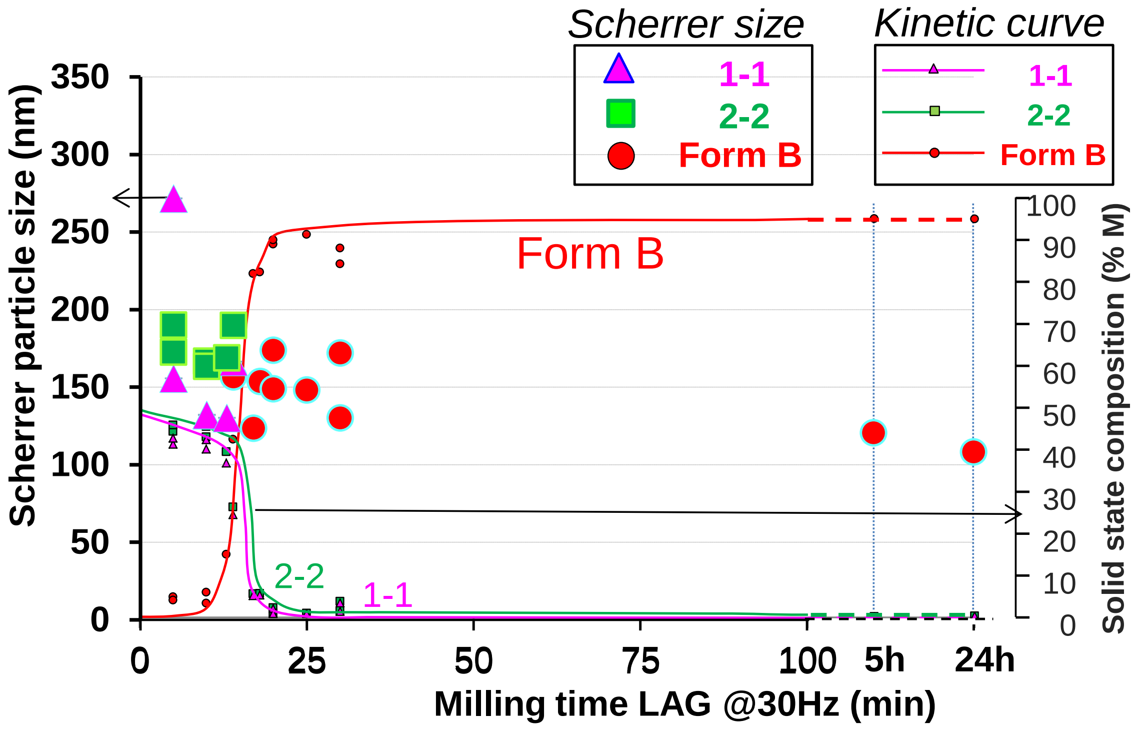

Figure S11 Scherrer size (nm) for the homodimers **1-1**, **2-2** and the reaction product **Form B** determined at different grinding times of the experimental kinetic stiudy at 30 Hz . **Form A** is not formed by LAG experiments. Scherrer size determination is only reliable for compounds > 20%M and their estimated standard deviations (esd) are much smaller than the symbols used. Y-axis on the right is expressed as %M composition of **1-1**, **2-2** and **Form B**.

5.4 *Kinetics LAG studies of 1-1 and 2-2 with 2 mol % dbu at 25 Hz*

This section presents the characterization of the reaction product of the kinetic ball mill LAG studies at 25 Hz using single point experiments. The solid-state DCC reaction was performed following the procedure described in Section 3.1. The solid-state DCC reaction was run at different grinding times ranging from 5 min up to 24 h to investigate the kinetic profile leading to thermodynamic equilibrium. The chemical composition of the reaction product was determined by HPLC (Table S 5 and Figure S13a). The phase composition of the reaction product was calculated from the Rietveld refinement of the PXRD scans (Table S 6 and Figure S13b). The crystal size of **1-1**, **2-2** and **Form B** in the product was calculated using the Scherrer calculation applied to the Rietveld refinement (Table S 7 and Figure S14). The PXRD scans are displayed on Figure S12.

Table S 5 Experimentals details of single point grinding experiments of the solid state DCC reaction of **1-1** and **2-2** in the presence of 2%M **dbu** performed under LAG conditions at 25 Hz. Experiments are run from 5 min up to 24 h. The chemical composition of reaction products is determined by HPLC analysis. The HPLC conditions are shown below. This data is plotted on Figure S13a.

Kinetics of ball mill **LAG** reaction @**25Hz**					
[**1-1**]+[**2-2**]+2%M dbu + 50μL MeCN					
Reagents:	**$(2NO_2PhS)_2$**	**$(4ClPhS)_2$**	**HPLC conditions** [λ=259(8nm) ref 550(100nm)]		
MW:	308.33	287.23	Zorbax SB C18, 1.8 μm; 4.6mm ID x50 mm		
%M:	50%M	50%M	**A**: H_2O+0.1% HCOOH; **B**: MeCN+0.1%HCOOH		
mmol:	0.34 mmol	0.34 mmol	0-2 min 75-85%B; 2ml/min; 60°C; run time=2.0 min		
mgs:	104.38 mg	97.63 mg	**HPLC results**		
grinding time @ 25 Hz	$(2NO_2PhS)_2$ weighed	$(4ClPhS)_2$ weighed	$(2NO_2PhS)_2$ (**1-1**)	Product (**1-2**)	$(4ClPhS)_2$ (**2-2**)
min	mg	mg	%M	%M	%M
**0**	Expected %M from weighing		**50.0**	**0.0**	**50.0**
**10**	104.80	97.70	45.8	**8.0**	46.3
**15**	104.79	97.69	41.9	**15.4**	42.7
**20**	104.76	97.65	48.8	**0.6**	49.6
**22**	104.79	97.69	43.8	**7.5**	48.7
**25**	104.79	97.68	31.7	**36.6**	31.7
**27**	104.83	97.65	2.1	**96.2**	1.7
**30**	104.80	97.64	1.6	**97.1**	1.3
**40**	104.79	97.68	2.0	**96.7**	1.4
**45**	104.80	97.67	2.3	**95.9**	1.8
**60**	104.80	97.68	1.9	**96.5**	1.6
**90**	104.83	97.64	2.0	**96.7**	1.4
**300**	104.80	97.72	2.9	**95.1**	2.1
**24h**	104.87	97.66	2.5	**96.0**	1.6

Table S 6 Solid state composition of the reaction products calculated using Rietveld refimenment from PXRD scans of single point experiments of the solid state DCC reaction of **1-1** and **2-2** performed in the presence of 2%M **dbu** under LAG conditions at 25 Hz. Experiments are run from 5 min up to 24 h. This data is plotted on Figure S13b. The PXRD scans used for the Rietveld refinement are shown on Figure S12.

Kinetics of ball mill **LAG** reaction @**25Hz**

0.68 mmol DCC ([**1-1**]+[**2-2**] + 2%M dbu+50μL MeCN)

**grinding time @ 25Hz**	**HPLC**			**PXRD: Rietveld refinement**									
	**1-1**	**2-2**	**1-2**	**1-1**		**2-2**		**Form A**		**Form B**		Rwp Rietveld refinement Goodness	chisq Rietveld refinement Fit Index
(min)	%M	%M	%M	%M	e.s.d. mol%	%M	e.s.d. mol%	%M	e.s.d. mol%	%M	e.s.d. mol%		
**0**	**50.0**	**50.0**	**0.0**	**50.0**		**50.0**		**0.0**		**0.0**			
**10**	45.8	46.3	**8.0**	**42.2**	0.7	**39.4**	0.6	**6.9**	1.0	**11.5**	0.8	*12.3*	*3.1*
**15**	41.9	42.7	**15.4**	**42.5**	0.7	**39.5**	0.7	**4.9**	1.0	**13.1**	0.7	*8.5*	*2.1*
**20**	48.8	49.6	**0.6**	**44.0**	0.7	**41.0**	0.6	**8.2**	1.0	**6.8**	0.7	*14.5*	*3.6*
**22**	2.1	1.7	**96.2**	**45.3**	0.7	**42.2**	0.7	**6.8**	1.0	**5.7**	0.6	*13.1*	*2.3*
**25**	31.7	31.7	**36.6**	**32.8**	0.6	**30.6**	0.6	**5.4**	1.1	**31.3**	0.7	*8.7*	*2.3*
**27**	43.8	48.7	**7.5**	**2.5**	0.8	**2.3**	0.8	**5.0**	0.8	**90.2**	1.3	*20.2*	*3.5*
**30**	1.6	1.3	**97.1**	**3.5**	0.7	**3.3**	0.7	**4.5**	0.9	**88.7**	1.2	*12.3*	*2.2*
**40**	2.0	1.4	**96.7**	**1.2**	0.8	**1.2**	0.8	**6.5**	0.8	**91.1**	1.3	*4.3*	*17.4*
**45**	2.3	1.8	**95.9**	**3.0**	0.9	**2.9**	0.8	**4.9**	0.9	**89.2**	1.4	*13.4*	*2.4*
**60**	1.9	1.6	**96.5**	**2.5**	0.8	**2.3**	0.7	**3.7**	0.8	**91.5**	1.3	*12.7*	*2.2*
**90**	2.0	1.4	**96.7**	**2.1**	0.8	**1.9**	0.7	**4.9**	0.8	**91.2**	1.3	*17.0*	*2.9*
**300**	2.9	2.1	**95.1**	**2.8**	0.7	**2.5**	0.7	**4.4**	0.7	**90.3**	1.1	*16.8*	*3.0*
**24h**	2.5	1.6	**96.0**	**1.5**	0.8	**1.4**	0.8	**7.0**	0.9	**90.1**	1.4	*17.5*	*3.1*

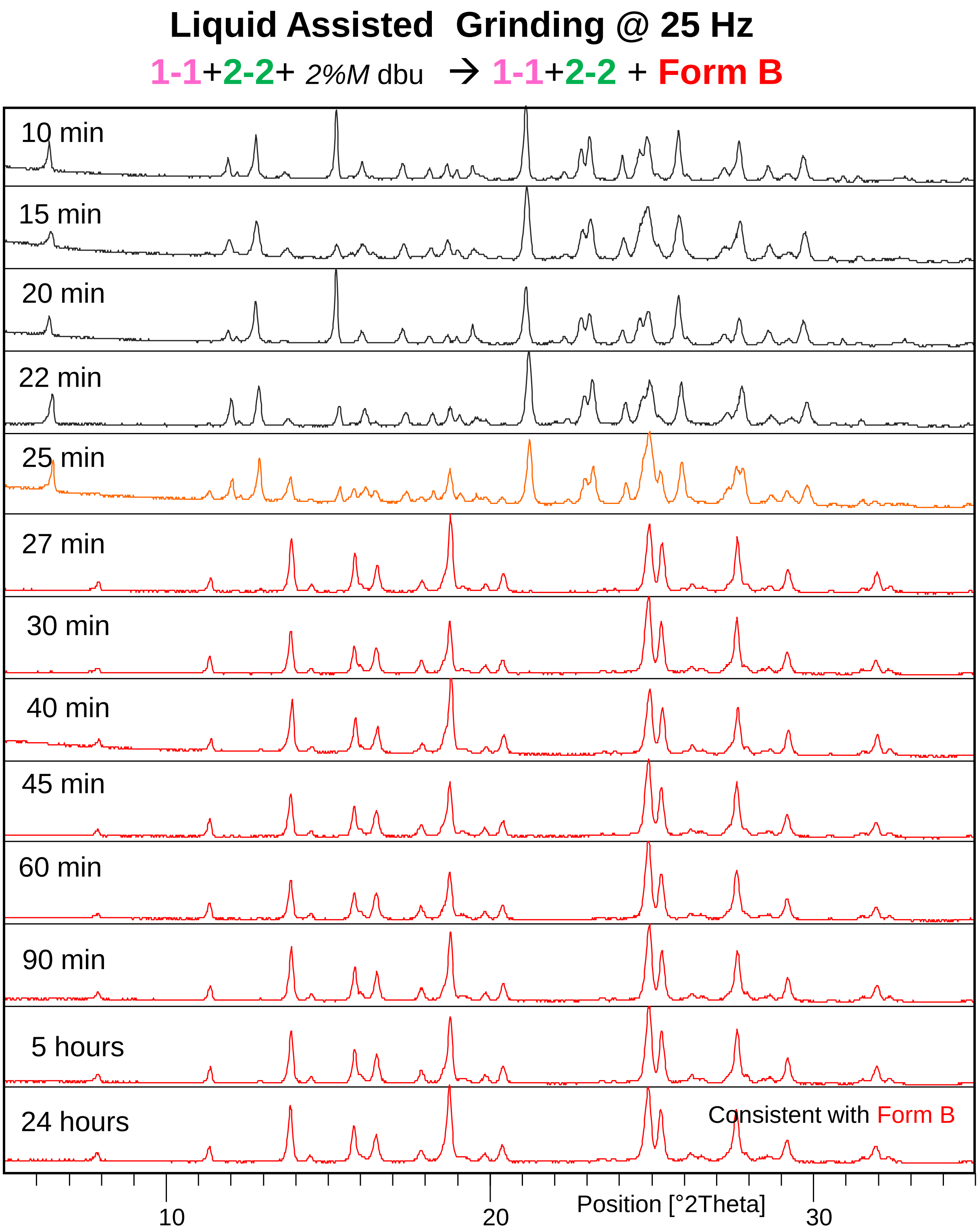

Figure S 12 PXRD scans of the reaction product of the kinetic study of a ball mill LAG reaction (50μL MeCN) at 25 Hz. Each PXRD scan was obtained from a single point experiment. The PXRD sample was prepared immediately after completion of the grinding experiment, and scanned between 5 and 45° (2θ). Typical PXRD scans are shown on Figure S 6.

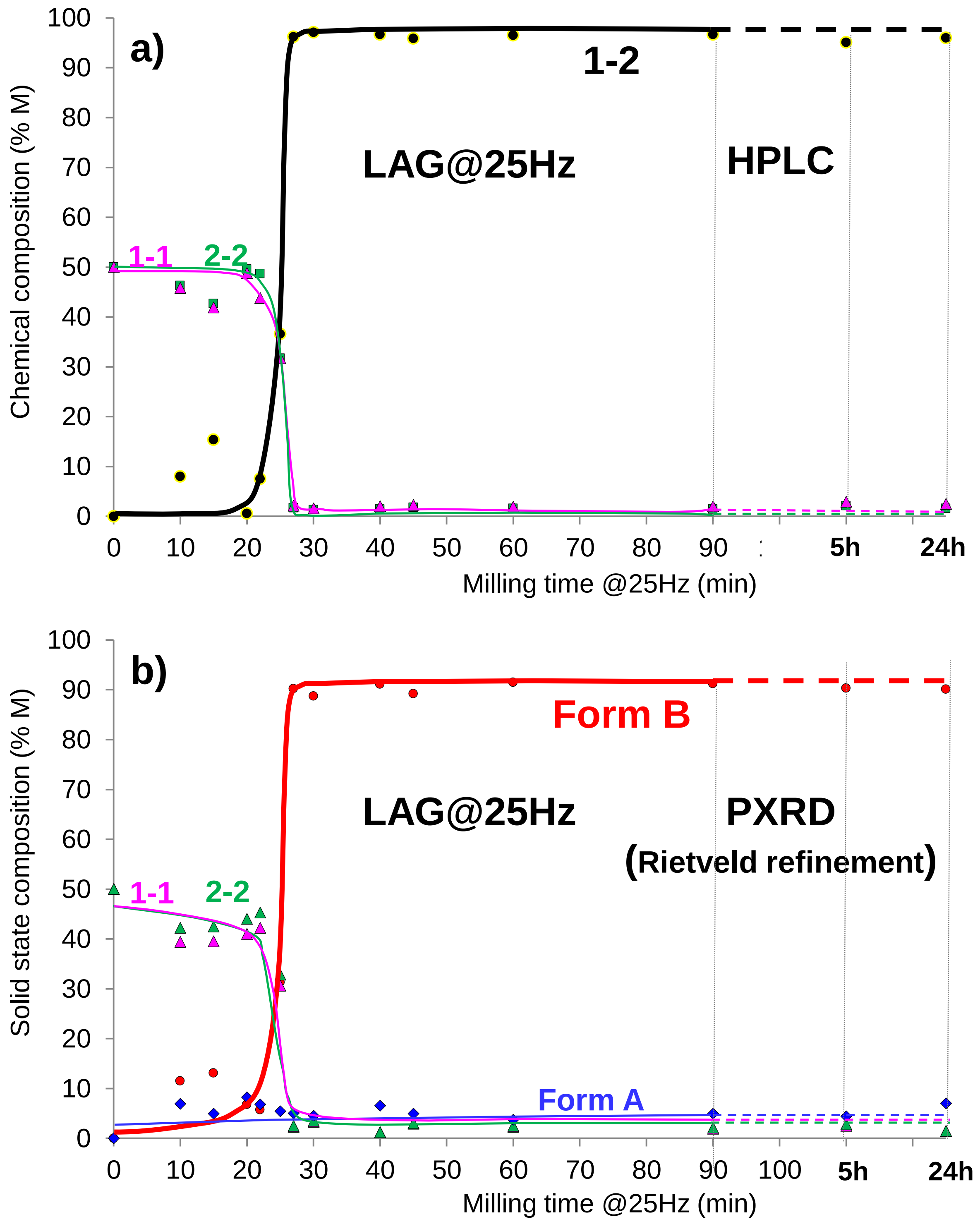

Figure S 13: Kinetic plots of the solid state DCC LAG reaction at 25 Hz of **1-1** and **2-2** in the presence of 2%M **dbu** leading to thermodynamic equilibrium and quantitative formation of **Form B**. a) chemical composition determined by HPLC; b) solid state composition calculated by the Rietveld refinement from PXRD data.

Table S 7 Crystal Scherrer size of the starting materials (**1-1** and **2-2**) and the product **Form B** in relation to the chemical and solid state composition of each single point experiment of the LAG reaction at 25Hz.

Kinetics of ball mill LAG reaction @**25Hz**															
0.68 mmol DCC ([1-1]+[2-2] + 2%M dbu +50µL MeCN															
**grinding time @ 25Hz**	**HPLC**			**Rietveld refinement**				**Scherrer size**							
	**1-1**	**2-2**	**1-2**	**1-1**	**2-2**	**Form A**	**Form B**	**1-1**		**2-2**		**Form A**		**Form B**	
min	%M	%M	%M	%M	%M	%M	%M	**nm**	e.s.d. nm	**nm**	e.s.d. nm	**nm**	e.s.d. nm	**nm**	e.s.d. nm
**10**	45.8	**46.3**	**8.0**	**42.2**	**39.4**	**6.9**	**11.5**			137	6				
**15**	41.9	**42.7**	**15.4**	**42.5**	**39.5**	**4.9**	**13.1**	95	5	94	4				
**20**	48.8	**49.6**	**0.6**	**44.0**	**41.0**	**8.2**	**6.8**			99	3				
**22**	2.1	**1.7**	**96.2**	**45.3**	**42.2**	**6.8**	**5.7**	149	8	84	3				
**25**	31.7	**31.7**	**36.6**	**32.8**	**30.6**	**5.4**	**31.3**	147	11	91	4			118	9
**27**	43.8	**48.7**	**7.5**	**2.5**	**2.3**	**5.0**	**90.2**							137	4
**30**	1.6	**1.3**	**97.1**	**3.5**	**3.3**	**4.5**	**88.7**							137	4
**40**	2.0	**1.4**	**96.7**	**1.2**	**1.2**	**6.5**	**91.1**							139	5
**45**	2.3	**1.8**	**95.9**	**3.0**	**2.9**	**4.9**	**89.2**							121	4
**60**	1.9	**1.6**	**96.5**	**2.5**	**2.3**	**3.7**	**91.5**							116	3
**90**	2.0	**1.4**	**96.7**	**2.1**	**1.9**	**4.9**	**91.2**							125	4
**5h**	2.9	**2.1**	**95.1**	**2.8**	**2.5**	**4.4**	**90.3**							134	4
**24h**	2.5	**1.6**	**96.0**	**1.5**	**1.4**	**7.0**	**90.1**							41	10

Scherrer size calculation is only reliable for contents > 20%M, must be in range 30 nm-350 nm and the size > 3 x esd

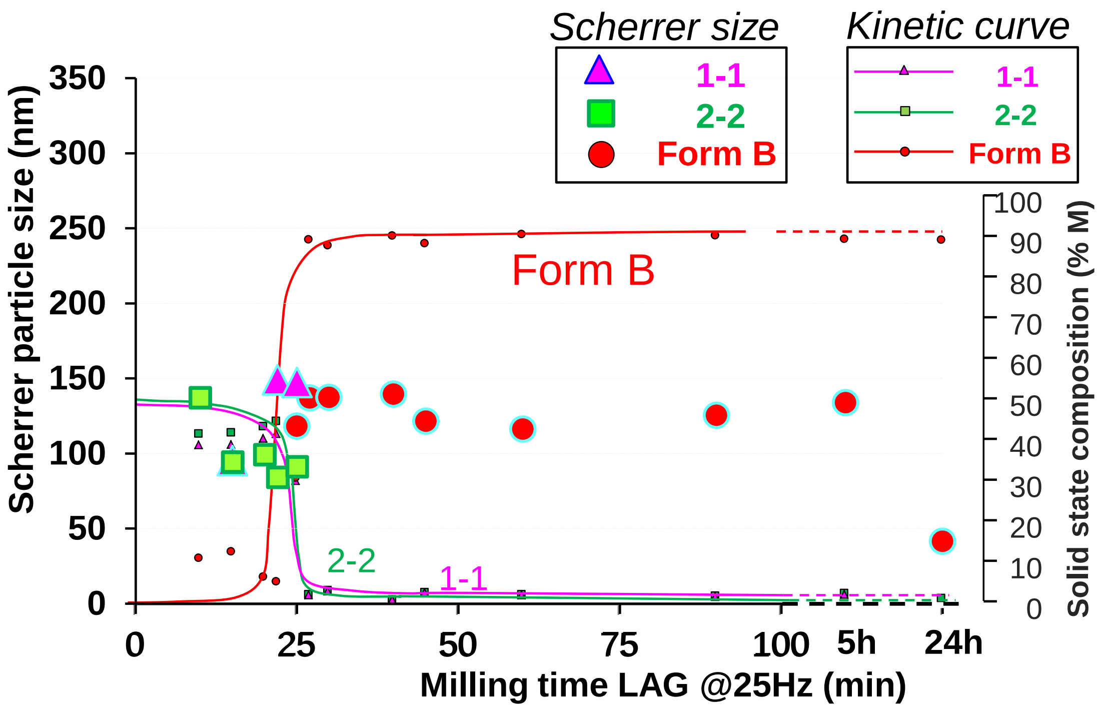

Figure S14: Scherrer size (nm) for the homodimers **1-1**, **2-2** and the reaction product **Form B** determined at different grinding times of the experimental kinetic stiudy at 25 Hz . **Form A** is not formed by LAG experiments. Scherrer size determination is only reliable for compounds > 20%M and their estimated standard deviations (esd) are much smaller than the symbols used. Y-axis on the right is expressed as %M composition of **1-1**, **2-2** and **Form B**.

5.5 Kinetics LAG studies of 1-1 and 2-2 with 2 mol % dbu at 20 Hz

This section presents the characterization of the reaction product of the kinetic ball mill LAG studies at 20 Hz using single point experiments. The solid-state DCC reaction was performed following the procedure described in Section 3.1. The solid-state DCC reaction was run at different grinding times ranging from 5 min up to 24 h to investigate the kinetic profile leading to thermodynamic equilibrium. The chemical composition of the reaction product was determined by HPLC (Table S 8 and Figure S16a). The phase composition of the reaction product was calculated from the Rietveld refinement of the PXRD scans (Table S 9 and Figure S16b). The crystal size of **1-1**, **2-2** and **Form B** in the product was calculated using the Scherrer calculation applied to the Rietveld refinement (Table S10 and Figure S17). The PXRD scans are displayed on Figure S15.

Table S 8 Experimentals details of single point grinding experiments of solid state DCC reaction of **1-1** and **2-2** performed in the presence of 2%M **dbu** under LAG conditions at 20 Hz. Experiments are run from 5 min up to 24 h. The chemical composition of reaction products is determined by HPLC analysis. The HPLC conditions are shown below. This data is plotted on Figure S16a.

Kinetics of ball mill LAG reaction @20Hz					
[1-1]+[2-2]+2%M dbu + 50μL MeCN					
Reagents:	$(2NO_2PhS)_2$	$(4ClPhS)_2$	**HPLC conditions** [λ=259(8nm) ref 550(100nm)]		
MW:	308.33	287.23	Zorbax SB C18, 1.8 μm; 4.6mm ID x50 mm		
%M:	50%M	50%M	**A**: H_2O+0.1% HCOOH; **B**: MeCN+0.1%HCOOH		
mmol:	0.34 mmol	0.34 mmol	0-2 min 75-85%B; 2ml/min; 60°C; run time=2.0 min		
mgs:	104.38 mg	97.63 mg	**HPLC results**		
grinding time @ 20 Hz	$(2NO_2PhS)_2$ weighed	$(4ClPhS)_2$ weighed	$(2NO_2PhS)_2$ (**1-1**)	Product (**1-2**)	$(4ClPhS)_2$ (**2-2**)
min	mg	mg	%M	%M	%M
**0**	Expected %M from weighing		50.0	0.0	50.0
**15**	104.82	97.70	49.4	**0.6**	50.0
**30**	104.76	97.71	48.8	**1.2**	50.0
**35**	104.81	97.65	48.3	**3.4**	48.3
**37**	104.85	97.64	28.9	**39.7**	30.4
**40**	104.84	97.67	32.9	**35.1**	32.0
**45**	104.86	97.7	14.8	**70.7**	15.5
**48**	104.80	97.66	2.5	**95.4**	2.1
**50**	104.82	97.70	3.1	**94.8**	2.3
**60**	104.79	97.63	2.1	**96.4**	1.5
**90**	104.84	97.67	1.9	**96.5**	1.6
**300**	104.79	97.64	1.7	**97.1**	1.2
**24h**	104.82	97.68	2.5	**95.9**	1.6

Table S 9 Solid state composition of the reaction products calculated using Rietveld refimenment from PXRD scans of single point experiments of the solid state DCC reaction of **1-1** and **2-2** performed in the presence of 2%M **dbu** under LAG conditions at 20 Hz. Experiments are run from 5 min up to 24 h. This data is plotted on Figure S16b. The PXRD scans used for the Rietveld refinement are shown on Figure S15

Kinetics of ball mill LAG reaction @20Hz<br>0.68 mmol DCC ([1-1]+[2-2] + 2%M dbu+50μL MeCN)														
grinding time @ 20Hz	HPLC			PXRD: Rietveld refinement										
	1-1	2-2	1-2	1-1		2-2		Form A		Form B		Rwp Rietveld refinement Goodness	chisq Rietveld refinement Fit Index
(min)	%M	%M	%M	%M	e.s.d. mol%	%M	e.s.d. mol%	%M	e.s.d. mol%	%M	e.s.d. mol%		
**0**	**50.0**	**50.0**	**0.0**	**50.0**		**50.0**		**0.0**		**0.0**			
**15**	49.4	50.0	**0.6**	**43.8**	0.6	**40.9**	0.6	**6.5**	1.0	**8.8**	0.7	*16.4*	*4.2*
**30**	48.8	50.0	**1.2**	**44.6**	0.6	**41.6**	0.6	**6.7**	0.9	**7.0**	0.6	*18.3*	*3.4*
**35**	48.3	48.3	**3.4**	**44.9**	0.7	**41.9**	0.7	**5.0**	1.0	**8.2**	0.8	*13.7*	*3.4*
**37**	28.9	30.4	**39.7**	**32.5**	0.6	**30.3**	0.6	**2.7**	0.9	**34.5**	0.7	*7.9*	*2.0*
**40**	32.9	32.0	**35.1**	**31.9**	0.5	**29.8**	0.4	**6.1**	0.8	**32.1**	0.5	*13.0*	*2.2*
**45**	14.8	15.5	**70.7**	**16.8**	0.6	**15.7**	0.5	**6.1**	1.1	**61.4**	1.0	*11.1*	*1.9*
**48**	2.5	2.1	**95.4**	**1.6**	0.5	**1.5**	0.5	**2.9**	0.8	**93.9**	1.0	*12.7*	*3.2*
**50**	3.1	2.3	**94.8**	**4.0**	0.6	**3.7**	0.5	**4.2**	0.8	**88.1**	1.0	*13.9*	*2.5*
**60**	2.1	1.5	**96.4**	**2.8**	0.9	**2.6**	0.8	**5.2**	0.9	**89.4**	1.4	*15.4*	*2.7*
**90**	1.9	1.6	**96.5**	**2.5**	0.8	**2.3**	0.7	**3.3**	0.7	**91.9**	1.2	*13.5*	*2.4*
**300**	1.7	1.2	**97.1**	**1.6**	0.7	**1.5**	0.6	**4.8**	0.7	**92.1**	1.1	*15.6*	*2.8*
**24h**	2.5	1.6	**95.9**	**1.2**	0.6	**1.1**	0.5	**5.7**	0.8	**92.1**	1.1	*16.5*	*3.0*

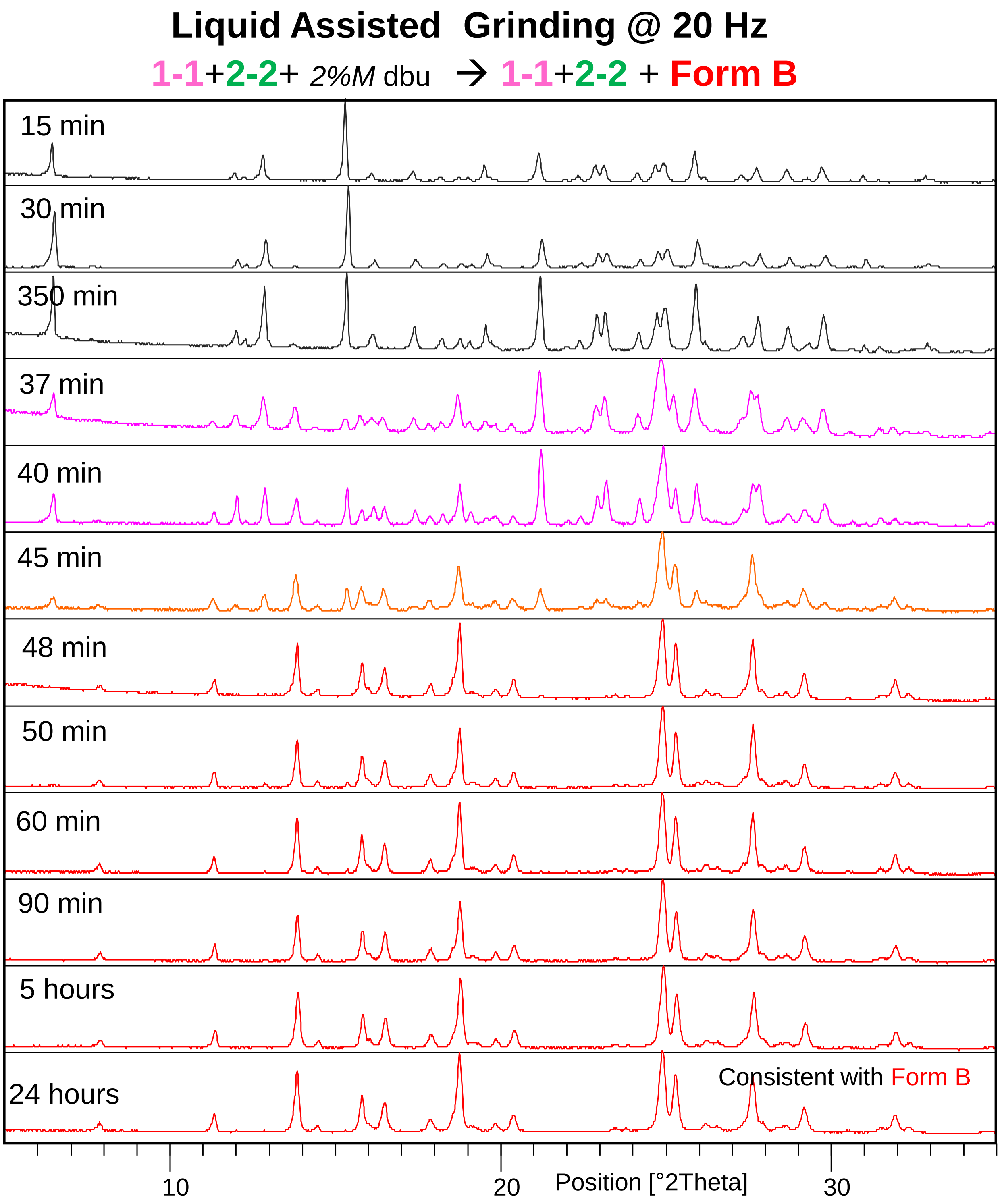

Figure S 15 PXRD scans of the reaction product of the kinetic study of a ball mill LAG reaction (50µL MeCN) at 20 Hz. Each PXRD scan was obtained from a single point experiment. The PXRD sample was prepared immediately after completion of the grinding experiment, and scanned between 5 and 45º (2θ). Typical PXRD scans are shown on Figure S 6.

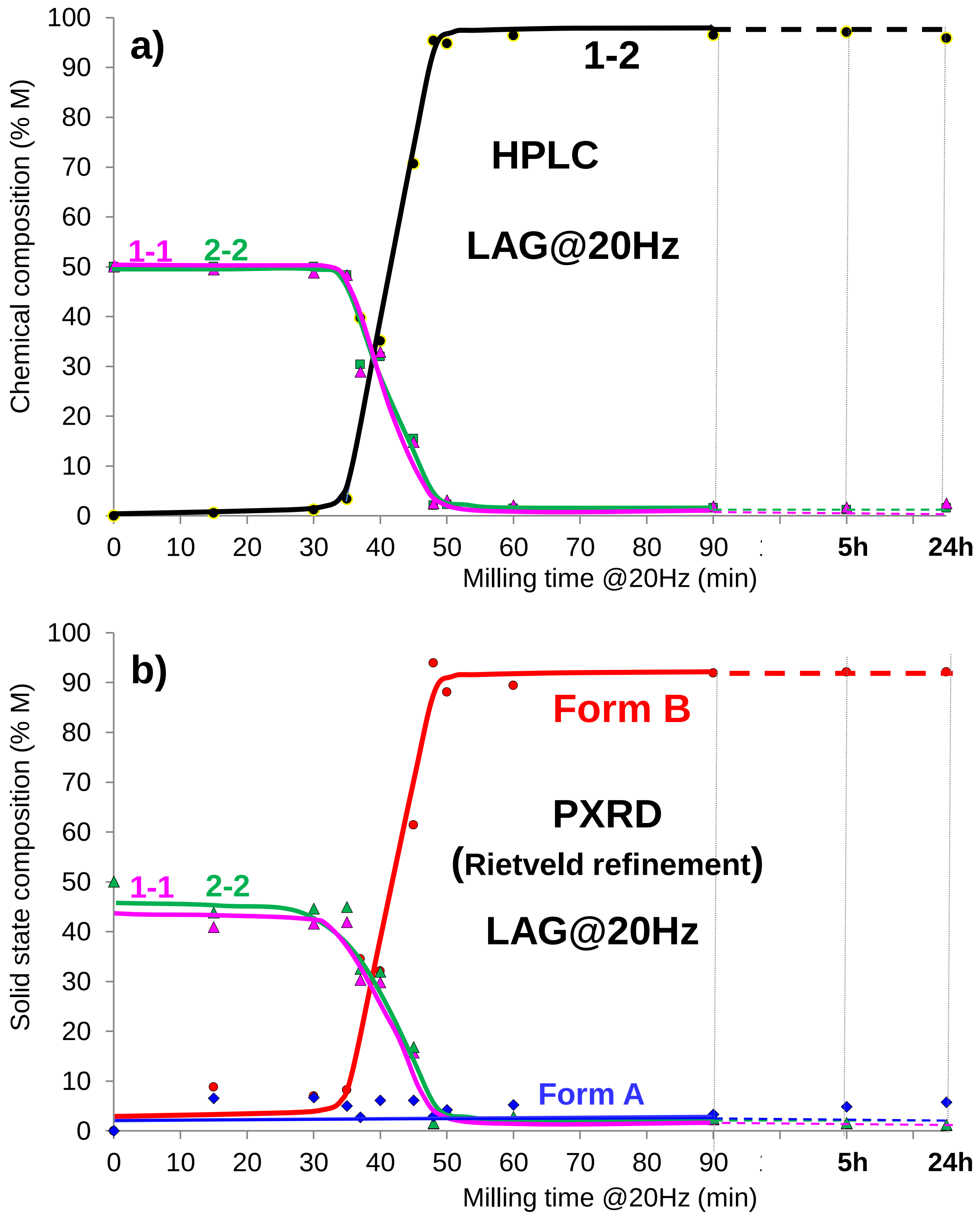

Figure S 16 Kinetic plots of the solid state DCC LAG reaction at 20 Hz of **1-1** and **2-2** in the presence of 2%M **dbu** leading to thermodynamic equilibrium and quantitative formation of **Form B**. a) chemical composition determined by HPLC; b) solid state composition calculated by the Rietveld refinement from PXRD data.

Table S 10 Crystal Scherrer size of the starting materials (**1-1** and **2-2**) and the product **Form B** in relation to the chemical and solid state composition of each single point experiment of the LAG reaction at 20Hz.

Kinetics of ball mill LAG reaction @20Hz															
0.68 mmol DCC ([1-1]+[2-2] + 2%M dbu+50μL MeCN															
grinding time @ 20Hz	HPLC			Rietveld refinement				Scherrer size							
	1-1	2-2	1-2	1-1	2-2	Form A	Form B	1-1		2-2		Form A		Form B	
min	%M	%M	%M	%M	%M	%M	%M	nm	e.s.d. nm	nm	e.s.d. nm	nm	e.s.d. nm	nm	e.s.d. nm
**15**	49.4	**50.0**	**0.6**	**43.8**	**40.9**	**6.5**	**8.8**			135	*5*				
**30**	48.8	**50.0**	**1.2**	**44.6**	**41.6**	**6.7**	**7.0**			127	*4*				
**35**	48.3	**48.3**	**3.4**	**44.9**	**41.9**	**5.0**	**8.2**			161	*8*				
**37**	28.9	**30.4**	**39.7**	**32.5**	**30.3**	**2.7**	**34.5**	108	*8*	106	*6*			**118**	*9*
**40**	32.9	**32.0**	**35.1**	**31.9**	**29.8**	**6.1**	**32.1**	325	*73*	106	*5*			**139**	*10*
**45**	14.8	**15.5**	**70.7**	**16.8**	**15.7**	**6.1**	**61.4**	302	*76*	56	*5*			**98**	*4*
**48**	2.5	**2.1**	**95.4**	**1.6**	**1.5**	**2.9**	**93.9**								
**50**	3.1	**2.3**	**94.8**	**4.0**	**3.7**	**4.2**	**88.1**							**151**	*5*
**60**	2.1	**1.5**	**96.4**	**2.8**	**2.6**	**5.2**	**89.4**							**167**	*6*
**90**	1.9	**1.6**	**96.5**	**2.5**	**2.3**	**3.3**	**91.9**							**139**	*4*
**5h**	1.7	**1.2**	**97.1**	**1.6**	**1.5**	**4.8**	**92.1**							**115**	*3*
**24h**	2.5	**1.6**	**95.9**	**1.2**	**1.1**	**5.7**	**92.1**								

Scherrer size calculation is only reliable for contents > 20%M, must be in range 30 nm-350 nm and the size > 3 x esd

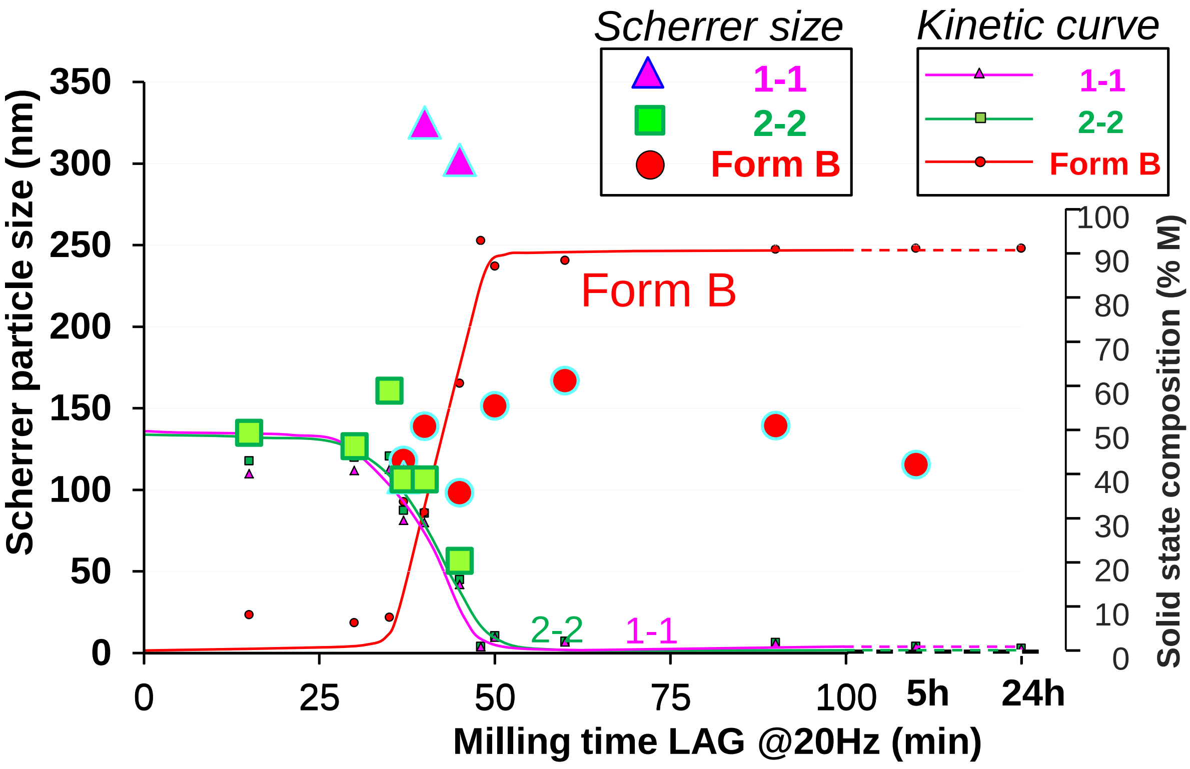

Figure S17 Scherrer size (nm) for the homodimers **1-1**, **2-2** and the reaction product **Form B** determined at different grinding times of the experimental kinetic stiudy at 20 Hz . **Form A** is not formed by LAG experiments. Scherrer size determination is only reliable for compounds > 20%M and their estimated standard deviations (esd) are much smaller than the symbols used. Y-axis on the right is expressed as %M composition of **1-1**, **2-2** and **Form B**.

5.6 Kinetics LAG studies of 1-1 and 2-2 with 2 mol % dbu at 15 Hz

This section presents the characterization of the reaction product of the kinetic ball mill LAG studies at 15 Hz using single point experiments. The solid-state DCC reaction was performed following the procedure described in Section 3.1. The solid-state DCC reaction was run at different grinding times ranging from 5 min up to 24 h to investigate the kinetic profile leading to thermodynamic equilibrium. The chemical composition of the reaction product was determined by HPLC (Table S11 and Figure S19a). The phase composition of the reaction product was calculated from the Rietveld refinement of the PXRD scans (Table S12 and Figure S19b). The crystal size of **1-1**, **2-2** and **Form B** in the product was calculated using the Scherrer calculation applied to the Rietveld refinement (Table S13 and Figure S20). The PXRD scans are displayed on Figure S18.

Table S 11 Experimentals details of single point grinding experiments of solid state DCC reaction of **1-1** and **2-2** performed in the presence of 2%M **dbu** under LAG conditions at 15 Hz. Experiments are run from 5 min up to 24 h. The chemical composition of reaction products is determined by HPLC analysis. The HPLC conditions are shown below. This data is plotted on Figure S19a.

Kinetics of ball mill LAG reaction @**15Hz**					
[**1-1**]+[**2-2**]+2%M dbu + 50μL MeCN					
Reagents:	**$(2NO_2PhS)_2$**	**$(4ClPhS)_2$**	**HPLC conditions** [λ=259(8nm) ref 550(100nm)]		
MW:	308.33	287.23	Zorbax SB C18, 1.8 μm; 4.6mm ID x50 mm		
%M:	50%M	50%M	**A**: H_2O+0.1% HCOOH; **B**: MeCN+0.1%HCOOH		
mmol:	0.34 mmol	0.34 mmol	0-2 min 75-85%B; 2ml/min; 60°C; run time=2.0 min		
mgs:	104.38 mg	97.63 mg	**HPLC results**		
grinding time @ 15 Hz	$(2NO_2PhS)_2$ weighed	$(4ClPhS)_2$ weighed	$(2NO_2PhS)_2$ (**1-1**)	Product (**1-2**)	$(4ClPhS)_2$ (**2-2**)
min	mg	mg	%M	%M	%M
**0**	Expected %M from weighing		50.0	0.0	50.0
**30**	104.81	97.65	47.2	**0.6**	52.2
**45**	104.85	97.69	49.0	**1.5**	49.6
**50**	104.81	97.65	47.4	**1.0**	51.5
**55**	104.80	97.65	45.0	**13.2**	41.7
**60**	104.81	97.68	26.4	**47.9**	25.7
**70**	104.81	97.64	34.7	**34.2**	31.1
**75**	104.80	97.71	18.9	**60.0**	21.4
**90**	104.81	97.62	6.0	**88.5**	5.5
**100**	104.80	97.66	1.7	**97.0**	1.3
**120**	104.83	97.69	4.8	**90.7**	4.6
**150**	104.84	97.69	1.3	**97.8**	0.9
**180**	104.82	97.70	1.7	**97.1**	1.2
**240**	104.84	97.67	2.0	**96.6**	1.4
**300**	104.81	97.63	1.7	**96.9**	1.3
**24h**	104.79	97.64	3.0	**95.4**	1.7

Table S 12 Solid state composition of the reaction products calculated using Rietveld refimenment from PXRD scans of single point experiments of the solid state DCC reaction of **1-1** and **2-2** performed in the presence of 2%M **dbu** under LAG conditions at 15 Hz. Experiments are run from 5 min up to 24 h. This data is plotted on Figure S19b. The PXRD scans used for the Rietveld refinement are shown on Figure S18.

Kinetics of ball mill LAG reaction @15Hz														
0.68 mmol DCC ([1-1]+[2-2] + 2%M dbu+50µL MeCN														
**grinding time @15Hz**	**HPLC**			**PXRD: Rietveld refinement**										
	**1-1**	**2-2**	**1-2**	**1-1**		**2-2**		**Form A**		**Form B**		Rwp Rietveld refinement Goodness	chisq Rietveld refinement Fit Index
(min)	%M	%M	%M	**%M**	e.s.d. mol%	**%M**	e.s.d. mol%	**%M**	e.s.d. mol%	**%M**	e.s.d. mol%		
0	50.0	50.0	0.0	**50.0**		**50.0**		**0.0**		**0.0**			
**30**	47.2	52.2	**0.6**	**47.7**	0.7	**44.4**	0.6	**5.1**	0.9	**2.9**	0.8	*13.7*	*2.4*
**45**	49.0	49.6	**1.5**	**47.1**	0.7	**43.9**	0.7	**5.0**	1.0	**4.1**	0.8	*12.6*	*2.2*
**50**	47.4	51.5	**1.0**	**47.2**	0.7	**44.0**	0.6	**4.4**	0.9	**4.3**	0.8	*13.8*	*2.4*
**55**	45.0	41.7	**13.2**	**44.0**	0.5	**41.0**	0.5	**6.7**	0.9	**8.4**	0.5	*13.2*	*2.4*
**60**	18.2	18.2	**63.7**	**42.0**	0.6	**39.2**	0.6	**5.8**	0.9	**13.0**	0.6	*15.1*	*2.6*
**60**	26.4	25.7	**47.9**	**39.8**	0.6	**37.1**	0.5	**4.8**	0.9	**18.3**	0.5	*11.4*	*1.9*
**70**	34.7	31.1	**34.2**	**35.9**	0.4	**33.5**	0.4	**6.4**	0.8	**24.1**	0.5	*12.5*	*2.3*
**75**	18.9	21.4	**60.0**	**5.8**	0.3	**5.4**	0.3	**4.4**	0.7	**84.3**	0.7	*13.4*	*2.3*
**90**	6.0	5.5	**88.5**	**4.1**	0.3	**3.9**	0.3	**3.3**	0.6	**88.7**	0.7	*15.2*	*2.7*
**100**	1.7	1.3	**97.0**	**1.0**	0.7	**1.0**	0.6	**3.1**	0.6	**94.9**	1.1	*13.4*	*2.3*
**120**	4.8	4.6	**90.7**	**5.9**	0.3	**5.5**	0.3	**4.2**	0.7	**84.4**	0.8	*15.4*	*2.6*
**150**	1.3	0.9	**97.8**	**2.2**	0.8	**2.0**	0.8	**4.3**	0.8	**91.5**	1.3	*11.5*	*2.0*
**180**	1.7	1.2	**97.1**	**2.2**	0.7	**2.1**	0.7	**4.8**	0.8	**90.9**	1.2	*11.6*	*2.0*
**240**	2.0	1.4	**96.6**	**1.6**	0.6	**1.4**	0.6	**4.4**	0.7	**92.6**	1.1	*15.1*	*2.7*
**300**	1.7	1.3	**96.9**	**1.1**	0.3	**1.0**	0.3	**3.6**	0.6	**94.3**	0.7	*16.0*	*2.8*
**24h**	3.0	1.7	**95.4**	**0.7**	0.7	**0.7**	0.6	**5.8**	0.7	**92.8**	1.2	*17.4*	*3.1*

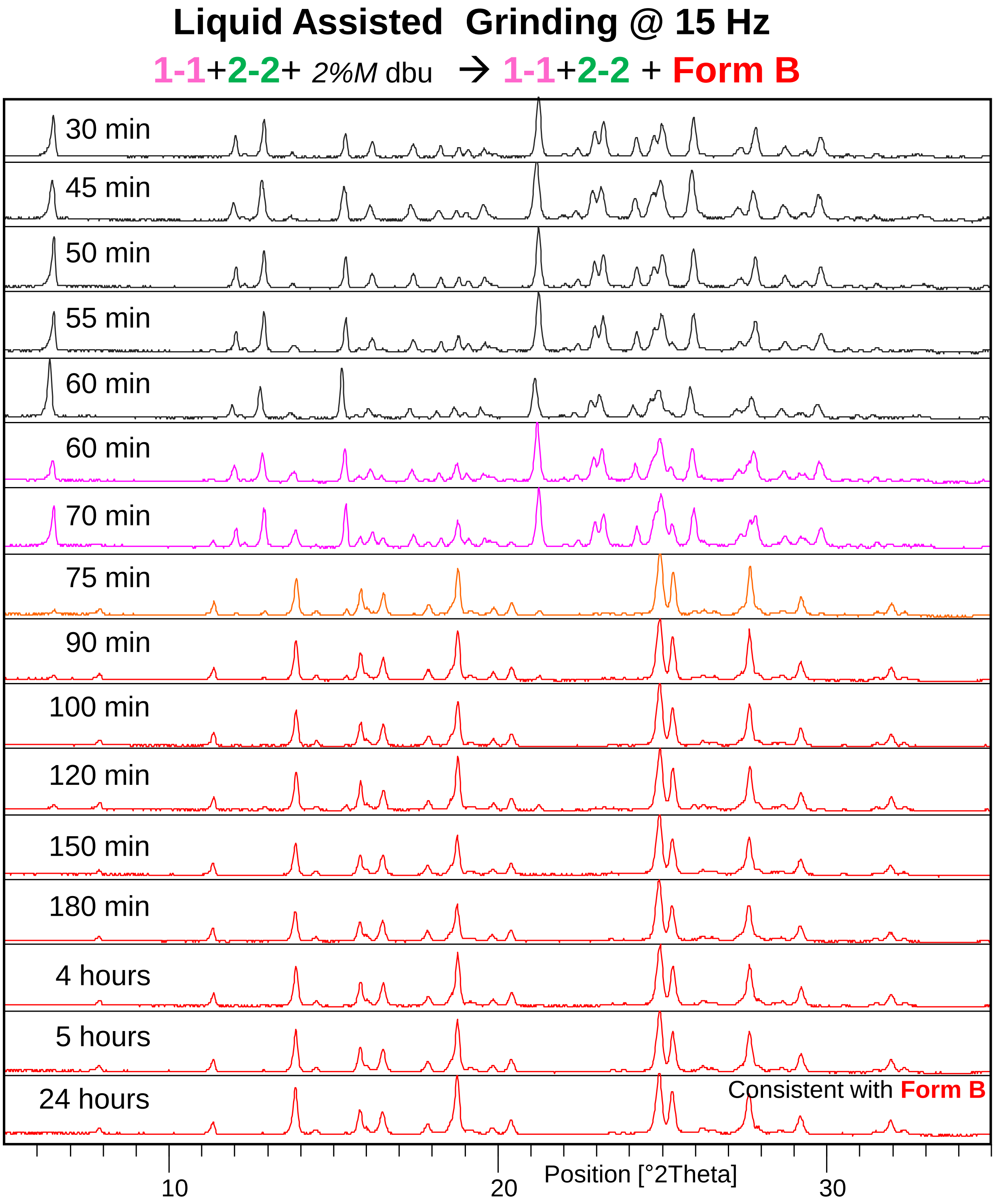

Figure S 18 PXRD scans of the reaction product of the kinetic study of a ball mill LAG reaction (50μL MeCN) at 15 Hz. Each PXRD scan was obtained from a single point experiment. The PXRD sample was prepared immediately after completion of the grinding experiment, and scanned between 5 and 45° (2θ). Typical PXRD scans are shown on Figure S 6.

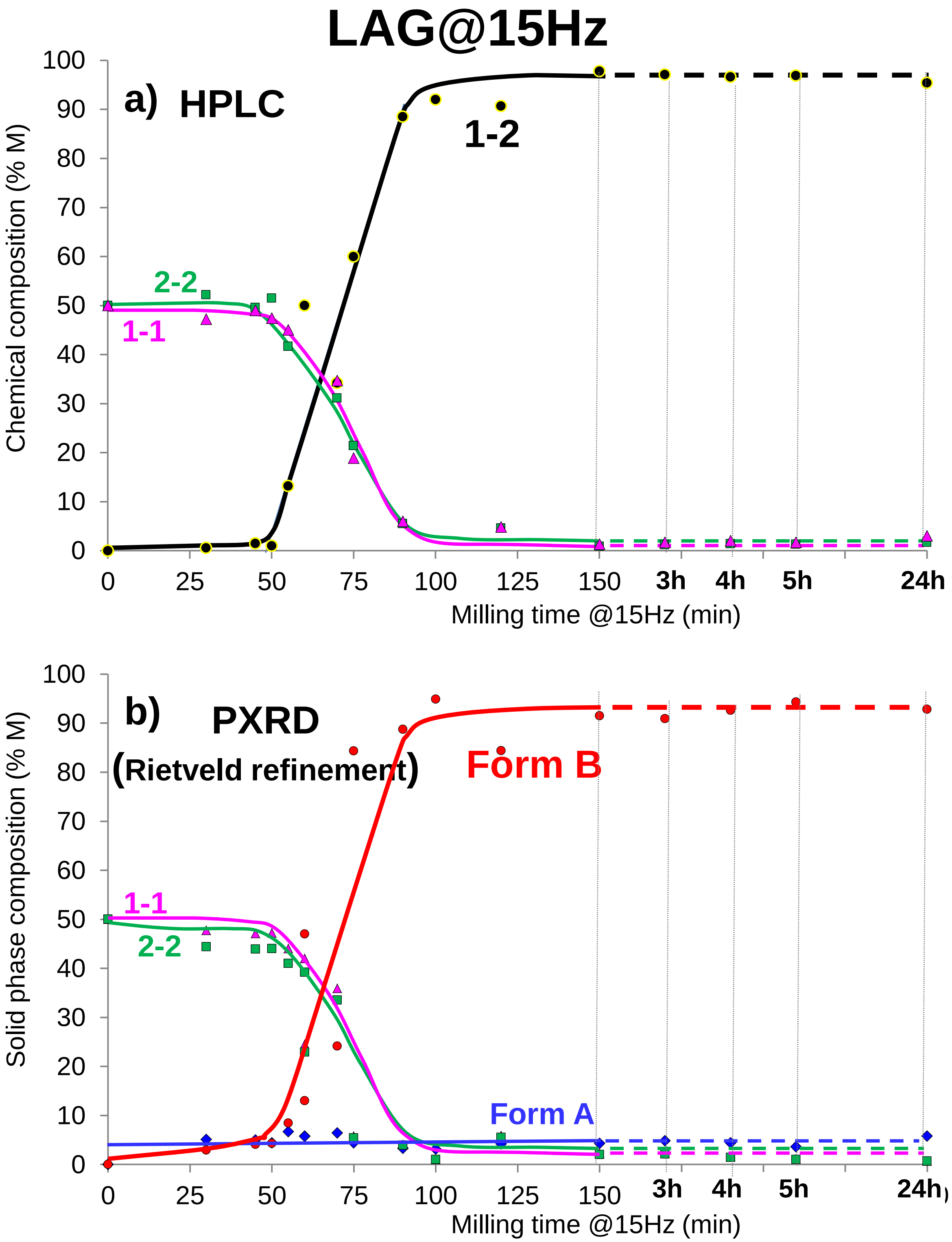

Figure S 19 Kinetic plots of the solid state DCC LAG reaction at 15 Hz of **1-1** and **2-2** in the presence of 2%M **dbu** leading to thermodynamic equilibrium and quantitative formation of **Form B**. a) chemical composition determined by HPLC; b) solid state composition calculated by the Rietveld refinement from PXRD data.

Table S 13 Crystal Scherrer size of the starting materials (**1-1** and **2-2**) and the product **Form B** in relation to the chemical and solid state composition of each single point experiment of the LAG reaction at 15Hz.

Kinetics of ball mill LAG reaction @**15Hz**

0.68 mmol DCC ([1-1]+[2-2] + 2%M dbu+50μL MeCN

grinding time @ 30Hz	HPLC			Rietveld refinement				Scherrer size							
	1-1	2-2	1-2	1-1	2-2	Form A	Form B	1-1		2-2		Form A		Form B	
min	%M	%M	%M	%M	%M	%M	%M	nm	e.s.d. nm	nm	e.s.d. nm	nm	e.s.d. nm	nm	e.s.d. nm
30	47.2	52.2	0.6	47.7	44.4	5.1	2.9	274	22	104	4				
45	49.0	49.6	1.5	47.1	43.9	5.0	4.1	167	9	77	2				
50	47.4	51.5	1.0	47.2	44.0	4.4	4.3	325	31	125	4				
55	45.0	41.7	13.2	44.0	41.0	6.7	8.4	325	33	109	3				
60	18.2	18.2	63.7	42.0	39.2	5.8	13.0	325	45	98	3				
60	26.4	25.7	47.9	39.8	37	4.8	18.3			103	5			137	6
70	34.7	31.1	34.2	35.9	33.5	6.4	24.1	325	51	100	3			125	9
75	18.9	21.4	60.0	5.8	5.4	4.4	84.3							160	5
90	6.0	5.5	88.5	4.1	3.9	3.3	88.7							163	5
100	1.7	1.3	97.0	1.0	1.0	3.1	94.9							146	3
120	4.8	4.6	90.7	5.9	5.5	4.2	84.4							156	5
150	1.3	0.9	97.8	2.2	2.0	4.3	91.5							134	4
3h	1.7	1.2	97.1	2.2	2.1	4.8	90.9							126	3
4h	2.0	1.4	96.6	1.6	1.4	4.4	92.6							128	3
5h	1.7	1.3	96.9	1.1	1.0	3.6	94.3							132	3
24h	3.0	1.7	95.4	0.7	0.7	5.8	92.8								

Scherrer size calculation is only reliable for contents > 20%M, must be in range 30 nm-350 nm and the size > 3 x esd

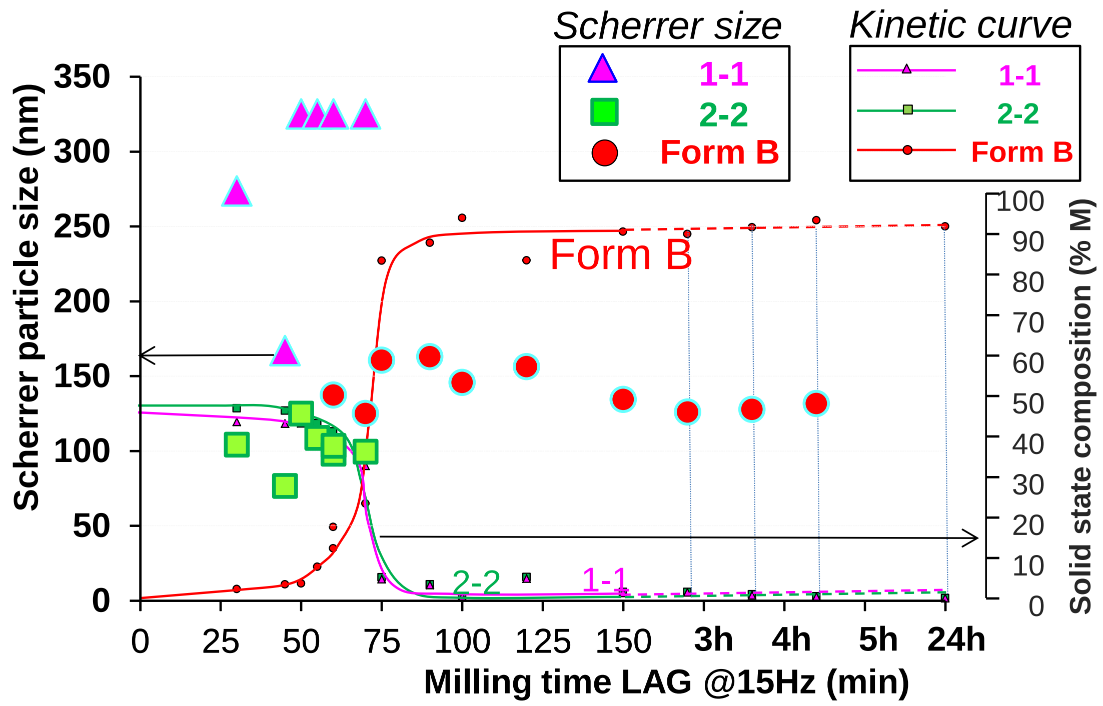

Figure S20 Scherrer size (nm) for the homodimers **1-1**, **2-2** and the reaction product **Form B** determined at different grinding times of the experimental kinetic stiudy at 15 Hz . **Form A** is not formed by LAG experiments. Scherrer size determination is only reliable for compounds > 20%M and their estimated standard deviations (esd) are much smaller than the symbols used. Y-axis on the right is expressed as %M composition of **1-1**, **2-2** and **Form B**.

5.7 Lack of homogeneity in LAG at low grinding frequency (15 Hz)

Table S 14: Examples of poor homogeneity of the chemical composition of the kinetic points along the sigmoidal segment (55min-75 min) when grinding at 15 Hz: the powder is found caked around the balls (see pictures) resulting in different chemical composition on the balls and inside the jar. During the initial grinding period (30 min and 50 min) the chemical composition of the powder in various locations is homogenous: **1-1**& **2-2** have not reacted yet. The values tabulated on this table are plotted in Figure S21.

Kinetics of ball mill **LAG** reaction @**15Hz- Chemical composition**						
[**1-1**]+[**2-2**]+2%M dbu + 50µL MeCN						
grinding time @ 15 Hz	Position inside jar	replicates in same part of jar	$(2NO_2PhS)_2$ (**1-1**)	Product (**1-2**)	$(4ClPhS)_2$ (**2-2**)	Pictures of jar with powder opened just after compeltion of grinding
min			%M	%M	%M	
**30**	jar /male	1	47.2	**0.6**	52.2	no pictures available
**30**	jar /male	2	46.1	**0.6**	53.2	
**30**	jar /female	1	53.2	**0.4**	46.4	
**30**	jar /female	2	48.1	**0.4**	51.5	
**30**	ball bearing 1	1	47.6	**0.6**	51.8	
**30**	ball bearing 2	1	48.3	**0.4**	51.3	
**30**	ball bearing 2	2	47.7	**0.3**	52.0	
**50**	jar /male	1	46.9	**1.4**	51.7	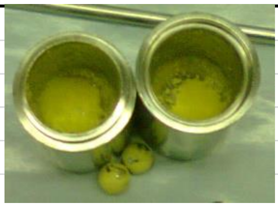
**50**	jar /male	2	47.4	**1.0**	51.5	
**50**	jar /female	1	56.0	**0.4**	43.6	
**50**	jar /female	2	46.8	**1.9**	51.2	
**50**	ball bearing 1	1	48.4	**1.8**	49.8	
**50**	ball bearing 2	1	48.3	**1.8**	49.9	
**55**	jar /male	1	44.8	**3.9**	51.3	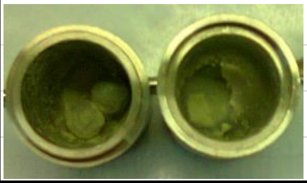
**55**	jar /female	1	45.1	**4.9**	50.0	
**55**	ball bearing 1	1	43.0	**17.6**	39.4	
**55**	ball bearing 2	2	45.0	**13.2**	41.7	
**60**	jar /male	1	32.7	**30.0**	37.3	no pictures available
**60**	jar /female	1	37.1	**22.9**	40.0	
**60**	ball bearing 1	1	26.4	**47.9**	25.7	
**70**	jar /male	1	40.6	**17.8**	41.6	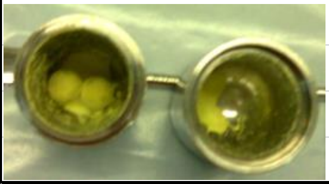
**70**	jar /female	1	38.2	**24.8**	37.0	
**70**	ball bearing 1	1	34.7	**34.2**	31.1	
**70**	ball bearing 2	1	34.0	**35.6**	30.5	
**75**	jar /male	1	18.9	**60.0**	21.4	no pictures available
**75**	jar /female	1	22.8	**52.1**	24.7	
**75**	ball bearing 1	1	3.0	**94.4**	2.6	
**75**	ball bearing 2	1	2.6	**95.3**	2.1	

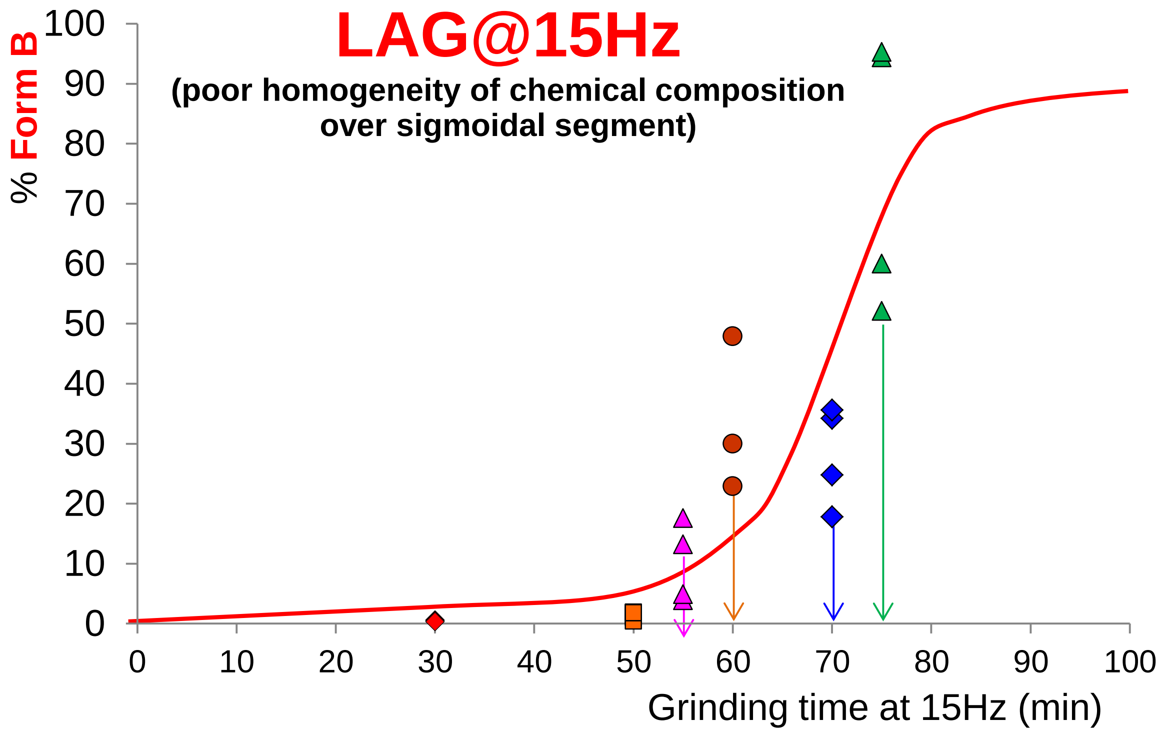

Figure S21 Display of poor homogeneity of the composition of the individual grinding samples at different grinding periods once **1-1** and **2-2** start reacting in the presence of the catalyst **dbu** under LAG conditions at 15Hz; dbu may be poorly distributed to all the powder when using the low grinding frequencies and therefore results in different reaction efficiency. The values of the composition is tabulated in Table S14

5.8 Comparison between kinetics LAG studies at 30, 25, 20 and 15 Hz

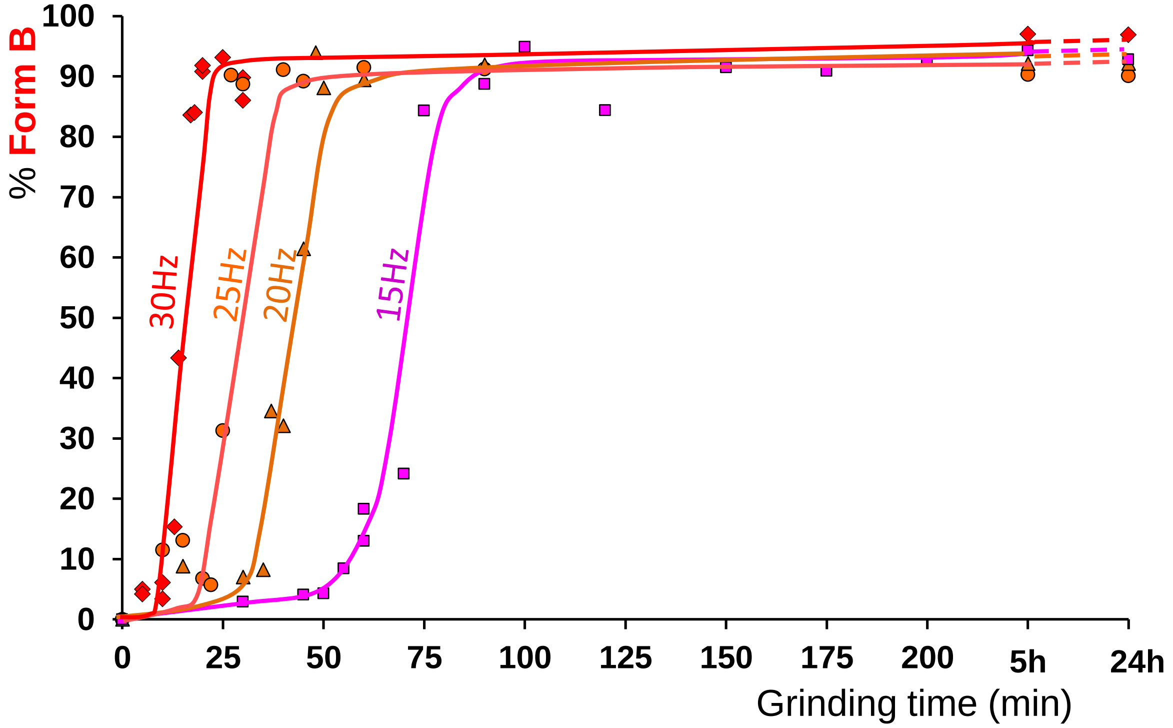

Figure S22 Comparison between the kinetic curves obtained from individual experiments ground between 5 min and 24 hours of the solid state DCC reaction run under LAG conditions at 30Hz, 25Hz, 20Hz and 15Hz. The curves are a guide to the eye only.

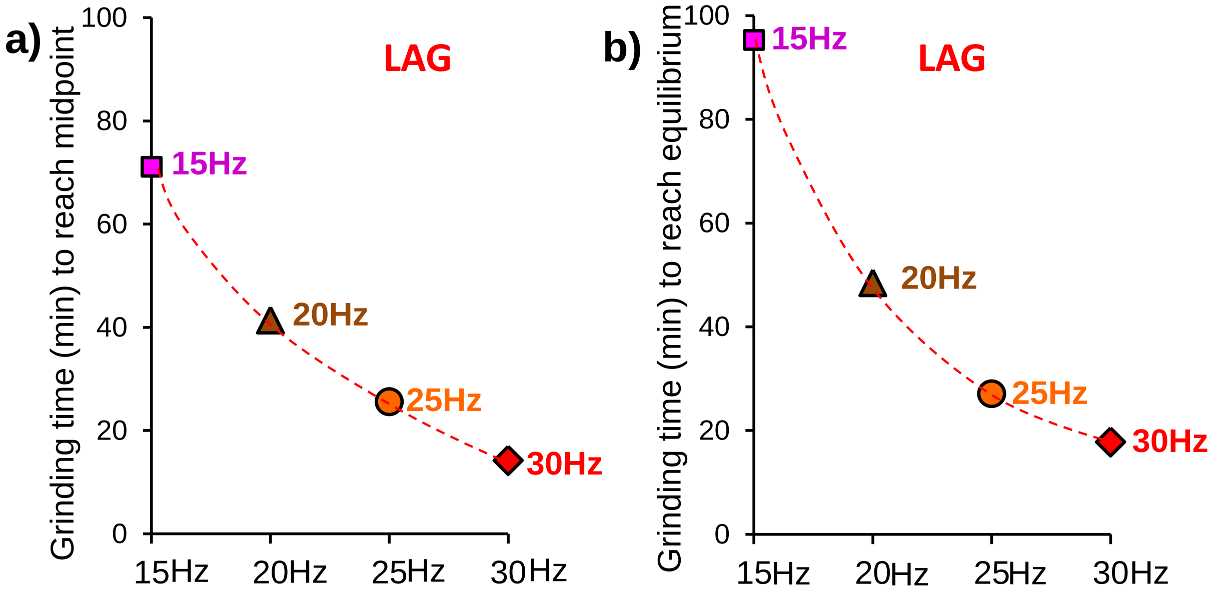

Figure S23 Correlation between the grinding time (min) and the frequency of LAG reaction of **1-1** and **2-2** in presence of dbu for a) the midpoint of the sigmoidal curve and b) the start of the thermodynamic equilibrium of the kinetic curves obtained for LAG experiments ground at 15Hz, 20Hz, 25Hz and 30Hz

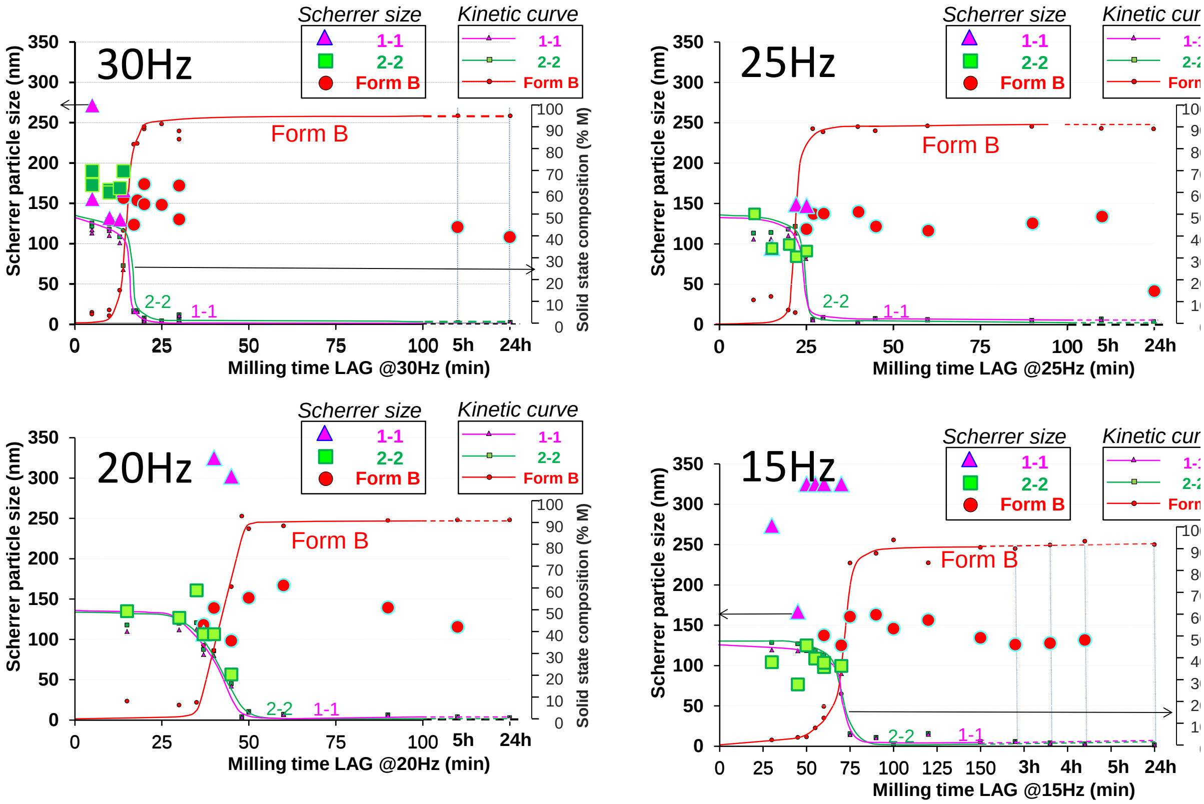

Figure S24 Comparison of the Scherrer particle size of **1-1**, **2-2** and **Form B** in ball mill LAG reactions performed at 30Hz, 25Hz, 20Hz and 15Hz.

6 Kinetic studies of ball mill NG of 1-1 and 2-2 with 2 mol % dbu

Ball mill NG reactions were performed starting from equimolar mixtures of **1-1** and **2-2** with 2 mol % dbu as a base catalyst.

6.1 Kinetics NG studies of 1-1 and 2-2 with 2 mol % dbu at 30 Hz

This section presents the characterization of the reaction product of the kinetic ball mill NG studies at 30 Hz using single point experiments. The solid-state DCC reaction was performed following the procedure described in Section 3.1. The solid-state DCC reaction was run at different grinding times ranging from 5 min up to 24 h to investigate the kinetic profile leading to thermodynamic equilibrium.

The chemical composition of the reaction product was determined by HPLC (Table S15 and Figure S26a). The phase composition of the reaction product was calculated from the Rietveld refinement of the PXRD scans (Table S16 and Figure S26b). The crystal size of **1-1**, **2-2** and **Form A** in the product was calculated using the Scherrer calculation applied to the Rietveld refinement (Table S17 and Figure S27). The PXRD scans are displayed on Figure S25.

Table S 15 Experimentals details of single point grinding experiments of the solid-state DCC reaction of **1-1** and **2-2** performed in the presence of 2 mol % **dbu** under ball mill NG conditions at 30 Hz. Experiments are run from 5 min up to 24 h. The chemical composition of reaction products is determined by HPLC analysis. The HPLC conditions are shown below. This data is plotted on Figure S26a.

Kinetics of ball mill **NG** reaction @**30Hz**

[**1-1**]+[**2-2**]+2%M dbu

Reagents:	**$(2NO_2PhS)_2$**	**$(4ClPhS)_2$**	**HPLC conditions** [λ=259(8nm) ref 550(100nm)]		
MW:	308.33	287.23	Zorbax SB C18, 1.8 μm; 4.6mm ID x50 mm		
%M:	50%M	50%M	**A**: H_2O+0.1% HCOOH; **B**: MeCN+0.1%HCOOH		
mmol:	0.34 mmol	0.34 mmol	0-2 min 75-85%B; 2ml/min; 60°C; run time=2.0 min		
mgs:	104.38 mg	97.63 mg	**HPLC results**		
grinding time @ 30 Hz	$(2NO_2PhS)_2$ weighed	$(4ClPhS)_2$ weighed	$(2NO_2PhS)_2$ (**1-1**)	Product (**1-2**)	$(4ClPhS)_2$ (**2-2**)
min	mg	mg	%M	%M	%M
**0**	Expected %M from weighing		50.0	0.0	50.0
**0**	104.85	97.70	49.0	**0.0**	51.0
**5**	104.88	97.66	50.5	**2.8**	46.7
**5**	104.79	97.69	50.4	**3.0**	46.6
**10**	104.82	97.63	48.0	**4.5**	47.5
**10**	104.85	97.73	48.6	**4.7**	46.7
**15**	104.84	97.66	47.3	**6.2**	46.5
**15**	104.83	97.69	47.2	**6.9**	45.9
**15**	104.88	97.67	46.9	**5.7**	47.5
**15**	104.85	97.64	47.5	**5.1**	47.3
**15**	104.83	97.62	47.5	**6.5**	46.0
**15**	104.78	97.66	48.9	**5.7**	45.3
**20**	104.83	97.63	47.1	**5.3**	47.6
**20**	104.82	97.61	47.7	**7.3**	45.0
**25**	104.88	97.76	47.7	**4.6**	47.7
**25**	104.80	97.66	46.9	**7.3**	45.8
**25**	104.85	97.63	44.2	**10.9**	44.9
**30**	104.71	97.69	45.5	**8.8**	45.7
**32**	104.84	97.62	46.6	**9.1**	44.3
**33**	104.81	97.59	4.9	**90.5**	4.6
**34**	104.82	97.65	5.7	**88.8**	5.5
**36**	104.85	97.64	3.0	**94.2**	2.7
**37**	104.81	97.58	4.7	**90.9**	4.4
**40**	104.85	97.62	2.8	**94.9**	2.3
**45**	104.88	97.69	2.0	**96.7**	1.3
**50**	104.83	97.65	1.5	**97.2**	1.3
**300**	104.83	97.65	1.3	**97.2**	1.5
**24h**	104.85	97.64	1.3	**97.2**	1.5

Table S 16 Solid-state composition of the reaction products calculated using Rietveld refimenment from PXRD scans of single point experiments of the solid-state DCC reaction of **1-1** and **2-2** performed in the presence of 2 mol % **dbu** under ball mill NG conditions at 30 Hz. Experiments are run from 5 min up to 24 h. This data is plotted on Figure S26b. The PXRD scans used for the Rietveld refinement are shown on Figure S25,

Kinetics of ball mill **NG** reaction @**30Hz**														
0.68 mmol DCC ([**1-1**]+[**2-2**] + 2%M dbu														
**grinding time @ 30Hz**	**HPLC**			**PXRD: Rietveld refinement**										
	**1-1**	**2-2**	**1-2**	**1-1**		**2-2**		**Form A**		**Form B**		Rwp Rietveld refinement Goodness	chisq Rietveld refinement Fit Index
(min)	%M	%M	%M	%M	e.s.d. mol%	%M	e.s.d. mol%	%M	e.s.d. mol%	%M	e.s.d. mol%		
**0**	**50.0**	**50.0**	**0.0**	**50.0**		**50.0**		**0.0**		**0.0**			
**5**	50.5	46.7	**2.8**	**46.3**	0.6	**43.1**	0.6	**6.3**	0.9	**4.3**	0.7	*11.6*	*9.9*
**10**	48.6	46.7	**4.7**	**46.6**	0.6	**43.3**	0.6	**5.9**	0.9	**4.2**	0.7	*11.0*	*9.4*
**15**	47.3	46.5	**6.2**	**46.4**	0.7	**43.1**	0.6	**6.1**	1.0	**4.3**	0.8	*10.3*	*8.6*
**15**	46.9	47.5	**5.7**	**43.2**	2.2	**40.1**	2.0	**10.2**	3.1	**6.6**	2.6	*7.5*	*3.2*
**15**	47.5	46.0	**6.5**	**46.5**	0.7	**43.2**	0.6	**6.0**	1.0	**4.3**	0.7	*10.2*	*8.6*
**15**	48.9	45.3	**5.7**	**46.7**	0.7	**43.5**	0.6	**5.6**	0.9	**4.2**	0.7	*10.1*	*8.4*
**20**	47.1	47.6	**5.3**	**44.9**	0.5	**41.7**	0.5	**7.4**	0.6	**5.9**	0.7	*9.4*	*7.8*
**20**	47.7	45.0	**7.3**	**44.8**	0.6	**41.5**	0.6	**7.0**	0.8	**6.7**	0.7	*9.4*	*8.0*
**30**	45.5	45.7	**8.8**	**40.1**	1.6	**37.1**	1.5	**19.8**	2.0	**2.9**	2.0	*7.5*	*3.2*
**33**	4.9	4.6	**90.5**	**2.7**	0.3	**2.5**	0.3	**92.8**	0.5	**2.0**	0.4	*6.8*	*5.7*
**34**	5.7	5.5	**88.8**	**4.6**	0.4	**4.3**	0.4	**88.1**	0.6	**3.0**	0.4	*6.3*	*5.1*
**36**	3.0	2.7	**94.2**	**1.3**	0.3	**1.2**	0.3	**94.4**	0.6	**3.1**	0.4	*6.7*	*5.5*
**37**	4.7	4.4	**90.9**	**2.2**	0.3	**2.1**	0.3	**93.8**	0.5	**1.9**	0.3	*7.1*	*6.1*
**40**	2.8	2.3	**94.9**	**1.0**	0.3	**0.9**	0.3	**95.9**	0.5	**2.3**	0.3	*7.0*	*5.9*
**45**	2.0	1.3	**96.7**	**3.1**	2.0	**2.9**	1.9	**88.6**	3.3	**5.5**	2.3	*6.2*	*2.5*
**50**	1.5	1.3	**97.2**	**0.5**	0.3	**0.5**	0.3	**96.8**	0.5	**2.2**	0.3	*7.1*	*6.1*
**300**	1.3	1.5	**97.2**	**0.8**	0.5	**0.7**	0.5	**94.7**	0.8	**3.9**	0.5	*6.1*	*5.0*
**24h**	1.3	1.5	**97.2**	**1.0**	0.9	**1.0**	0.8	**92.2**	1.6	**92.2**	1.6	*6.0*	*2.5*

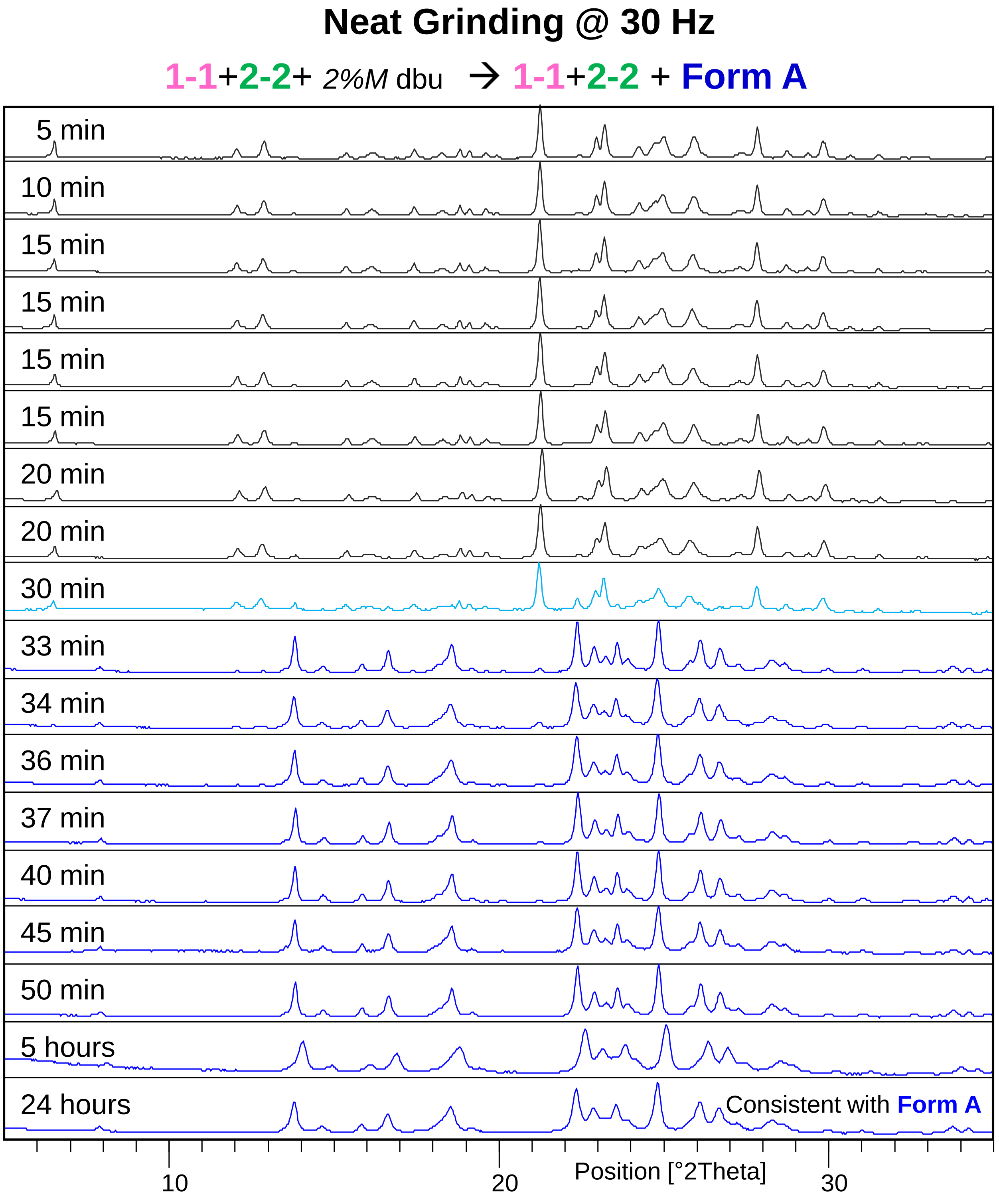

Figure S 25 PXRD scans of the reaction product of the kinetic study of a ball mill NG reaction at 30 Hz. Each PXRD scan was obtained from a single point experiment. The PXRD sample was prepared immediately after completion of the grinding experiment, and scanned between 5 and 45º (2θ). Typical PXRD scans are shown on Figure S 6.

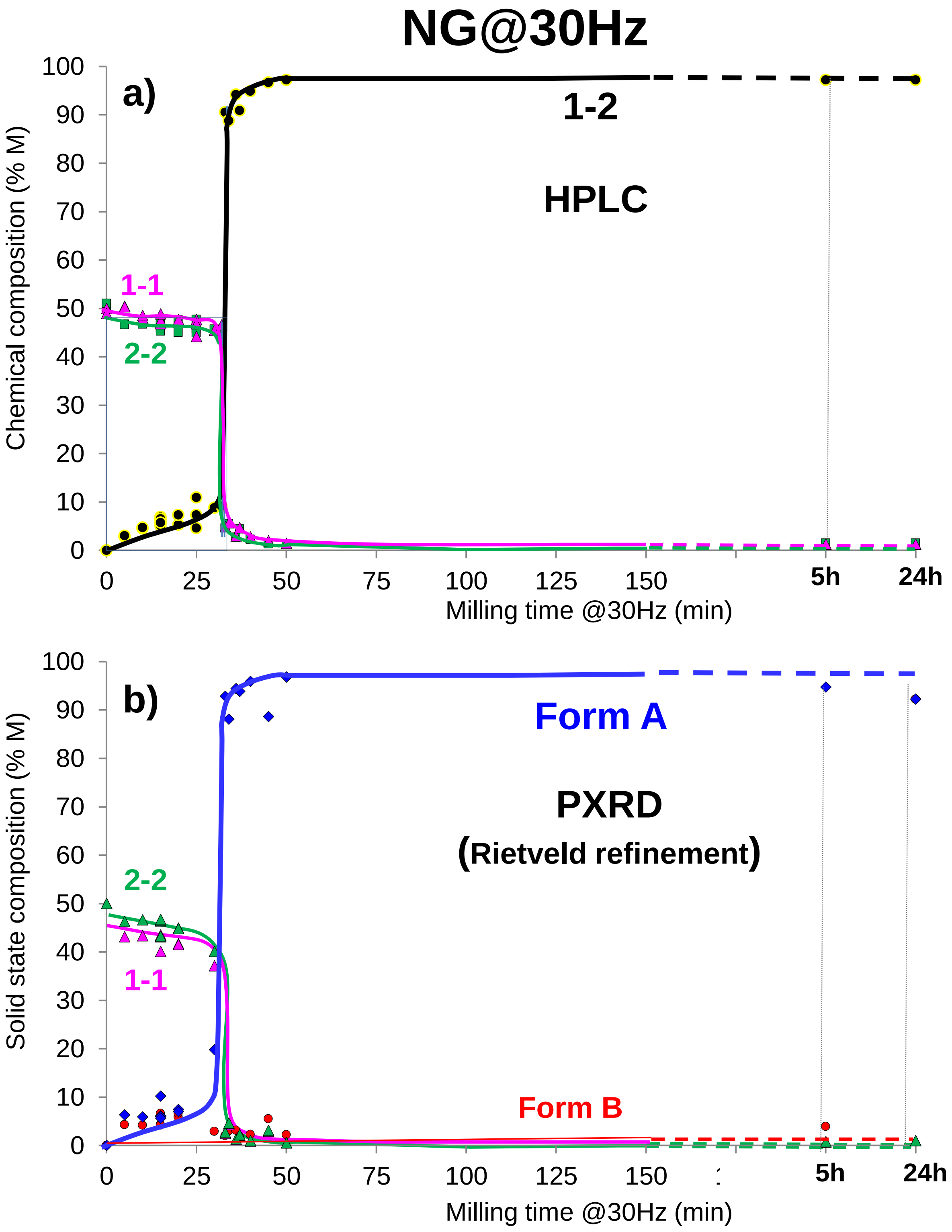

Figure S 26 Kinetic plots of the solid-state DCC ball mill NG reaction at 30 Hz of **1-1** and **2-2** in the presence of 2 mol % dbu leading to thermodynamic equilibrium and quantitative formation of **Form A**. a) chemical composition determined by HPLC; b) solid-state composition calculated by the Rietveld refinement from PXRD data.

Table S 17 Crystal Scherrer size of the starting materials (**1-1** and **2-2**) and the product **Form A** in relation to the chemical and solid-state composition of each single point experiment of the ball mill NG reaction at 30Hz.

Kinetics of ball mill NG reaction @30Hz															
0.68 mmol DCC ([1-1]+[2-2] + 2%M dbu															
**grinding time @ 30Hz**	**HPLC**			**Rietveld refinement**				**Scherrer size**							
	**1-1**	**2-2**	**1-2**	**1-1**	**2-2**	**Form A**	**Form B**	**1-1**		**2-2**		**Form A**		**Form B**	
min	%M	%M	%M	%M	%M	%M	%M	**nm**	e.s.d. nm	**nm**	e.s.d. nm	**nm**	e.s.d. nm	**nm**	e.s.d. nm
**5**	50.5	**46.7**	**2.8**	46.3	43.1	6.3	4.3	95	*3*	300	*23*				
**10**	48.6	**46.7**	**4.7**	46.6	43.3	5.9	4.2	89	*3*	289	*15*				
**15**	47.3	**46.5**	**6.2**	46.4	43.1	6.1	4.3	82	*3*	295	*16*				
**15**	46.9	**47.5**	**5.7**	43.2	40.1	10.2	6.6	60	*6*	149	*18*				
**15**	47.5	**46.0**	**6.5**	46.5	43.2	6.0	4.3	74	*2*	208	*9*				
**15**	48.9	**45.3**	**5.7**	46.7	43.5	5.6	4.2	82	*3*	241	*11*				
**20**	47.1	**47.6**	**5.3**	44.9	41.7	7.4	5.9	63	*2*	141	*5*				
**20**	47.7	**45.0**	**7.3**	44.8	41.5	7.0	6.7	52	*2*	124	*4*				
**30**	45.5	**45.7**	**8.8**	40.1	37.1	19.8	2.9	44	*5*	117	*13*				
**33**	4.9	**4.6**	**90.5**	2.7	2.5	92.8	2.0					**96**	*2*		
**34**	5.7	**5.5**	**88.8**	4.6	4.3	88.1	3.0					**58**	*1*		
**36**	3.0	**2.7**	**94.2**	1.3	1.2	94.4	3.1					**63**	*1*		
**37**	4.7	**4.4**	**90.9**	2.2	2.1	93.8	1.9					**90**	*2*		
**40**	2.8	**2.3**	**94.9**	1.0	0.9	95.9	2.3					**91**	*2*		
**45**	2.0	**1.3**	**96.7**	3.1	2.9	88.6	5.5					**76**	*5*		
**50**	1.5	**1.3**	**97.2**	0.5	0.5	96.8	2.2					**83**	*1*		
**5h**	1.3	**1.5**	**97.2**	0.8	0.7	94.7	3.9					**47**	*1*		
**24h**	1.3	**1.5**	**97.2**	1.0	1.0	92.2	92.2					**39**	*2*		
Scherrer size calculation is only reliable for contents > 20%M, must be in range 30 nm-350 nm and the size > 3 x esd															

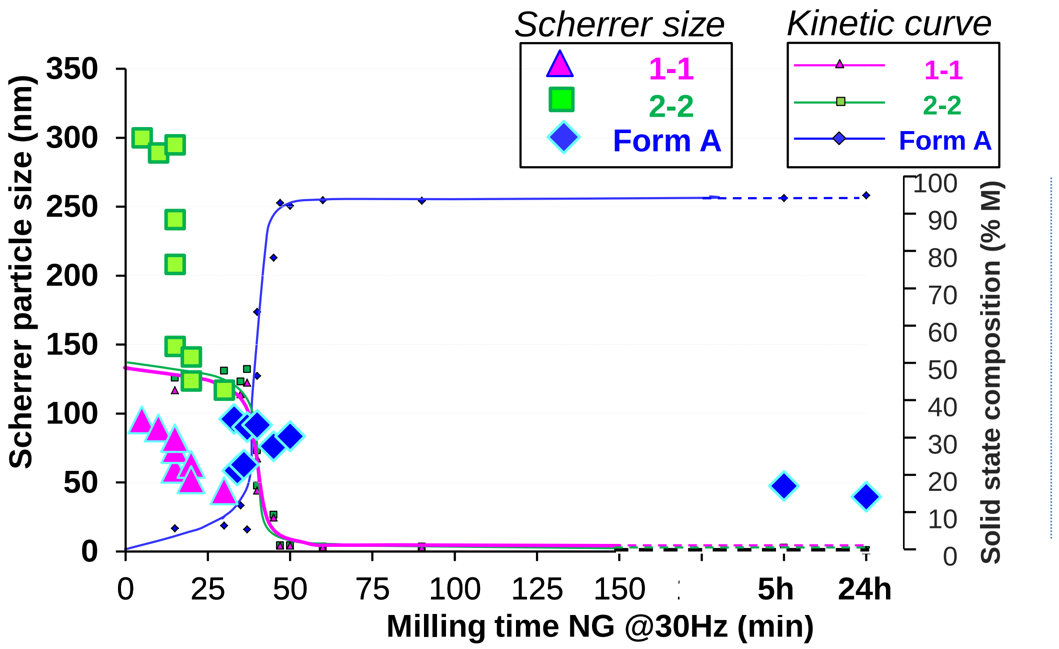

Figure S27 Scherrer size (nm) for the homodimers **1-1**, **2-2** and the reaction product **Form A** determined at different grinding times of the experimental kinetic stiudy at 30 Hz. **Form B** is not formed by NG experiments. Scherrer size determination is only reliable for compounds > 20 mol % and their estimated standard deviations (esd) are much smaller than the symbols used. Y-axis on the right is expressed as mol % composition of **1-1**, **2-2** and **Form A**.

6.2 Kinetic NG studies of 1-1 and 2-2 with 2 mol % dbu at 25 Hz

This section presents the characterization of the reaction product of the kinetic ball mill NG studies at 25 Hz using single point experiments. The solid-state DCC reaction was performed following the procedure described in Section 3.1. The solid-state DCC reaction was run at different grinding times ranging from 5 min up to 24 h to investigate the kinetic profile leading to thermodynamic equilibrium. The chemical composition of the reaction product was determined by HPLC (Table S18 and Figure S29a). The phase composition of the reaction product was calculated from the Rietveld refinement of the PXRD scans (Table S19 and Figure S29b). The crystal size of **1-1**, **2-2** and **Form A** in the product was calculated using the Scherrer calculation applied to the Rietveld refinement (Table S20 and Figure S30). The PXRD scans are displayed on Figure S28.

Table S 18 Experimentals details of single point grinding experiments of the solid-state DCC reaction of **1-1** and **2-2** performed in the presence of 2 mol % **dbu** under ball mill NG conditions at 25 Hz. Experiments are run from 5 min up to 24 h. The chemical composition of reaction products is determined by HPLC analysis. The HPLC conditions are shown below. This data is plotted on Figure S29a.

Kinetics of ball mill **NG** reaction @**25Hz** [**1-1**]+[**2-2**]+2%M dbu					
Reagents:	**$(2NO_2PhS)_2$**	**$(4ClPhS)_2$**	**HPLC conditions** [λ=259(8nm) ref 550(100nm)]		
MW:	308.33	287.23	Zorbax SB C18, 1.8 μm; 4.6mm ID x50 mm		
%M:	50%M	50%M	**A**: H_2O+0.1% HCOOH; **B**: MeCN+0.1%HCOOH		
mmol:	0.34 mmol	0.34 mmol	0-2 min 75-85%B; 2ml/min; 60°C; run time=2.0 min		
mgs:	104.38 mg	97.63 mg	**HPLC results**		
grinding time @25 Hz	$(2NO_2PhS)_2$ weighed	$(4ClPhS)_2$ weighed	$(2NO_2PhS)_2$ (**1-1**)	Product (**1-2**)	$(4ClPhS)_2$ (**2-2**)
min	mg	mg	%M	%M	%M
**0**	Expected %M from weighing		50.0	0.0	50.0
**15**	104.85	97.66	47.6	**4.5**	47.9
**30**	104.88	97.60	46.3	**7.1**	46.6
**35**	104.83	97.66	40.1	**19.4**	40.5
**37**	104.83	97.63	47.4	**4.9**	47.7
**40**	104.86	97.64	16.7	**66.1**	17.2
**40**	104.85	97.65	6.5	**87.7**	5.9
**45**	104.86	97.68	4.0	**92.0**	4.0
**47**	104.66	97.63	2.8	**94.6**	2.6
**50**	104.81	97.67	3.4	**93.4**	3.2
**60**	104.83	97.73	1.2	**97.6**	1.2
**90**	104.85	97.70	1.0	**98.0**	1.1
**300**	104.84	97.70	1.0	**98.0**	1.1
**24h**	104.80	97.74	0.5	**98.4**	1.0

Table S 19 Solid-state composition of the reaction products calculated using Rietveld refimenment from PXRD scans of single point experiments of the solid-state DCC reaction of **1-1** and **2-2** performed in the presence of 2 mol % **dbu** under NG conditions at 25 Hz. Experiments are run from 5 min up to 24 h. This data is plotted on Figure S29b. The PXRD scans used for the Rietveld refinement are shown on Figure S28.

Kinetics of ball mill **NG** reaction @**25Hz**													
0.68 mmol DCC ([**1-1**]+[**2-2**] + 2%M dbu													
**grinding time @ 30Hz**	**HPLC**			**PXRD: Rietveld refinement**									
	**1-1**	**2-2**	**1-2**	**1-1**		**2-2**		**Form A**		**Form B**		Rwp Rietveld refinement Goodness	chisq Rietveld refinement Fit Index
(min)	%M	%M	%M	**%M**	e.s.d. mol%	**%M**	e.s.d. mol%	**%M**	e.s.d. mol%	**%M**	e.s.d. mol%		
**0**	**50.0**	**50.0**	**0.0**	**50.0**		**50.0**		**0.0**		**0.0**			
**15**	47.6	47.9	**4.5**	**46.4**	0.7	**43.1**	0.6	**6.2**	0.8	**4.3**	0.8	*11.2*	*2.0*
**30**	46.3	46.6	**7.1**	**48.3**	0.6	**44.7**	0.5	**6.9**	0.8	**0.0**	0.4	*12.1*	*2.1*
**35**	40.1	40.5	**19.4**	**45.4**	0.5	**42.0**	0.5	**12.3**	0.7	**0.3**	0.4	*12.5*	*2.1*
**37**	47.4	47.7	**4.9**	**48.7**	0.7	**45.1**	0.7	**5.9**	1.0	**0.3**	0.8	*9.8*	*2.5*
**40**	16.7	17.2	**66.1**	**27.0**	0.4	**24.9**	0.4	**46.9**	0.5	**1.2**	0.4	*9.7*	*1.7*
**40**	6.5	5.9	**87.7**	**17.6**	0.4	**16.3**	0.4	**64.0**	0.5	**2.0**	0.4	*9.1*	*1.6*
**45**	4.0	4.0	**92.0**	**9.8**	0.4	**9.1**	0.4	**78.5**	0.6	**2.6**	0.4	*8.9*	*1.6*
**47**	2.8	2.6	**94.6**	**1.7**	0.3	**1.6**	0.3	**93.1**	0.5	**3.6**	0.4	*7.1*	*1.8*
**50**	3.4	3.2	**93.4**	**1.7**	0.3	**1.6**	0.3	**92.3**	0.6	**4.4**	0.4	*9.2*	*1.7*
**60**	1.2	1.2	**97.6**	**1.3**	0.5	**1.2**	0.5	**93.8**	0.8	**3.7**	0.4	*9.6*	*1.7*
**90**	1.0	1.1	**98.0**	**1.3**	0.5	**1.2**	0.5	**93.7**	0.8	**3.8**	0.4	*10.1*	*1.8*
**300**	1.0	1.1	**98.0**	**1.0**	0.4	**0.9**	0.3	**94.4**	0.6	**3.8**	0.4	*9.7*	*1.7*
**24h**	0.5	1.0	**98.4**	**0.4**	0.2	**0.4**	0.2	**95.1**	0.4	**4.1**	0.4	*10.1*	*1.8*

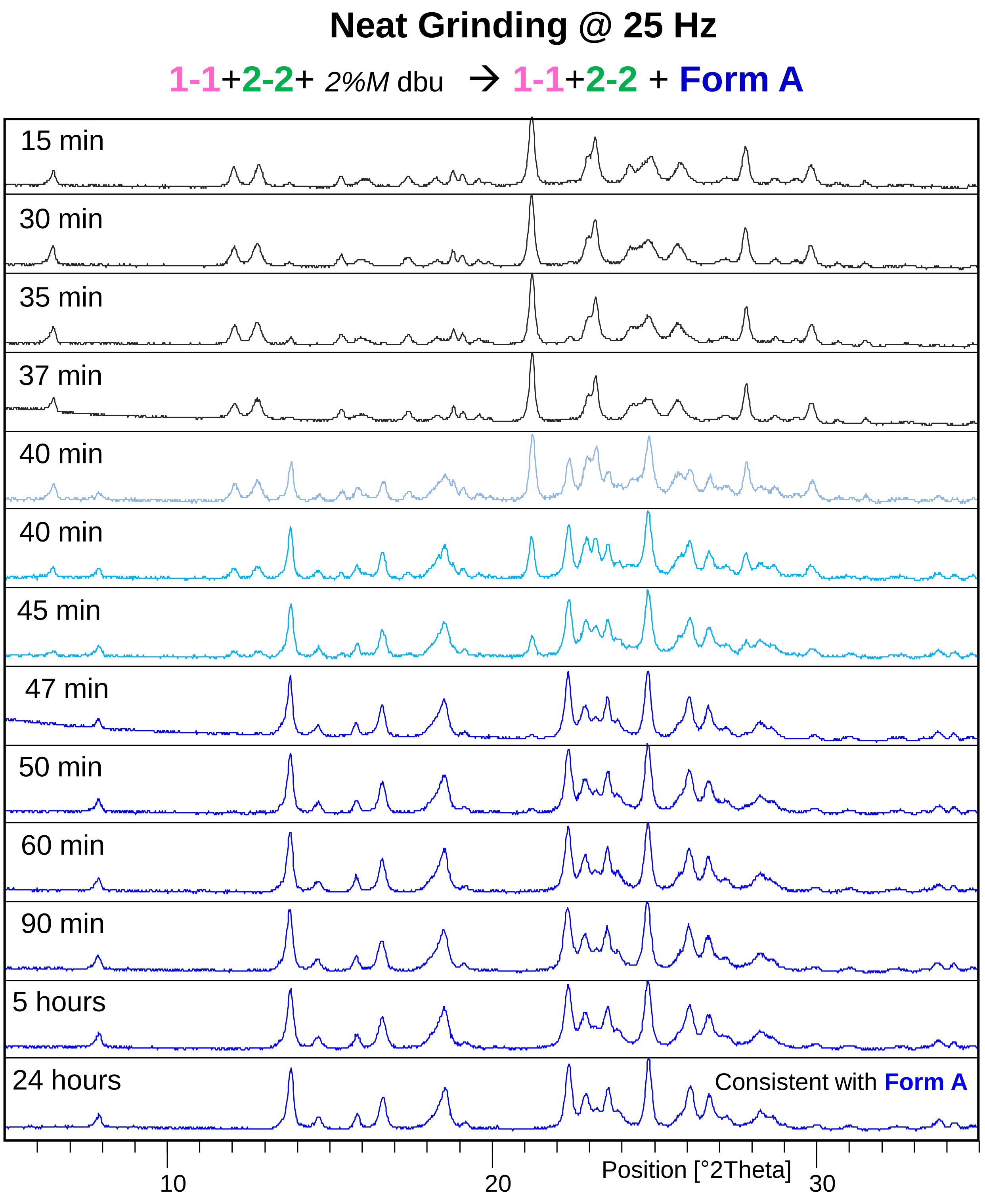

Figure S 28 PXRD scans of the reaction product of the kinetic study of a ball mill NG reaction at 25 Hz. Each PXRD scan was obtained from a single point experiment. The PXRD sample was prepared immediately after completion of the grinding experiment, and scanned between 5 and 45° (2θ). Typical PXRD scans are shown on Figure S 6.

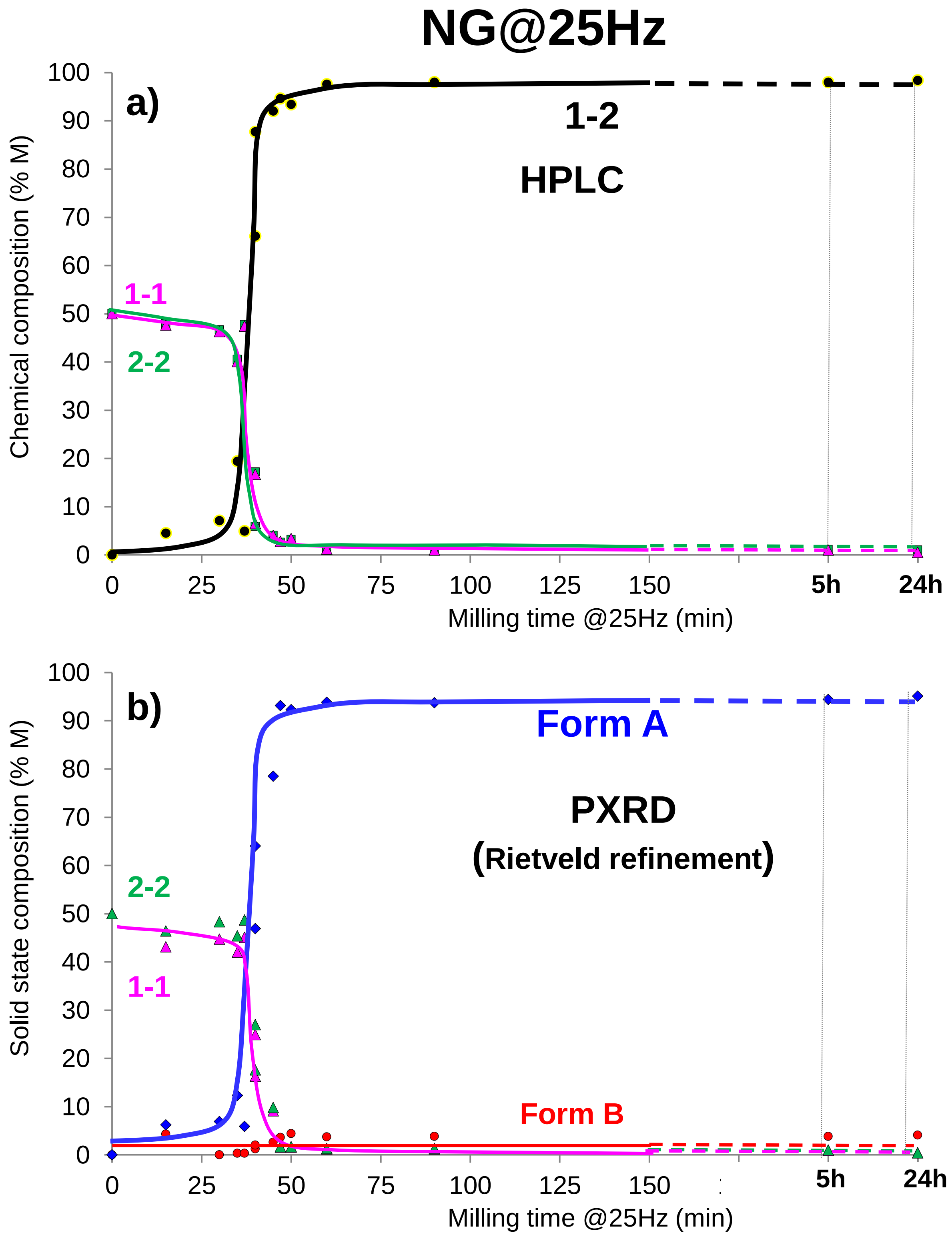

Figure S 29: Kinetic plots of the solid-state DCC NG reaction at 25 Hz of **1-1** and **2-2** in the presence of 2 mol % **dbu** leading to thermodynamic equilibrium and quantitative formation of **Form A**. a) chemical composition determined by HPLC; b) solid-state composition calculated by the Rietveld refinement from PXRD data.

Table S 20 Crystal Scherrer size of the starting materials (**1-1** and **2-2**) and the product **Form A** in relation to the chemical and solid-state composition of each single point experiment of the ball mill NG reaction at 25Hz.

Kinetics of ball mill NG reaction @25Hz															
0.68 mmol DCC ([1-1]+[2-2] + 2%M dbu															
grinding time @ 25Hz	HPLC			Rietveld refinement				Scherrer size							
	1-1	2-2	1-2	1-1	2-2	Form A	Form B	1-1		2-2		Form A		Form B	
min	%M	%M	%M	%M	%M	%M	%M	nm	e.s.d. nm	nm	e.s.d. nm	nm	e.s.d. nm	nm	e.s.d. nm
**15**	47.6	47.9	**4.5**	**46.4**	**43.1**	**6.2**	**4.3**	43	*1*	59	*1*				
**30**	46.3	46.6	**7.1**	**48.3**	**44.7**	**6.9**	**0.0**	34	*1*	60	*1*				
**35**	40.1	40.5	**19.4**	**45.4**	**42.0**	**12.3**	**0.3**	37	*1*	63	*1*				
**37**	47.4	47.7	**4.9**	**48.7**	**45.1**	**5.9**	**0.3**	35	*1*	73	*2*				
**40**	16.7	17.2	**66.1**	**27.0**	**24.9**	**46.9**	**1.2**	36	*1*	60	*2*	**51**	*1*		
**40**	6.5	5.9	**87.7**	**17.6**	**16.3**	**64.0**	**2.0**	40	*2*	66	*3*	**57**	*1*		
**45**	4.0	4.0	**92.0**	**9.8**	**9.1**	**78.5**	**2.6**					**50**	*1*		
**47**	2.8	2.6	**94.6**	**1.7**	**1.6**	**93.1**	**3.6**					**54**	*1*		
**50**	3.4	3.2	**93.4**	**1.7**	**1.6**	**92.3**	**4.4**					**49**	*1*		
**60**	1.2	1.2	**97.6**	**1.3**	**1.2**	**93.8**	**3.7**					**50**	*1*		
**90**	1.0	1.1	**98.0**	**1.3**	**1.2**	**93.7**	**3.8**					**45**	*1*		
**5h**	1.0	1.1	**98.0**	**1.0**	**0.9**	**94.4**	**3.8**					**44**	*1*		
**24h**	0.5	1.0	**98.4**	**0.4**	**0.4**	**95.1**	**4.1**								

Scherrer size calculation is only reliable for contents > 10%M, must be in range 30 nm-350 nm and the size > 3 x esd

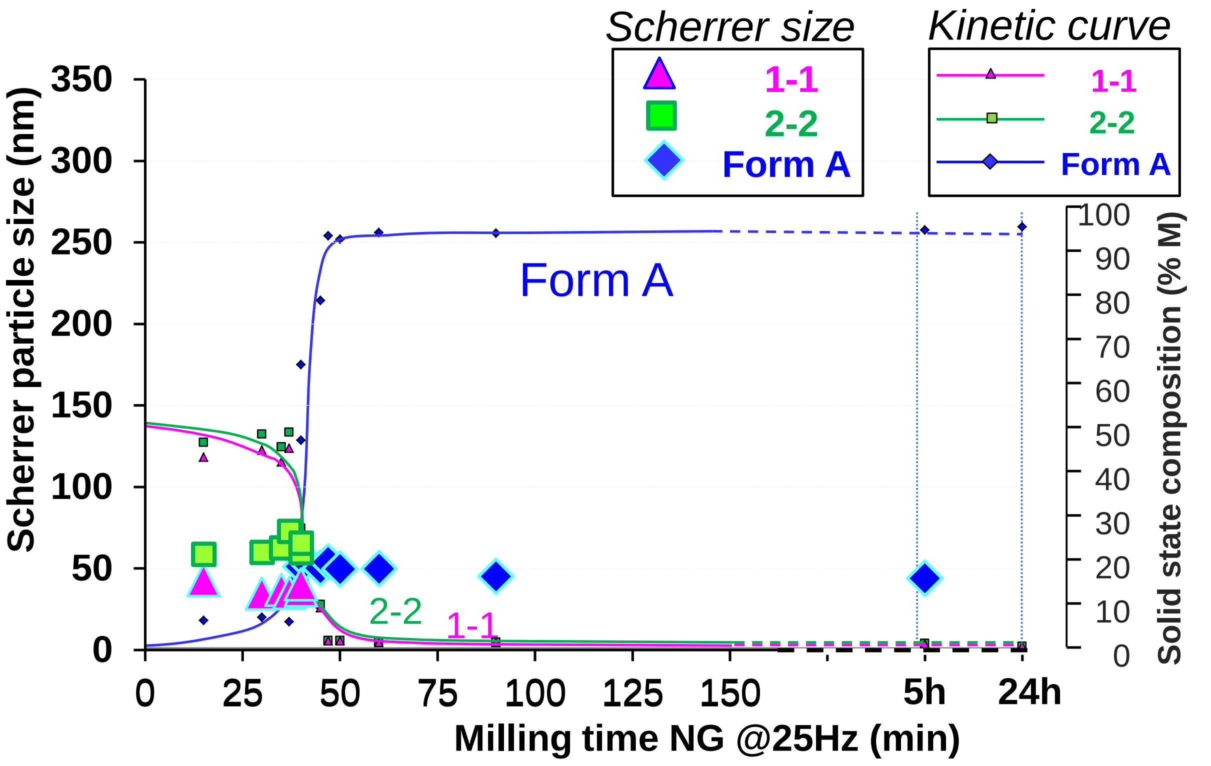

Figure S30 Scherrer size (nm) for the homodimers **1-1**, **2-2** and the reaction product **Form A** determined at different grinding times of the experimental kinetic stiudy at 25 Hz . **Form B** is not formed by NG experiments. Scherrer size determination is only reliable for compounds > 20 mol % and their estimated standard deviations (esd) are much smaller than the symbols used. Y-axis on the right is expressed as mol % composition of **1-1**, **2-2** and **Form A**.

6.3 *Kinetic NG studies of 1-1 and 2-2 with 2 mol % dbu at 20 Hz*

This section presents the characterization of the reaction product of the kinetic ball mill NG studies at 20 Hz using single point experiments. The solid-state DCC reaction was performed following the procedure described in Section 3.1. The solid-state DCC reaction was run at different grinding times ranging from 5 min up to 24 h to investigate the kinetic profile leading to thermodynamic equilibrium. The chemical composition of the reaction product was determined by HPLC (Table S21 and Figure S32a). The phase composition of the reaction product was calculated from the Rietveld refinement of the PXRD scans (Table S22 and Figure S32b). The crystal size of **1-1**, **2-2** and **Form A** in the product was calculated using the Scherrer calculation applied to the Rietveld refinement (Table S23 and Figure S33). The PXRD scans are displayed on Figure S31.

Table S 21 Experimentals details of single point grinding experiments of ball mill NG reaction of **1-1** and **2-2** performed in the presence of 2 mol % **dbu** under ball mill NG conditions at 20 Hz. Experiments are run from 5 min up to 24 h. The chemical composition of reaction products is determined by HPLC analysis. The HPLC conditions are shown below. This data is plotted on Figure S32a.

Kinetics of ball mill **NG** reaction @**20Hz**					
[**1-1**]+[**2-2**]+2%M dbu					
Reagents:	**($2NO_2PhS)_2$**	**$(4ClPhS)_2$**	**HPLC conditions** [λ=259(8nm) ref 550(100nm)]		
MW:	308.33	287.23	Zorbax SB C18, 1.8 μm; 4.6mm ID x50 mm		
%M:	50%M	50%M	**A**: H_2O+0.1% HCOOH; **B**: MeCN+0.1%HCOOH		
mmol:	0.34 mmol	0.34 mmol	0-2 min 75-85%B; 2ml/min; 60^oC; run time=2.0 min		
mgs:	104.38 mg	97.63 mg	**HPLC results**		
grinding time @ 20 Hz	$(2NO_2PhS)_2$ weighed	$(4ClPhS)_2$ weighed	$(2NO_2PhS)_2$ (**1-1**)	Product (**1-2**)	$(4ClPhS)_2$ (**2-2**)
min	mg	mg	%M	%M	%M
**0**	Expected %M from weighing		50.0	0.0	50.0
**30**	104.81	97.64	47.9	**4.1**	48.0
**45**	104.84	97.68	47.4	**4.4**	48.2
**50**	104.82	97.63	29.5	**40.5**	30.1
**60**	104.90	97.67	31.8	**35.9**	32.4
**70**	104.83	97.66	20.1	**59.6**	20.3
**80**	104.85	97.71	12.4	**75.1**	12.4
**90**	104.83	97.66	1.9	**96.2**	1.9
**100**	104.79	97.68	1.2	**97.3**	1.5
**120**	104.83	97.65	3.8	**92.4**	3.8
**150**	104.83	97.66	0.5	**99.2**	0.3
**300**	104.83	97.70	1.0	**97.8**	1.2
**24h**	104.86	97.67	0.9	**98.0**	1.1

Table S 22 Solid-state composition of the reaction products calculated using Rietveld refimenment from PXRD scans of single point experiments of the solid-state DCC reaction of **1-1** and **2-2** performed in the presence of 2 mol % **dbu** under ball mill NG conditions at 20 Hz. Experiments are run from 5 min up to 24 h. This data is plotted on Figure S32b. The PXRD scans used for the Rietveld refinement are shown on Figure S31.

Kinetics of ball mill NG reaction @20Hz<br>0.68 mmol DCC ([1-1]+[2-2] + 2%M dbu														
**grinding time @ 20Hz**	**HPLC**			**PXRD: Rietveld refinement**										
	**1-1**	**2-2**	**1-2**	**1-1**		**2-2**		**Form A**		**Form B**		Rwp Rietveld refinement Goodness	chisq Rietveld refinement Fit Index
(min)	%M	%M	%M	**%M**	e.s.d. mol%	**%M**	e.s.d. mol%	**%M**	e.s.d. mol%	**%M**	e.s.d. mol%		
**0**	**50.0**	**50.0**	**0.0**	**50.0**		**50.0**		**0.0**		**0.0**			
**30**	47.9	48.0	**4.1**	**48.1**	0.6	**44.7**	0.5	**6.4**	0.8	**0.8**	0.4	*11.3*	*2.0*
**45**	47.4	48.2	**4.4**	**48.7**	0.6	**45.3**	0.6	**4.9**	0.8	**1.1**	0.4	*11.5*	*2.0*
**50**	29.5	30.1	**40.5**	**27.9**	0.4	**25.9**	0.3	**43.0**	0.5	**3.2**	0.7	*10.8*	*1.9*
**60**	31.8	32.4	**35.9**	**40.8**	0.5	**37.8**	0.5	**20.7**	0.6	**0.7**	0.4	*11.5*	*2.0*
**70**	20.1	20.3	**59.6**	**26.6**	0.5	**24.6**	0.4	**47.4**	0.6	**1.3**	0.4	*9.3*	*1.7*
**80**	12.4	12.4	**75.1**	**15.8**	0.4	**14.7**	0.4	**67.4**	0.5	**2.1**	0.4	*9.4*	*1.6*
**90**	1.9	1.9	**96.2**	**0.6**	0.3	**0.6**	0.2	**95.4**	0.5	**3.4**	0.4	*9.5*	*1.7*
**100**	1.2	1.5	**97.3**	**1.1**	0.4	**1.0**	0.4	**95.6**	0.6	**2.2**	0.3	*7.0*	*1.8*
**120**	3.8	3.8	**92.4**	**3.3**	0.3	**3.0**	0.3	**90.6**	0.6	**3.1**	0.4	*9.1*	*1.6*
**150**	0.5	0.3	**99.2**	**2.3**	0.5	**2.1**	0.5	**94.3**	0.8	**1.3**	0.4	*12.2*	*2.3*
**300**	1.0	1.2	**97.8**	**1.1**	0.4	**1.1**	0.4	**94.2**	0.7	**3.6**	0.4	*9.7*	*1.7*
**24h**	0.9	1.1	**98.0**	**0.5**	0.3	**0.4**	0.3	**95.1**	0.6	**4.0**	0.4	*9.7*	*1.8*

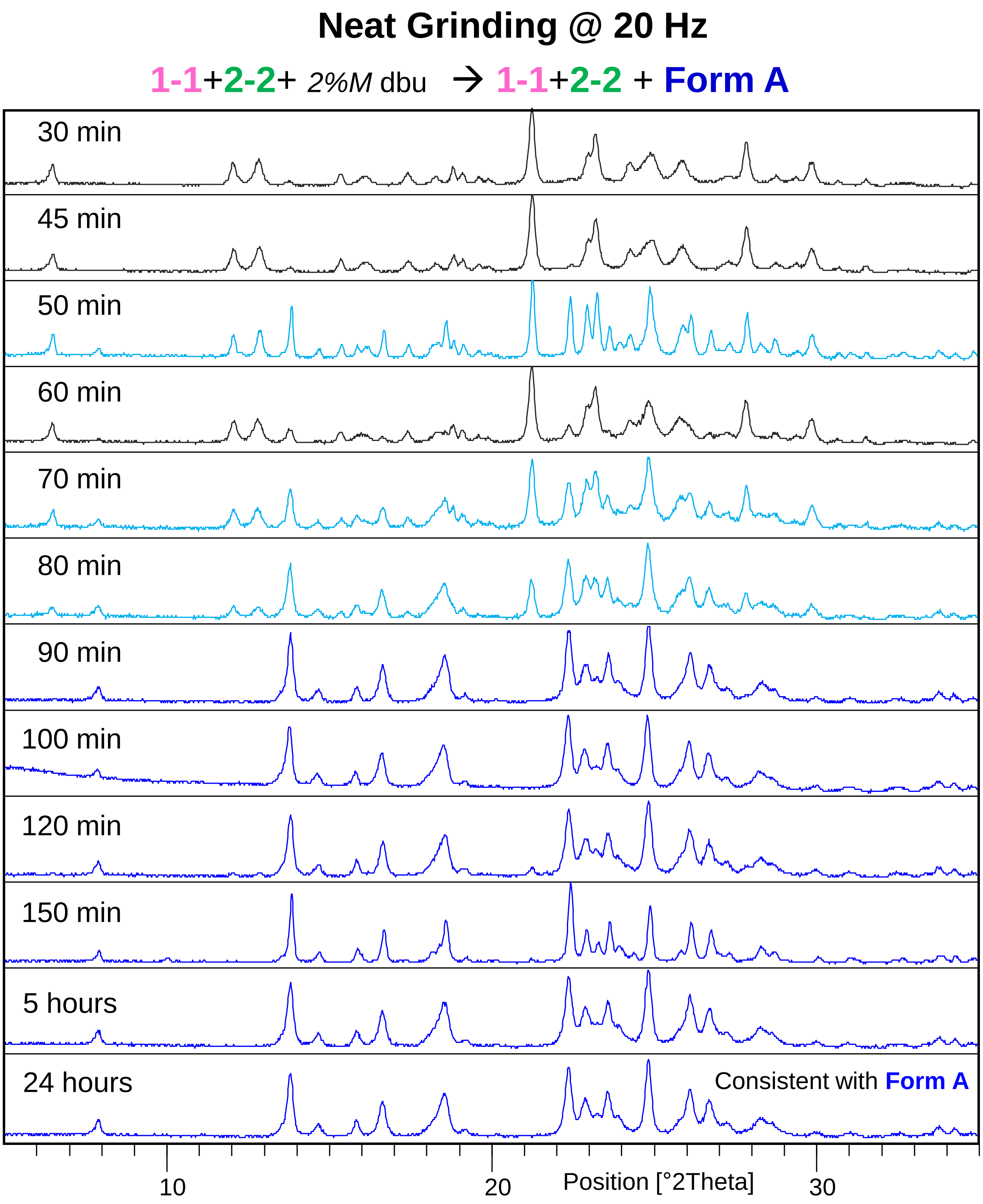

Figure S 31 PXRD scans of the reaction product of the kinetic study of a ball mill NG reaction at 20 Hz. Each PXRD scan was obtained from a single point experiment. The PXRD sample was prepared immediately after completion of the grinding experiment, and scanned between 5 and 45º (2θ). Typical PXRD scans are shown on Figure S 6.

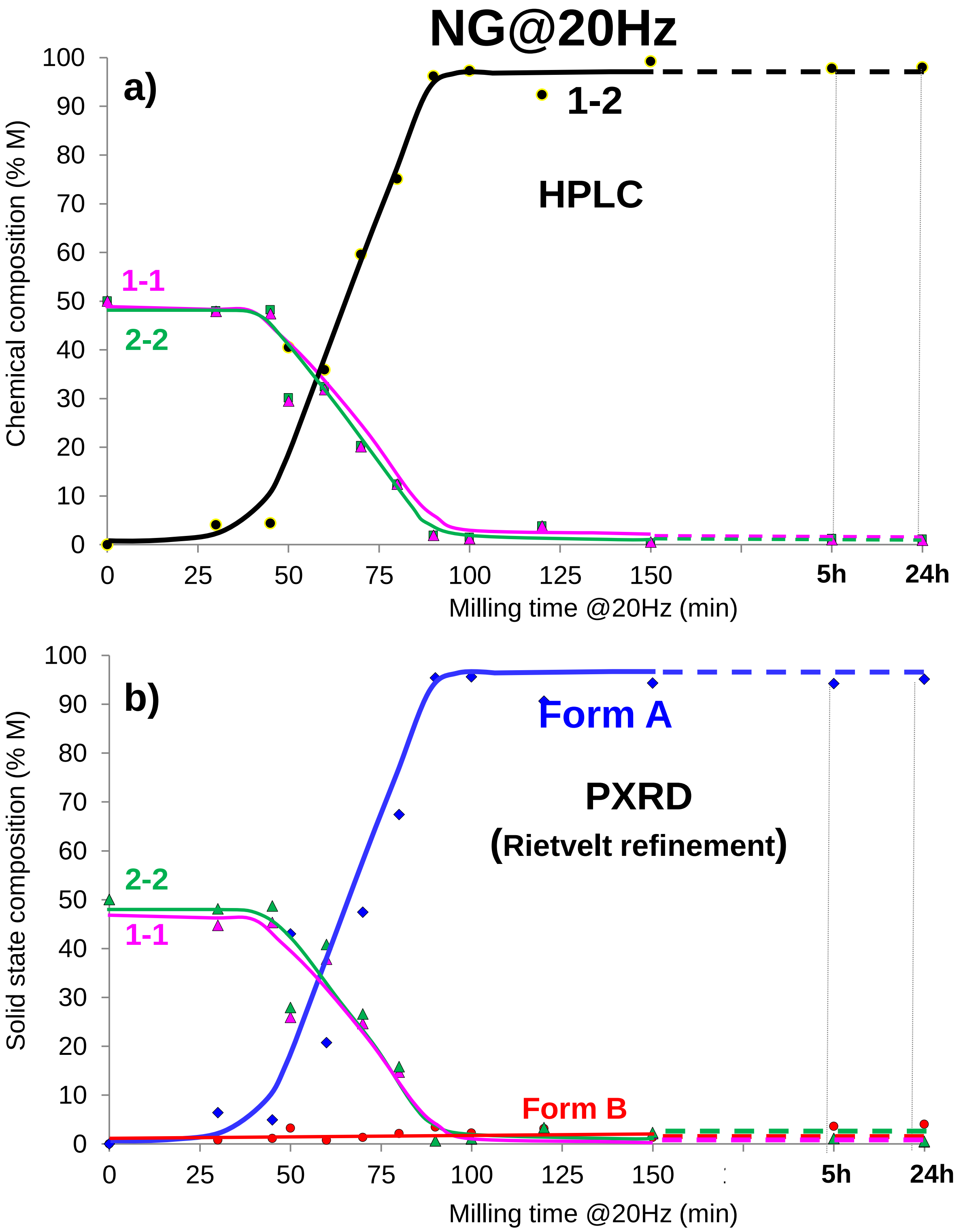

Figure S 32: Kinetic plots of the solid-state DCC ball mill NG reaction at 20 Hz of **1-1** and **2-2** in the presence of 2 mol % dbu leading to thermodynamic equilibrium and quantitative formation of **Form A**. a) chemical composition determined by HPLC; b) solid-state composition calculated by the Rietveld refinement from PXRD data.

Table S 23 Crystal Scherrer size of the starting materials (**1-1** and **2-2**) and the product **Form A** in relation to the chemical and solid-state composition of each single point experiment of the ball mill NG reaction at 20Hz.

Kinetics of ball mill NG grinding reaction @20Hz															
0.68 mmol DCC ([1-1]+[2-2] + 2%dbu															
grinding time @ 20Hz	HPLC			Rietveld refinement				Scherrer size							
	1-1	2-2	1-2	1-1	2-2	Form A	Form B	1-1		2-2		Form A		Form B	
min	%M	%M	%M	%M	%M	%M	%M	nm	e.s.d. nm	nm	e.s.d. nm	nm	e.s.d. nm	nm	e.s.d. nm
**30**	47.9	48.0	**4.1**	**48.1**	**44.7**	**6.4**	**0.8**	41	*1*	63	*1*				
**45**	47.4	48.2	**4.4**	**48.7**	**45.3**	**4.9**	**1.1**	39	*1*	58	*1*				
**50**	29.5	30.1	**40.5**	**27.9**	**25.9**	**43.0**	**3.2**	74	*3*	130	*5*	186	*8*		
**60**	31.8	32.4	**35.9**	**40.8**	**37.8**	**20.7**	**0.7**	34	*1*	56	*1*	52	*3*		
**70**	20.1	20.3	**59.6**	**26.6**	**24.6**	**47.4**	**1.3**	36	*1*	57	*2*	55	*2*		
**80**	12.4	12.4	**75.1**	**15.8**	**14.7**	**67.4**	**2.1**	38	*2*	58	*3*	51	*1*		
**90**	1.9	1.9	**96.2**	**0.6**	**0.6**	**95.4**	**3.4**					51	*1*		
**100**	1.2	1.5	**97.3**	**1.1**	**1.0**	**95.6**	**2.2**								
**120**	3.8	3.8	**92.4**	**3.3**	**3.0**	**90.6**	**3.1**					48	*1*		
**150**	0.5	0.3	**99.2**	**2.3**	**2.1**	**94.3**	**1.3**								
**5h**	1.0	1.2	**97.8**	**1.1**	**1.1**	**94.2**	**3.6**					45	*1*		
**24h**	0.9	1.1	**98.0**	**0.5**	**0.4**	**95.1**	**4.0**								

Scherrer size calculation is only reliable for contents > 20%M, must be in range 30 nm-350 nm and the size > 3 x esd

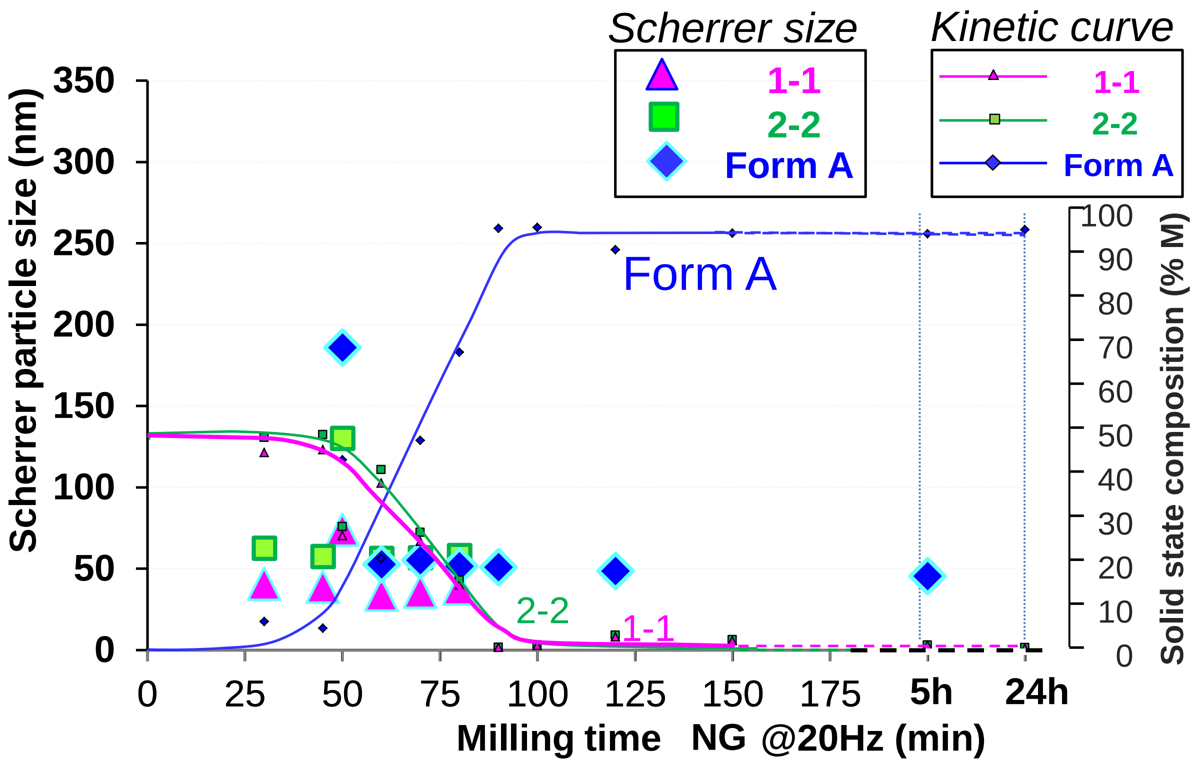

Figure S33 Scherrer size (nm) for the homodimers **1-1**, **2-2** and the reaction product **Form A** determined at different grinding times of the experimental kinetic stiudy at 20 Hz . **Form B** is not formed by NG experiments. Scherrer size determination is only reliable for compounds > 20 mol % and their estimated standard deviations (esd) are much smaller than the symbols used. Y-axis on the right is expressed as mol % composition of **1-1**, **2-2** and **Form A**.

6.4 Kinetic NG studies of 1-1 and 2-2 with 2 mol % dbu at 15 Hz

This section presents the characterization of the reaction product of the kinetic ball mill NG studies at 15 Hz using single point experiments. The solid-state DCC reaction was performed following the procedure described in Section 3.1. The solid-state DCC reaction was run at different grinding times ranging from 5 min up to 24 h to investigate the kinetic profile leading to thermodynamic equilibrium. The chemical composition of the reaction product was determined by HPLC (Table S24 and Figure S35a). The phase composition of the reaction product was calculated from the Rietveld refinement of the PXRD scans (Table S25 and Figure S35b). The crystal size of **1-1**, **2-2** and **Form A** in the product was calculated using the Scherrer calculation applied to the Rietveld refinement (Table S26 and Figure S36). The PXRD scans are displayed on Figure S34.

Table S 24 Experimentals details of single point grinding experiments of solid-state DCC reaction of **1-1** and **2-2** performed in the presence of 2 mol % **dbu** under ball mill NG conditions at 15 Hz. Experiments are run from 5 min up to 24 h. The chemical composition of reaction products is determined by HPLC analysis. The HPLC conditions are shown below. This data is plotted on Figure S35a.

Kinetics of ball mill **NG** reaction @**15Hz**

[**1-1**]+[**2-2**]+2%M dbu

Reagents:	**$(2NO_2PhS)_2$**	**$(4ClPhS)_2$**	**HPLC conditions** [λ=259(8nm) ref 550(100nm)]		
MW:	308.33	287.23	Zorbax SB C18, 1.8 μm; 4.6mm ID x50 mm		
%M:	50%M	50%M	**A**: H_2O+0.1% HCOOH; **B**: MeCN+0.1%HCOOH		
mmol:	0.34 mmol	0.34 mmol	0-2 min 75-85%B; 2ml/min; 60°C; run time=2.0 min		
mgs:	104.38 mg	97.63 mg	**HPLC results**		
grinding time @ 15 Hz	$(2NO_2PhS)_2$ weighed	$(4ClPhS)_2$ weighed	$(2NO_2PhS)_2$ (**1-1**)	Product (**1-2**)	$(4ClPhS)_2$ (**2-2**)
min	mg	mg	%M	%M	%M
**0**	Expected %M from weighing		50.0	0.0	50.0
**60**	104.79	97.70	49.8	**0.5**	49.7
**90**	104.79	97.64	48.9	**0.8**	50.3
**120**	104.76	97.70	41.7	**15.7**	42.6
**120**	104.80	97.66	49.5	**1.2**	49.3
**180**	104.78	97.69	45.3	**8.8**	45.9
**195**	104.84	97.71	4.3	**92.1**	3.5
**210**	104.81	97.67	6.6	**87.6**	5.8
**225**	104.81	97.63	11.9	**76.6**	11.5
**240**	104.79	97.69	20.2	**59.6**	20.3
**275**	104.81	97.67	3.3	**93.3**	3.3
**300**	104.82	97.63	6.6	**87.5**	6.0
**360**	104.85	97.66	2.4	**95.6**	2.0
**420**	104.80	97.68	9.7	**80.6**	9.7
**480**	104.82	97.66	3.8	**92.4**	3,8
**540**	104.82	97.69	1.5	**96.9**	1.6
**600**	104.81	97.61	8.5	**82.6**	8.9
**24h**	104.83	97.62	2.2	**95.8**	2.0

Table S 25 Solid-state composition of the reaction products calculated using Rietveld refimenment from PXRD scans of single point experiments of the solid-state DCC reaction of **1-1** and **2-2** performed in the presence of 2 mol % **dbu** under ball mill NG conditions at 15 Hz. Experiments are run from 5 min up to 24 h. This data is plotted on Figure S35b. The PXRD scans used for the Rietveld refinement are shown on Figure S34.

Kinetics of ball mill **NG** reaction @**15Hz**															
0.68 mmol DCC ([**1-1**]+[**2-2**] + 2%dbu															
**grinding time @ 15Hz**	**HPLC**			**PXRD: Rietveld refinement**											
	**1-1**	**2-2**	**1-2**	**1-1**		**2-2**		**Form A**		**Form B**		Rwp Rietveld refinement Goodness		chisq Rietveld refinement Fit Index	
(min)	%M	%M	%M	**%M**	e.s.d. mol%	**%M**	e.s.d. mol%	**%M**	e.s.d. mol%	**%M**	e.s.d. mol%				
**0**	50.0	50.0	**0.0**	**50.0**		**50.0**		**0.0**		**0.0**					
**60**	49.8	49.7	**0.5**	**47.4**	0.5	**44.2**	0.5	**4.6**	0.6	**3.8**	0.5	*16.0*		*2.9*	
**90**	48.9	50.3	**0.8**	**47.6**	0.5	**44.4**	0.5	**4.7**	0.6	**3.3**	0.7	*16.2*		*2.9*	
**120**	41.7	42.6	**15.7**	**45.2**	0.6	**42.0**	0.6	**12.5**	0.8	**0.3**	0.5	*12.2*		*2.2*	
**120**	49.5	49.3	**1.2**	**47.2**	0.5	**44.0**	0.5	**5.3**	0.7	**3.4**	0.5	*13.9*		*2.5*	
**180**	45.3	45.9	**8.8**	**45.4**	0.6	**42.2**	0.6	**9.3**	0.9	**3.1**	0.6	*12.6*		*2.2*	
**195**	4.3	3.5	**92.1**	**3.6**	0.3	**3.3**	0.3	**89.7**	0.5	**3.3**	0.4	*8.8*		*1.6*	
**210**	6.6	5.8	**87.6**	**5.4**	0.3	**5.1**	0.3	**86.3**	0.5	**3.2**	0.4	*9.3*		*1.6*	
**225**	11.9	11.5	**76.6**	**13.1**	0.3	**12.2**	0.3	**72.6**	0.5	**2.1**	0.3	*9.3*		*1.6*	
**240**	20.2	20.3	**59.6**	**19.3**	0.4	**18.0**	0.4	**60.8**	0.6	**2.0**	0.4	*10.5*		*1.9*	
**275**	3.3	3.3	**93.3**	**7.6**	0.3	**7.1**	0.3	**82.5**	0.5	**2.7**	0.4	*8.6*		*1.5*	
**5h**	6.6	6.0	**87.5**	**6.3**	0.6	**5.9**	0.6	**84.8**	0.9	**3.0**	0.5	*11.3*		*1.9*	
**6h**	2.4	2.0	**95.6**	**1.9**	0.5	**1.7**	0.5	**93.2**	0.8	**3.2**	0.5	*9.7*		*1.7*	
**7h**	9.7	9.7	**80.6**	**9.8**	0.4	**9.1**	0.4	**78.7**	0.7	**2.4**	0.5	*9.7*		*1.7*	
**8h**	3.8	3,8	**92.4**	**2.8**	0.4	**2.6**	0.3	**92.0**	0.6	**2.6**	0.4	*10.1*		*1.8*	
**9h**	1.5	1.6	**96.9**	**0.5**	0.2	**0.5**	0.2	**95.6**	0.4	**3.4**	0.3	*9.8*		*1.7*	
**10h**	8.5	8.9	**82.6**	**8.5**	0.4	**7.9**	0.3	**81.8**	0.5	**1.8**	0.4	*8.7*		*1.6*	
**24h**	2.2	2.0	**95.8**	**1.4**	0.3	**1.3**	0.3	**94.4**	0.6	**2.8**	0.4	*10.3*		*1.8*	

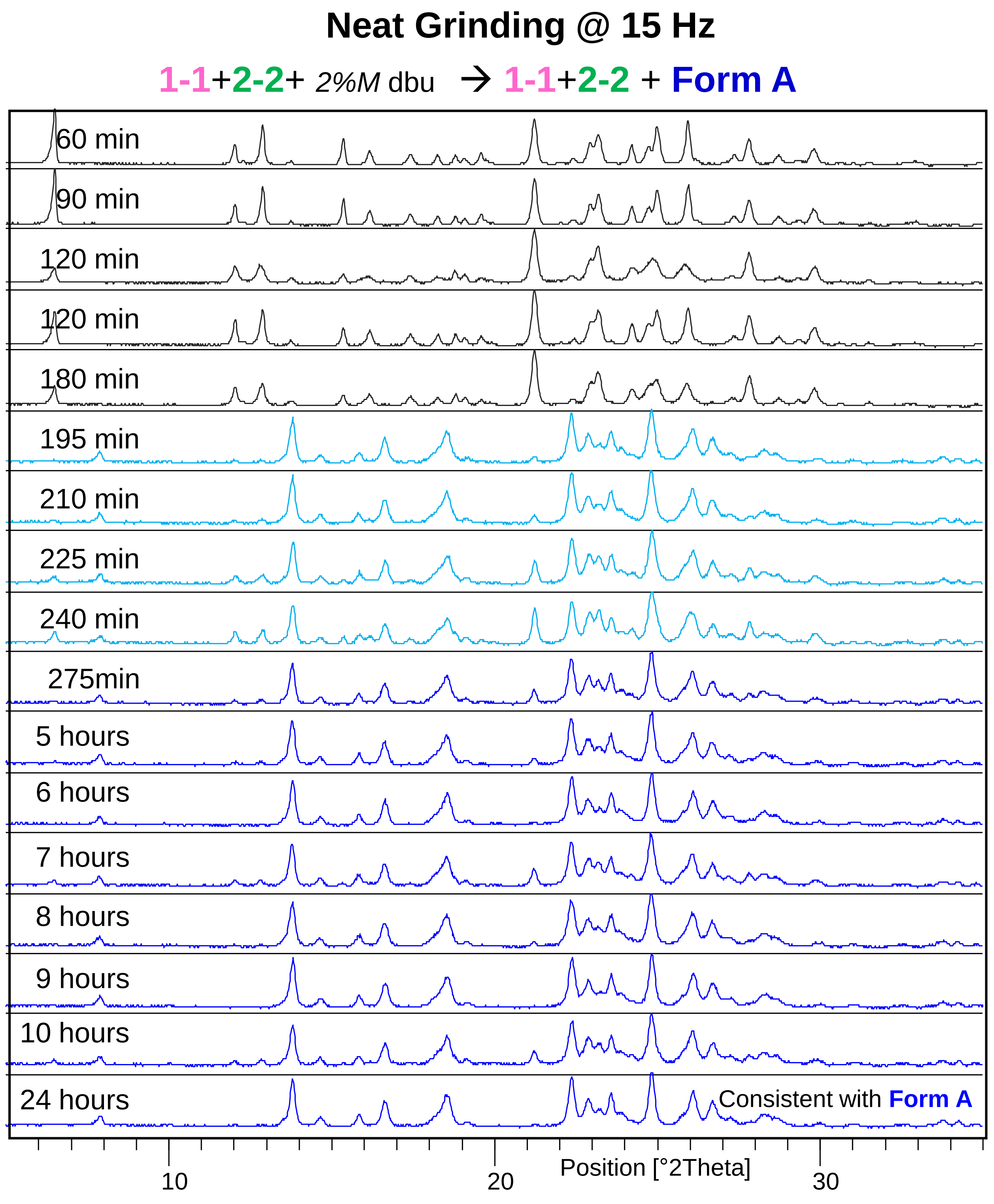

Figure S 34 PXRD scans of the reaction product of the kinetic study of a ball mill NG reaction at 15 Hz. Each PXRD scan was obtained from a single point experiment. The PXRD sample was prepared immediately after completion of the grinding experiment, and scanned between 5 and 45° (2θ). Typical PXRD scans are shown on Figure S 6.

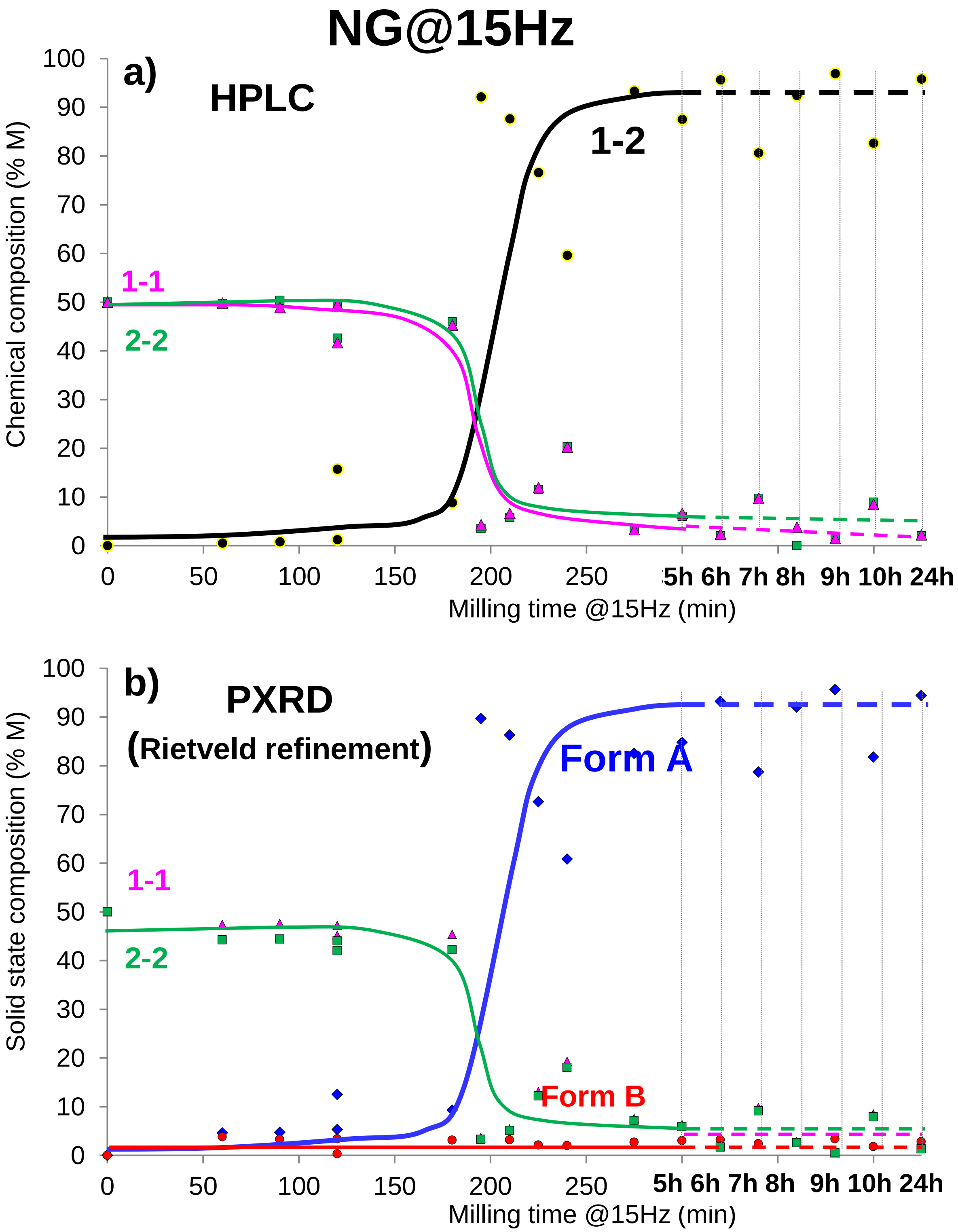

Figure S 35 Kinetic plots of the solid-state DCC ball mill NG reaction at 15 Hz of **1-1** and **2-2** in the presence of 2 mol % **dbu** leading to thermodynamic equilibrium and quantitative formation of **Form A**. a) chemical composition determined by HPLC; b) solid-state composition calculated by the Rietveld refinement from PXRD data.

Table S 26 Crystal Scherrer size of the starting materials (**1-1** and **2-2**) and the product **Form A** in relation to the chemical and solid-state composition of each single point experiment of the ball mill NG reaction at 15Hz.

Kinetics of ball mill **NG** reaction @**15Hz**															
0.68 mmol DCC ([**1-1**]+[**2-2**] + 2%M dbu															
**grinding time @ 15Hz**	**HPLC**			**Rietveld refinement**				**Scherrer size**							
	**1-1**	**2-2**	**1-2**	**1-1**	**2-2**	**Form A**	**Form B**	**1-1**		**2-2**		**Form A**		**Form B**	
min	%M	%M	%M	%M	%M	%M	%M	**nm**	e.s.d. nm	**nm**	e.s.d. nm	**nm**	e.s.d. nm	**nm**	e.s.d. nm
**60**	49.8	49.7	**0.5**	47.4	44.2	4.6	3.8	240	*13*	75	*2*				
**90**	48.9	50.3	**0.8**	47.6	44.4	4.7	3.3	235	*13*	86	*2*				
**120**	41.7	42.6	**15.7**	45.2	42.0	12.5	0.3	39	*1*	56	*1*				
**120**	49.5	49.3	**1.2**	47.2	44.0	5.3	3.4	113	*4*	69	*2*				
**180**	45.3	45.9	**8.8**	45.4	42.2	9.3	3.1	61	*2*	66	*2*				
**195**	4.3	3.5	**92.1**	3.6	3.3	89.7	3.3			79	*14*	**51**	*1*		
**210**	6.6	5.8	**87.6**	5.4	5.1	86.3	3.2			61	*7*				
**225**	11.9	11.5	**76.6**	13.1	12.2	72.6	2.1	58	*4*	64	*3*	**55**	*1*		
**240**	20.2	20.3	**59.6**	19.3	18.0	60.8	2.0	75	*5*	62	*3*	**54**	*1*		
**275**	3.3	3.3	**93.3**	7.6	7.1	82.5	2.7					**55**	*1*		
**5h**	6.6	6.0	**87.5**	6.3	5.9	84.8	3.0					**51**	*1*		
**6h**	2.4	2.0	**95.6**	1.9	1.7	93.2	3.2					**54**	*1*		
**7h**	9.7	9.7	**80.6**	9.8	9.1	78.7	2.4								
**8h**	3.8	3,8	**92.4**	2.8	2.6	92.0	2.6					**46**	*1*		
**9h**	1.5	1.6	**96.9**	0.5	0.5	95.6	3.4					**51**	*1*		
**10h**	8.5	8.9	**82.6**	8.5	7.9	81.8	1.8					**54**	*1*		
**24h**	2.2	2.0	**95.8**	1.4	1.3	94.4	2.8					**56**	*1*		
Scherrer size calculation is only reliable for contents > 20%M, must be in range 30 nm-350 nm and the size > 3 x esd															

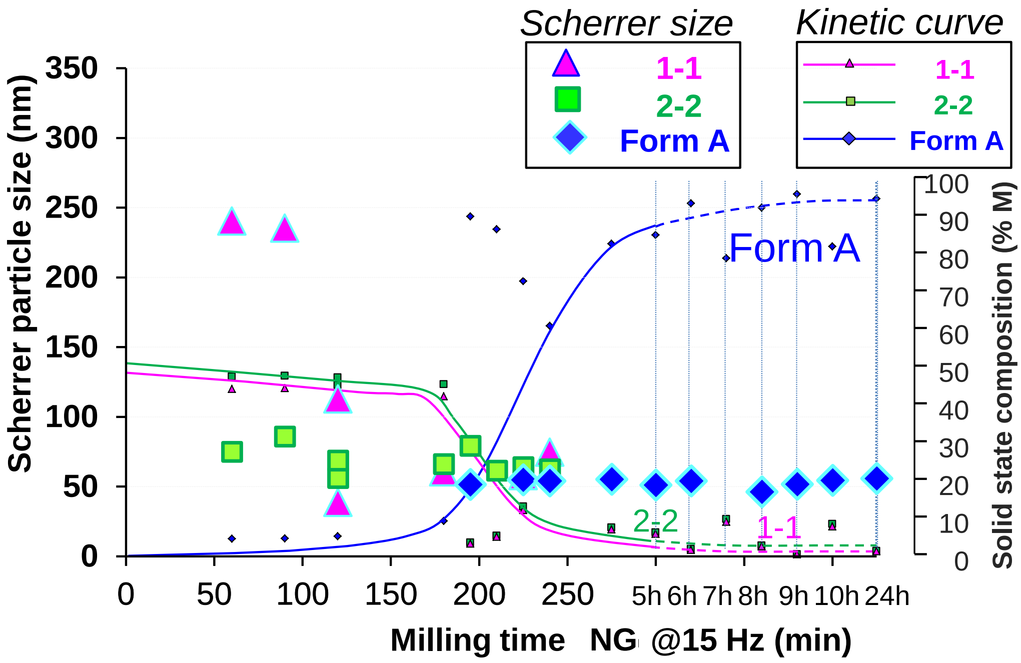

Figure S36 Scherrer size (nm) for the homodimers **1-1**, **2-2** and the reaction product **Form A** determined at different grinding times of the experimental kinetic stiudy at 15 Hz . **Form B** is not formed by NG experiments. Scherrer size determination is only reliable for compounds > 20 mol % and their estimated standard deviations (esd) are much smaller than the symbols used. Y-axis on the right is expressed as mol % composition of **1-1**, **2-2** and **Form A**.

6.5 *Proof of homogeneity in NG at low grinding frequency (15 Hz)*

Table S 27 Investigation of the homogeneity of the chemical composition of the powder in the grinding jar after ball mill NG at 15 Hz over 225 min and 275 min. Less than 1 mg of powder was required for HPLC analysis and therefore a microspatula was used to obtain the powder from the desired areas in the jar or ball bearings.

Kinetics of ball mill **NG** reaction @**15Hz- Chemical composition**						
[**1-1**]+[**2-2**]+2%M dbu + 50µL MeCN						
grinding time @ 15 Hz	Position inside jar	replicates in same part of jar	$(2NO_2PhS)_2$ (**1-1**)	Product (**1-2**)	$(4ClPhS)_2$ (**2-2**)	Pictures of jar with powder opened just after compeltion of grinding
min			%M	%M	%M	
**225**	jar /male	1	13.4	**73.7**	12.9	
**225**	jar /male	2	11.9	**76.6**	11.5	
**225**	jar /male	3	12.0	**76.4**	11.6	no
**225**	jar /female	1	12.2	**76.1**	11.7	pictures available
**225**	jar /female	2	13.9	**72.7**	13.4	
**275**	jar /male	1	3.6	**92.7**	3.7	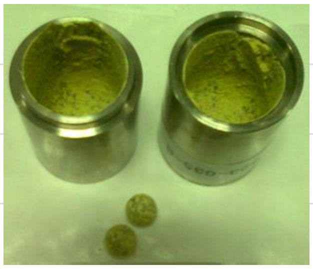
**275**	jar /male	2	3.3	**93.3**	3.3	
**275**	jar /female	1	3.3	**93.2**	3.5	
**275**	jar /female	2	3.4	**93.0**	3.5	

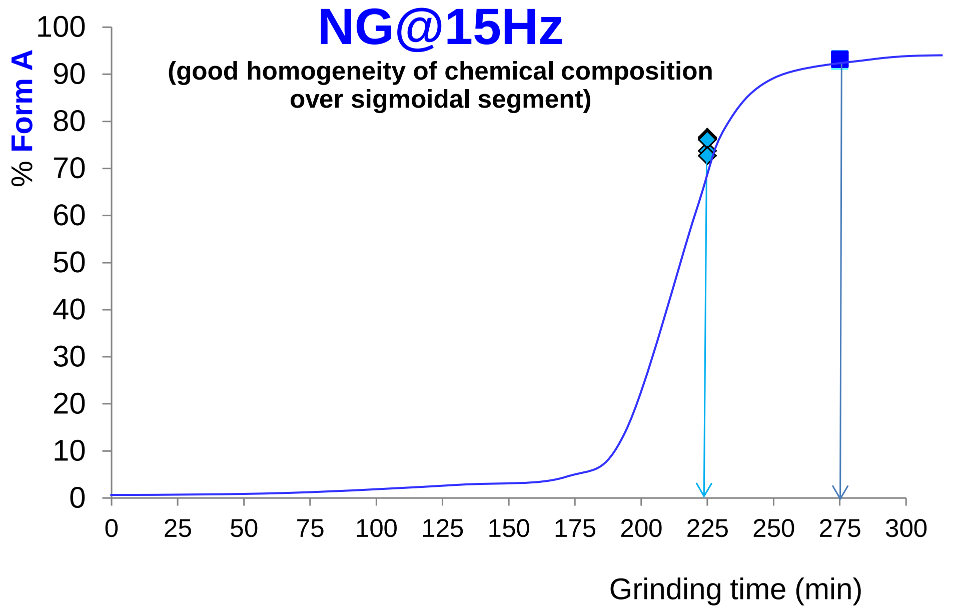

Figure S37 Plotting homogeneity of chemical composition as mol % of samples of powder taken from different positions in grinding jar and balls of the reaction product of the DCC reaction of of **1-1** and **2-2** performed in the presence of 2 mol % **dbu** under ball mill NG conditions at 15 Hz.

6.6 Comparison between kinetics NG studies at 30, 25, 20 and 15 Hz

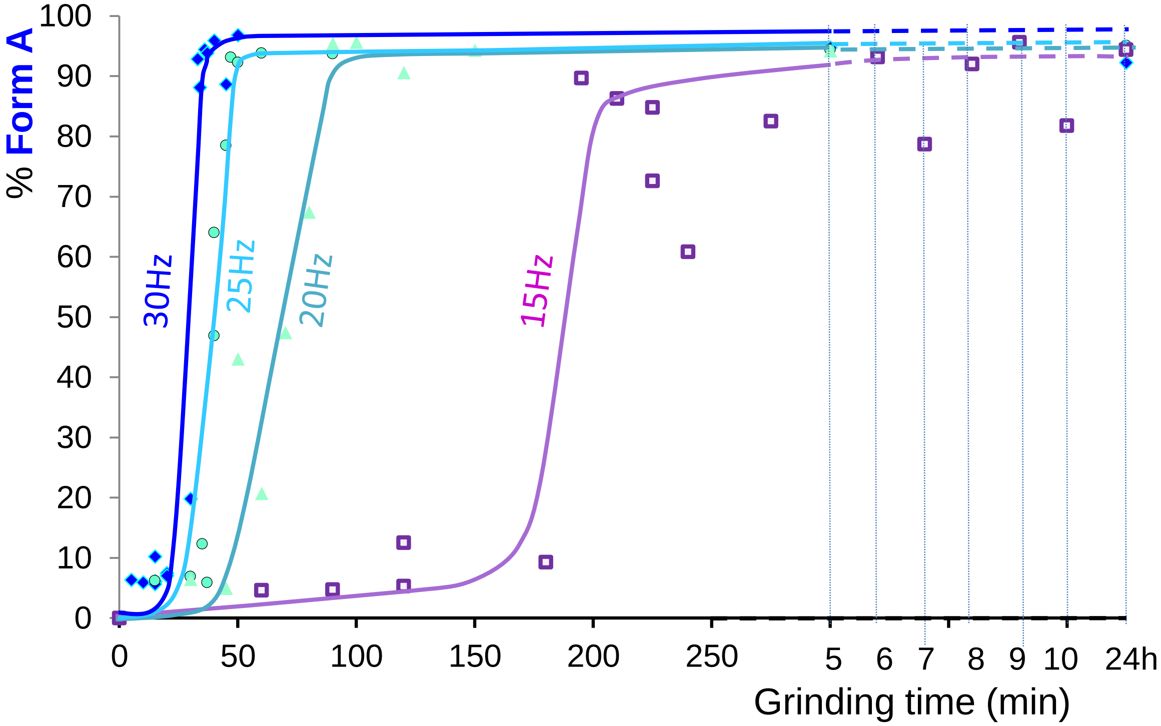

Figure S38 Comparison between the kinetic curves obtained from individual experiments ground between 5 min and 24 hours of the solid-state DCC reaction run under ball mill NG conditions at 30Hz, 25Hz, 20Hz and 15Hz. The curves are a guide to the eye only.

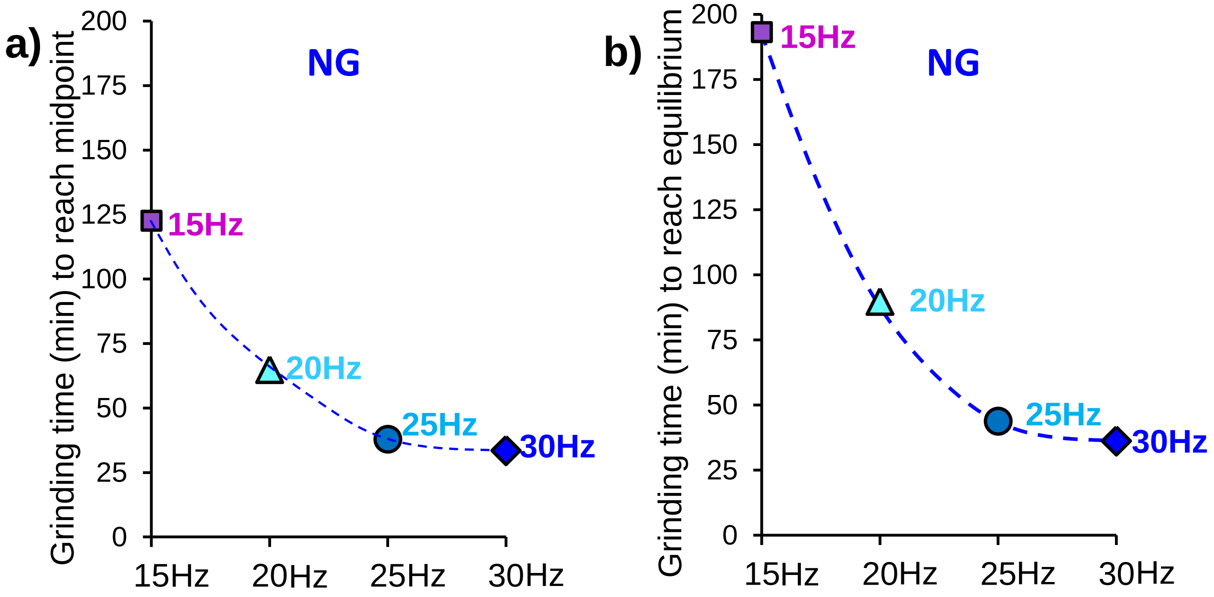

Figure S39 Correlation between the grinding time (min) and the frequency of ball mill NG reaction of **1-1** and **2-2** in presence of dbu for a) the midpoint of the sigmoidal curve and b) the start of the thermodynamic equilibrium of the kinetic curves obtained for ball mill NG experiments milled at 15 Hz, 20 Hz, 25 Hz and 30 Hz.

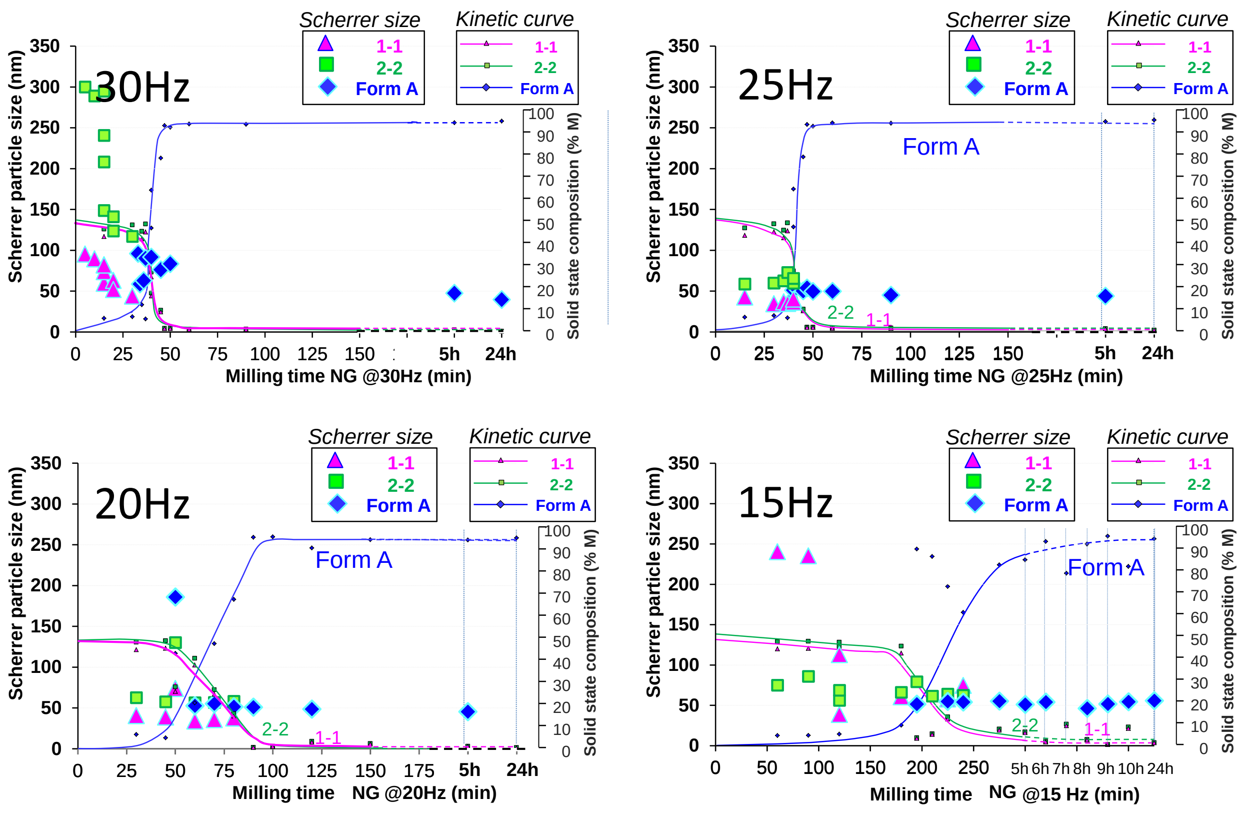

Figure S 40 Comparison of the Scherrer particle size of **1-1**, **2-2** and **Form A** in ball mill NG reactions performed at 30Hz, 25Hz, 20Hz and 15Hz.

7 Literature

1. Belenguer, A. M.; Friscic, T.; Day, G. M.; Sanders, J. K. M., Solid-state dynamic combinatorial chemistry: reversibility and thermodynamic product selection in covalent mechanosynthesis. *Chem. Sci.* **2011,** *2* (4), 696-700.
2. Belenguer, A. M.; Lampronti, G. I.; Wales, D. J.; Sanders, J. K. M., Direct Observation of Intermediates in a Thermodynamically Controlled Solid-State Dynamic Covalent Reaction. *J. Am. Chem. Soc.* **2014,** *136* (46), 16156-16166.
3. Belenguer, A. M.; Lampronti, G. I.; Sanders, J. K. M., Reliable Mechanochemistry: Protocols for Reproducible Outcomes of Neat and Liquid Assisted Ball-mill Grinding Experiments. *JoVE* **2018,** (131), e56824.
4. Cougnon, F. B. L.; Sanders, J. K. M., Evolution of Dynamic Combinatorial Chemistry. *Acc. Chem. Res.* **2011,** *45* (12), 2211-2221.
5. Black, S. P.; Sanders, J. K. M.; Stefankiewicz, A. R., Disulfide exchange: exposing supramolecular reactivity through dynamic covalent chemistry. *Chem. Soc. Rev.* **2014,** *43* (6), 1861-1872.
6. Herrmann, A., Dynamic combinatorial/covalent chemistry: a tool to read, generate and modulate the bioactivity of compounds and compound mixtures. *Chem. Soc. Rev.* **2014,** *43* (6), 1899-1933.
7. Jin, Y.; Yu, C.; Denman, R. J.; Zhang, W., Recent advances in dynamic covalent chemistry. *Chem. Soc. Rev.* **2013,** *42* (16), 6634-6654.
8. Corbett, P. T.; Leclaire, J.; Vial, L.; West, K. R.; Wietor, J.-L.; Sanders, J. K. M.; Otto, S., Dynamic Combinatorial Chemistry. *Chemical Reviews (Washington, DC, United States)* **2006,** *106* (9), 3652-3711.
9. Belenguer, A. M.; Lampronti, G. I.; Cruz-Cabeza, A. J.; Hunter, C. A.; Sanders, J. K. M., Solvation and surface effects on polymorph stabilities at the nanoscale. *Chem. Sci.* **2016,** *7* (11), 6617-6627.
10. Coelho, A., *TOPAS-Academic, Coelho Software, Brisbane, Australia* **2007**.
11. Payzant, E. A., *"Other Topics" in Principles and Applications of Powder Diffraction edited by Clearfield, A, Reibenspies, J. H. and Bhuvanesh N.* John Wiley and Sons, Ltd: 2008; p 365-380.
12. Black, D. R., Windover, D., Henins, A., Filliben, J., and Cline, J. P, Certification of standard reference material 660B. *Powder Diffraction* **2011,** *26* (02), 155-158.
13. R. W. Cheary, A. A. C., J. P. Cline, Fundamental Parameters Line Profile Fitting in Laboratory Diffractometers. *J Res Natl Inst Stand Technol* **2004,** *109* (1), 1–25.
