# Supplementary material for: Understanding the unexpected effect of frequency on the kinetics of a covalent reaction under ball-milling conditions
Source: Beilstein J Org Chem. 2019 Jun 5;15:1226–35. doi: 10.3762/bjoc.15.120 (PMC6604707; doi:10.3762/bjoc.15.120)
Supplement: File 2 — Kinetic model parameterization and additional model features. [file Beilstein_J_Org_Chem-15-1226-s002.pdf]

BEILSTEIN JOURNAL OF ORGANIC CHEMISTRY

Supporting Information

for

Understanding the unexpected effect of frequency on the kinetics of a covalent reaction under ball-milling conditions

Ana M. Belenguer, Adam A. L. Michalchuk, Giulio I. Lampronti and Jeremy K. M. Sanders

Beilstein J. Org. Chem. **2019,** *15,* 1226–1235. doi:10.3762/bjoc.15.120

Kinetic model parameterization and additional model features

License and Terms: This is a supporting information file under the terms of the Creative Commons Attribution License (http://creativecommons.org/licenses/by/4.0). Please note that the reuse, redistribution and reproduction in particular requires that the authors and source are credited.
The license is subject to the *Beilstein Journal of Organic Chemistry* terms and conditions: (https://www.beilstein-journals.org/bjoc)

TABLE OF CONTENTS

S1 Overview of Equations Presented in Main Text………………………………………. S2

S2 Effect of Modifying E_{act}: τ ………………………………………………………………. S3

S3 Conceptualisation of Constant Rate of Energy Accumulation ………………….. S5

S4 Scaling Energy Accumulation Rate …………………………………………………….. S6

S5 Effect of Mixing ……………………………………………………………………………………. S10

S6 References …………………………………………………………………………………………….S11

Nomenclature and abbreviations used

ν	frequency of milling
W	ideal impact energy
τ	relaxation rates
E_o	activation energy
E_{acc}	accumulated energy
k_1	rate constant associated with mixing
$k_1{}'$	rate constant associated with de-mixing
k_2	rate constant associated with mechanochemical transformation

S1 Overview of Equations Presented in Main Text

To assist in the reading of this document, we summarise here the relevant equations presented in the manuscript. For their complete discussion, readers are referred to the main text. Note that Equation numbering is maintained from the manuscript for clarity.

The kinetic equations in this manuscript are based on the simplified reaction scheme,

$$A + B \underset{k_1'}{\overset{k_1}{\rightleftharpoons}} [AB] \overset{k_2}{\rightarrow} C$$

Here, k_1 and $k_1{}'$ are taken to be the mixing and demixing rate constants, associated with formation of heterogeneous contacts during milling. The mechanochemical transformation is dictated by rate constant,

$$k_2 = A.exp\left(-\frac{E_0 - E_{acc}}{W}\right) \qquad \textbf{Equation 2}$$

Where A is a pre-exponential factor, E_o is the activation energy, W is the ideal impact energy and E_{acc} is accumulated energy given by,

$$E_{acc} = \begin{cases} W(\nu - \tau);\ \nu/\tau\ \geq\ 1 \\ 0\ ;\ \nu/\tau < 1 \end{cases} \qquad \textbf{Equation 3}$$

Formation of kinetics in this way allows for consideration of the non-equilibrium energy conditions experienced by particles under ball milling conditions. Furthermore, this allows energy accumulation of successive impulses, and hence allows chemical reactions whose activation energies would be otherwise too high to occur by the small amounts of energies associated with individual impacts. Note that Equation 3 assumes a linear increase in the accumulated energy.

S2 Effect of Modifying E_o: τ

The accumulation of energy depends intimately on the rate of energy accumulation E_{acc} and thus the frequency of milling ν and relaxation rates τ.[1] Furthermore, in line with established formalisms in chemical and physical reactivity, a particular threshold (activation energy, E_o) must be achieved. Of these three parameters, both τ and E_o must be fit, as they are not known experimentally. From Equation 2 in Section S1, this leads to the important ratio, E_o: τ. The values of τ which lead to reaction are limited by the milling frequency (Equation 3). This is exemplified by consideration of three values of τ for 15 Hz milling, Figure S1

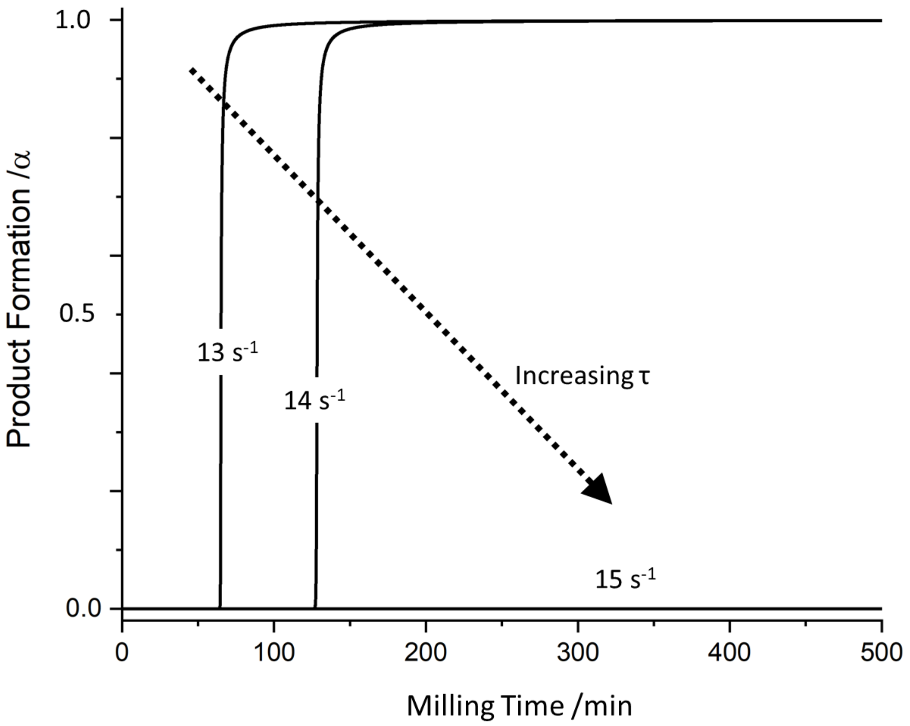

Figure S1: Effect of increasing τ (and hence decreasing E_o: τ ratio) for 15 Hz ball mill neat milling model. For each curve, the value of E_o is held constant, and τ is increased from 13 s^{-1}, 14 s^{-1} and 15 s^{-1}. Note that when relaxation is too rapid (here τ=15 s^{-1}), energy does not accumulate and no reaction is observed. This is seen with the flat line with product formation.

Due to the restriction imposed by the 15 Hz milling model, a ratio of E_o: τ was selected so as to best reproduce the 15 Hz data. This same value $E_0 : \tau = 17.48$, was used throughout all modelling work, unless otherwise stated. Importantly, the choice of E_o: τ affects only the relative onset times for each curve, but has no effect on the overall structure of the kinetic model, Figure S2.

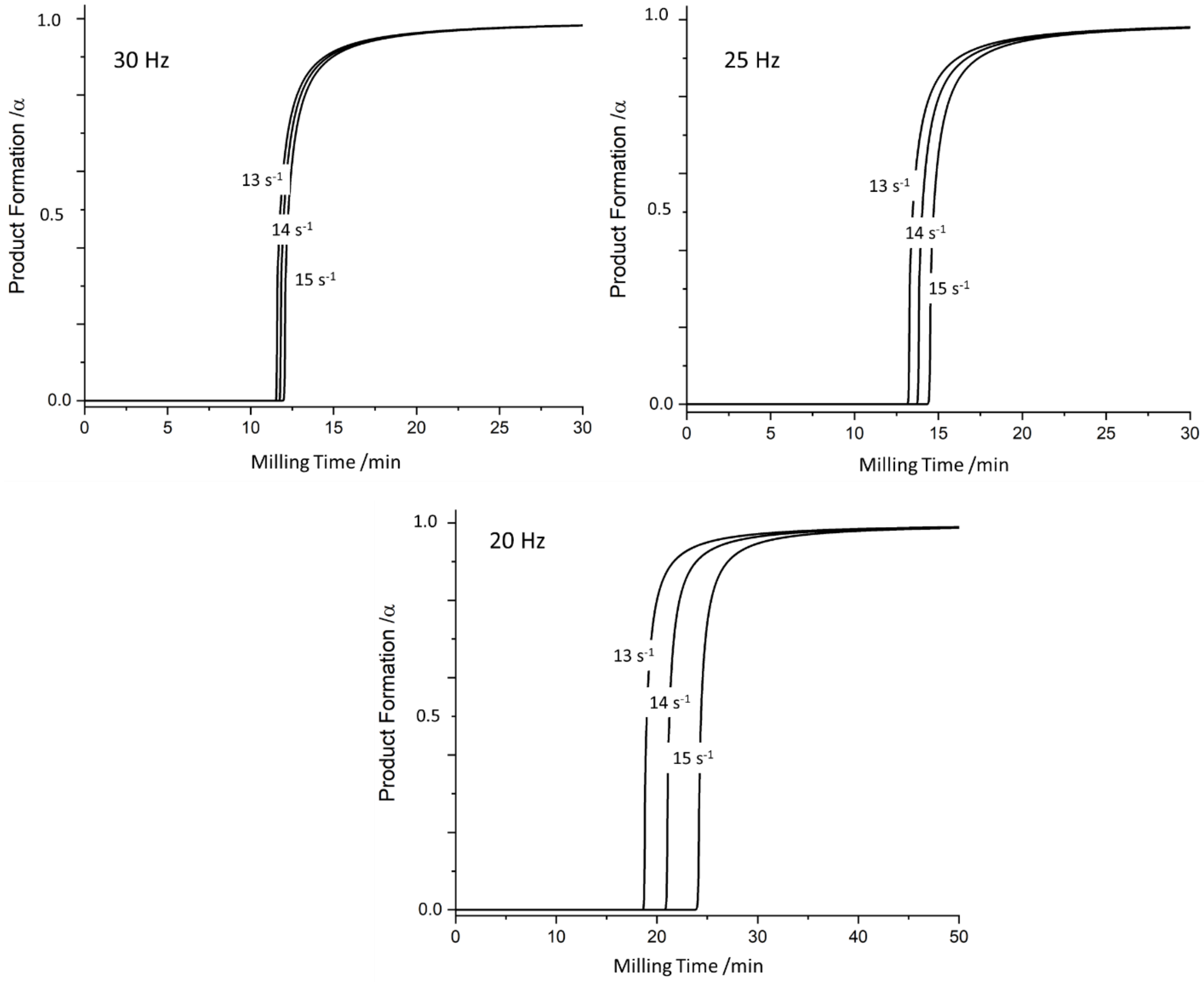

Figure S2: Effect of E_o: τ on the kinetic models for 30 Hz, 25 Hz and 20 Hz. In each case E_o is held constant, and the value of τ is increased (13 s^{-1}, 14 s^{-1}, 15 s^{-1}) leads to a longer milling time before onset of the transformation.

S3 Conceptualisation of Constant Rate of Energy Accumulation

The approximation applied in Equation 3 is most readily conceptualised schematically. We adopt a simple example in which the rate of energy relaxation is 1 Hz, and impact frequency 3 Hz, Scheme S1. With linear accumulation of energy (as assumed in this work), the total energy accumulated per second is constant, and depends on the relative ratio of frequency and energy relaxation. The same (constant increase) is observed if energy relaxation is assumed to be non-linear, although the analytical form describing this accumulation differs. However, the exact form of energy accumulation is of little importance without explicit knowledge of τ.

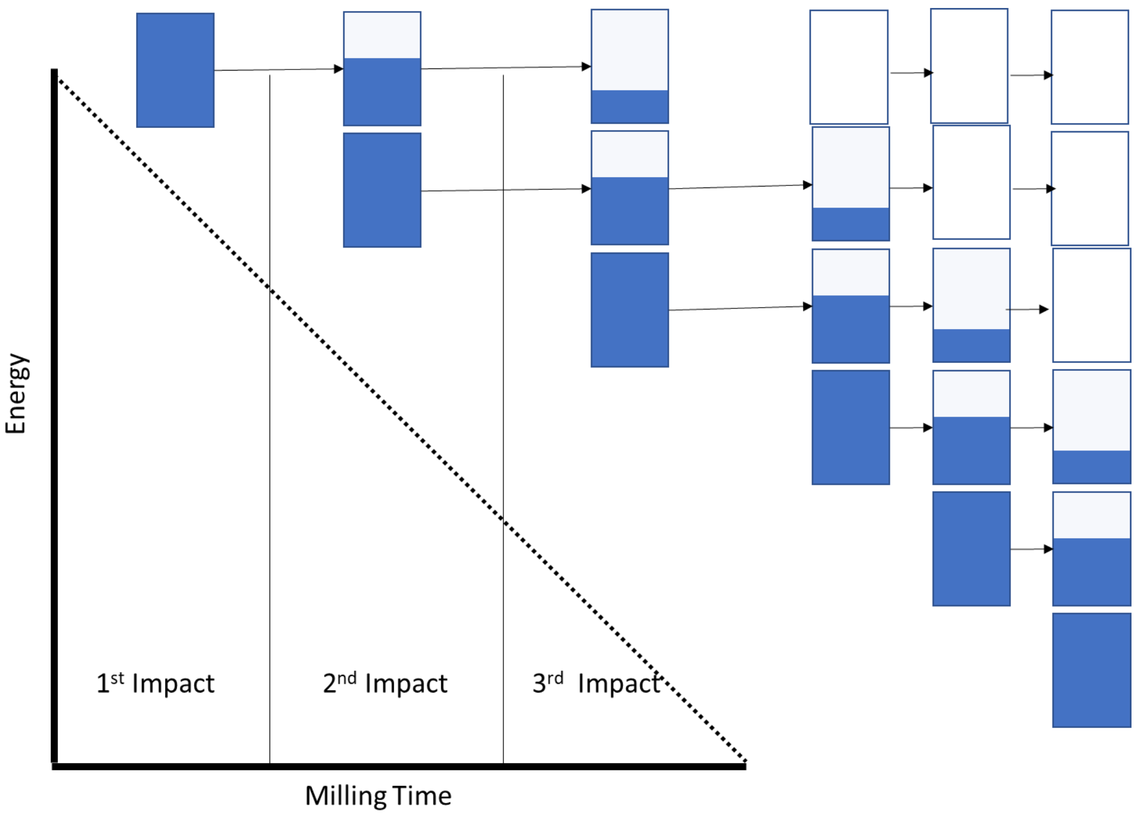

Scheme S1: Schematic representation of the accumulation of energy per impact with time. Impact energy are given as blue rectangles. Fill level is indicative of the approximate remaining energy of the impact. Arrows are used to follow an impact as a function of grinding time. The schematic *x-y* plot highlights the rate of energy relaxation of an impulse per unit time, and corresponds to the remaining energy of the initial impact.

S4 Scaling Energy Accumulation Rate

The model curves shown in the main text, described by Equation 3, assume a linear increase in the rate of mechanical energy. Without explicit knowledge of nature of energy accumulation in mechanochemical systems, however, it is not possible to derive a more accurate description of this process. As noted in the text, linear accumulation is used as a simplification for this proof of concept study. We can assume, however, that defect formation will become somewhat more difficult with increased stored energy. Hence, the distribution of input energy between work and heat is likely not constant. With the caveat of introducing yet more major assumptions, we briefly here explore the qualitative effect of introducing this non-linearity into the kinetic equation, Equation 3. Without knowledge of defect energy, we base scaled accumulation as a function of the activation energy of the system.

As an explicative example, we scale the accumulated energy E_{acc}at time t as a function of its current value relative to the activation energy E_0,

$$E_{acc}(t) = E_{acc} + W\big(1 - (E_{acc}/E_0)\big)$$ **Equation S1**

Where W is the impact energy. If this modified form of Equation 3 is employed, energy accumulation occurs according to Figure S3A. In this way, energy accumulation decreases asymptotically towards the activation energy of the system. Importantly, the spontaneous onset of the kinetic curve, Figure S3B, is maintained. An important structural feature associated with Equation S1 is the gentler sigmoidal nature of the curve (*i.e.* more gradual onset / plateau), Figure S4. This is more consistent with experimental trends displayed in the main text.

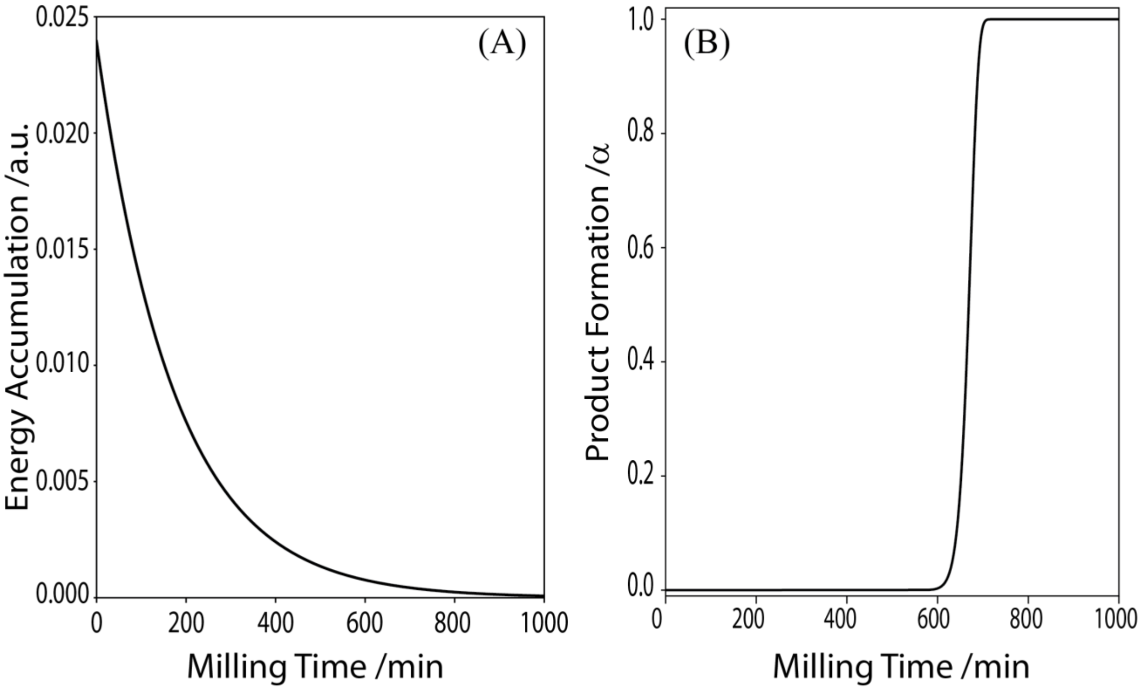

Figure S3: Kinetic model for 15 Hz ball mill neat milling, employing Equation S1 for energy accumulation rate. The per-second energy accumulation is given (A) alongside rate of product formation (B). $E_0 : \tau = 17.48$, as used for modelling throughout the main text.

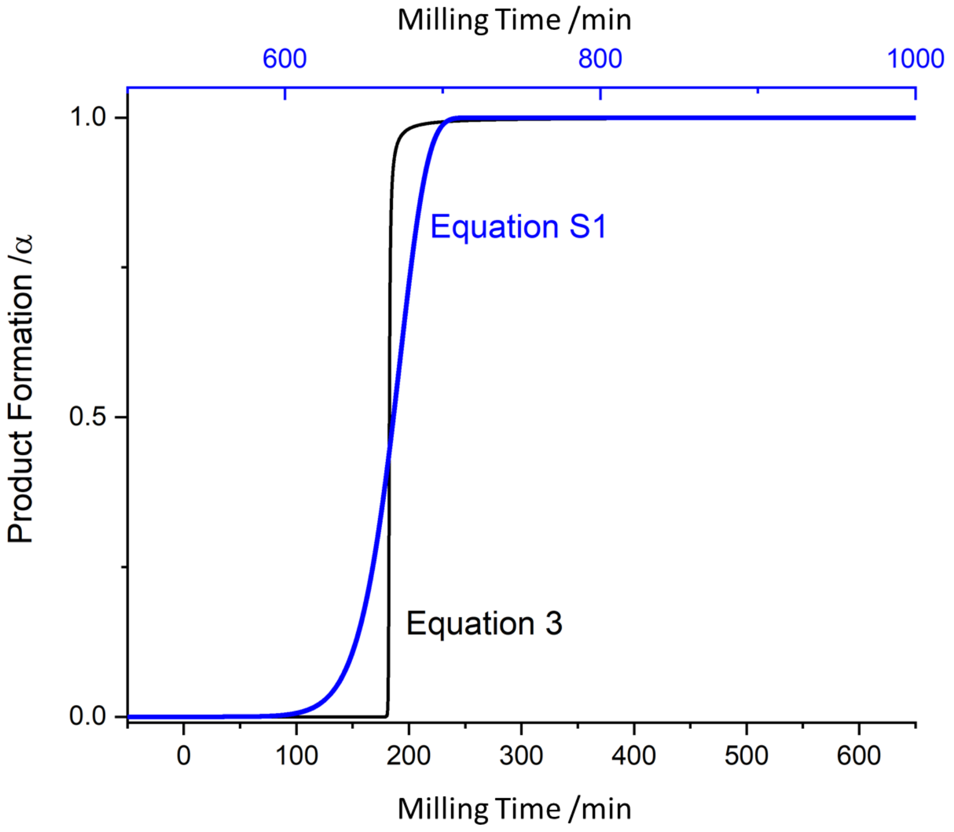

Figure S4: Comparison of the 15 Hz neat milling model using (black) Equation 3 and (blue) Equation S1. The sigmoidal trend is best reproduced by Equation S1, although the onset time is grossly overestimated. $E_0 : \tau = 17.48$ in both cases, as was used for modelling in the main text.

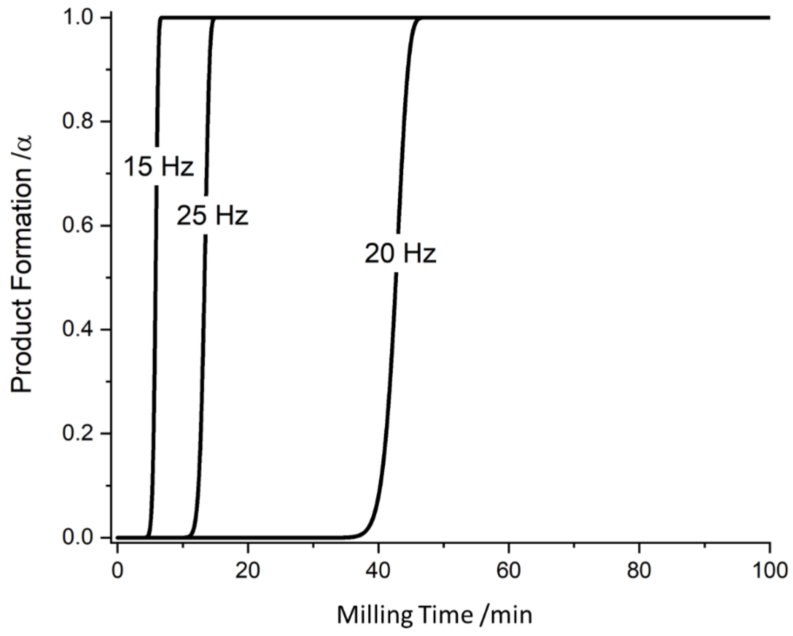

Figure S5: Kinetic model for 20, 25 and 30 Hz neat milling, employing Equation S1 for energy accumulation rate. $E_0 : \tau = 17.48$ as was used in the main text.

Further work is required to determine which mechanism (Equation 3, Equation S1 or otherwise) is mechanistically more reasonable. Given that each equation offers some added benefit over the other, it seems an intermediary equation is necessary. While Equation S1 does appear to better reproduce the on-set times of higher frequency milling (Table S1, Figure S5 and Figure S6), and indeed offers more sigmoidal character, it grossly overestimates the 15 Hz milling experiment as shown in Figure S4 and Figure S6. Thus at present, without additional work, Equation 3 (with the addition of a short mixing time) continues to yield the best overall results.
Both Equation 3 and Equation S1 yield similar relative onset times (*i.e.* ratio of onset time at each frequency – a measurement of non-linearity) at each milling frequency, with the notable exception being 15 Hz milling. As noted in the main text, this is possibly due to the non-ideal case associated with using 2 milling balls. One may also consider energy accumulation depends on the type of defect (and hence on the energy of each individual impact). We further note that, at the microscopic level, one must additionally segregate steps in which impact energy accumulates, and in which its relaxation leads to other effects, such as deagglomeration. Such additional considerations require more in-depth understanding of the system.

Table S1: Onset times for mechanochemical reaction according to Equation 3 and Equation S1. Note that values for Equation 3 include an addition period of pre-mixing.

Frequency /Hz	Onset Time Equation 3, with mixing /min	Onset Time Equation S1 /min
15	185	570
20	28	37
25	17	11
30	13	5

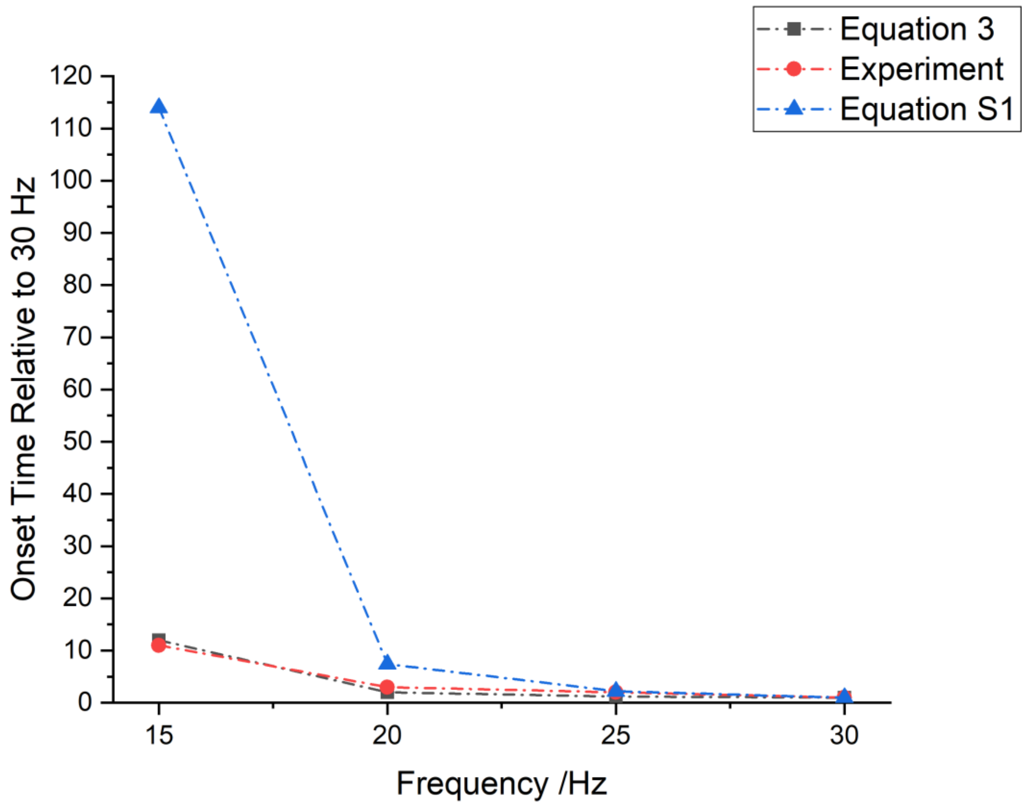

Figure S6: Comparison of relative on-set milling times at the four milling frequencies. All onset times are normalized to the fastest (*i.e.* 30 Hz) onset times, as indicated in Table S1

S5 Effect of Mixing

Prior to any multi-phase chemical reaction, reactant phases must come into contact. In solid state reactions, this occurs by formation of heterogeneous solid-solid contacts. As such, Scheme 1 in the main text contains a mixing/demixing equilibrium. The complexity of mixing[2] ensures that no analytical equations describe it. One must account for oscillations in particle size, density, shape, surface interactions, rheological changes, amongst other physical parameters. Experimentally, however, mixing under ball milling conditions tends to adhere to an exponential trend.[3] We have therefore employed such a model here. We furthermore make the assumption that homogeneous contacts (e.g. A/A and B/B) contacts are equally probably as heterogeneous contacts (A/B). That is to say that no preferential surface interactions occur in either case. Hence, the rate of mixing and demixing is taken to be equal in our simplified model. This is a reasonable assumption in the absence of charged surfaces.

If it is assumed that the mixing is itself the rate-limiting step, hence $k_1 \ll k_1{}'$, Figure S7, is obtained. Note that this condition is also descriptive of cases in which de-mixing is substantially more favourable than mixing. Qualitatively, it is seen that in such cases there is only a negligible contribution to onset time for the reaction. Instead, mixing has the effect of reducing the severity of product accumulation, once the energy barrier has been surmounted. Thus, while mixing may play an important role in tuning the slope of reaction rate, it does not alone describe the sigmoidal trend, with long induction period, observed experimentally.

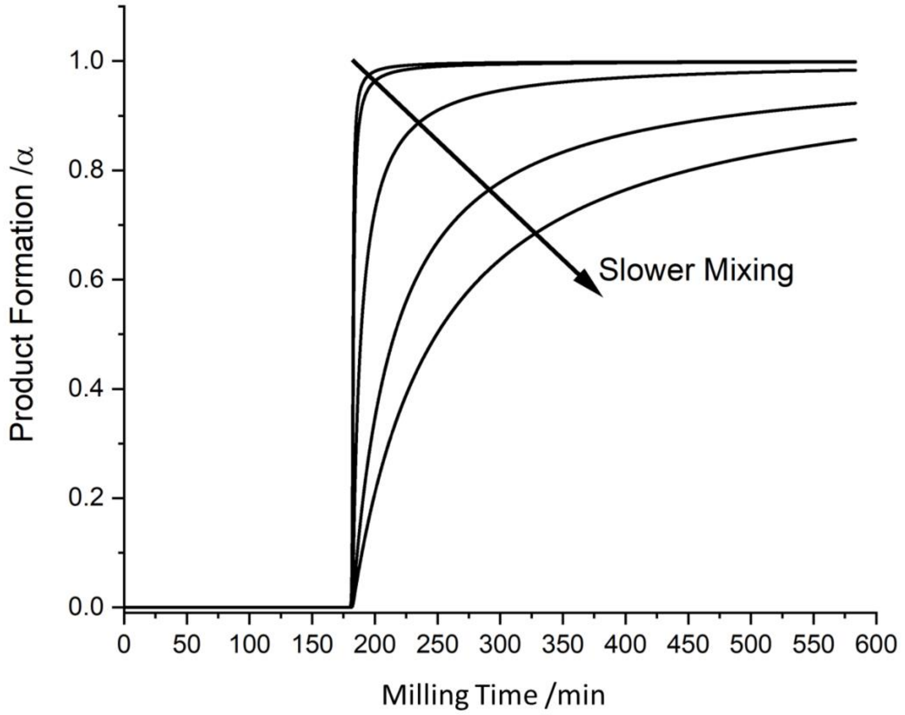

Figure S7: Kinetic curves modelled for 15 Hz milling under ball mill NG conditions. The rate limiting step is taken to be mixing (k_1 in the main text). Rate constants k_1 are chosen as 0.1 s^{-1}, 0.01 s^{-1}, 0.005 s^{-1}, 0.001 s^{-1}, 0.0005 s^{-1}.

Conceptually it is not surprising to find that mixing plays a negligible roll in controlling the onset time. Regardless of the mixing rate, once sufficient energy is present in the system, reaction will occur at heterogeneous contacts. For this reason, it follows that such large induction periods, as observed in this study, cannot result solely from mixing phenomena. If mixing were to be taken to explain this phenomenon, one must simultaneously assume that *no* reaction occurs during mixing. This is not reasonable unless one also considers the lack sufficient energy for reaction.

S6 References

1. Butyagin, P.Y. (1971) Kinetics and Nature of Mechanochemical Reactions. *Russ. Chem. Rev.*, **40**, 901.
2. Poux, M., Fayolle, P., Bertrand, J., Bridoux, D., and Bousquet, J. (1991) Powder mixing: Some practical rules applied to agitated systems. *Powder Tech.*, **68** (3), 213–234.
3. Massol-Chaudeur, S., Berthiaux, H., and Dodds, J. a. (2002) Experimental study of the mixing kinetics of binary pharmaceutical powder. *Chem. Eng. Sci.*, **57** (19), 4053–4065.
